# Exploring the Synthesis, Anti-Inflammatory and Anti-Tumor Potential of 4-Maleimidylphenyl-Hydrazide Derivatives

**DOI:** 10.3390/molecules30204035

**Published:** 2025-10-10

**Authors:** Francis Cloutier, Alexis Paquin, Maude Cloutier, Yassine Oufqir, Laurie Fortin, Julie Girouard, Heidar-Ali Tajmir-Riahi, Carlos Reyes-Moreno, Gervais Bérubé

**Affiliations:** 1 Laboratoire de Recherche en Chimie Médicinale (LRCM) et Groupe de Recherche en Signalisation Cellulaire (GRSC), Département de Chimie, Biochimie et Physique, Université du Québec à Trois-Rivières, Trois-Rivières, QC G8Z 4M3, Canada; 2 Laboratoire de Recherche en Oncologie et Immunobiologie (LROI) et Groupe de Recherche en Signalisation Cellulaire (GRSC), Département de Biologie Médicale, Université du Québec à Trois-Rivières, Trois-Rivières, QC G8Z 4M3, Canada

**Keywords:** hydrazide derivatives, anti-inflammatory, bladder cancer, MTT assay, nitric oxide

## Abstract

The design of innovative compounds displaying anti-inflammatory activity in oncological context is a subject of great interest in drug development. It has been proved that a pro-inflammatory microenvironment which accelerates cancer growth and cellular differentiation is often present in malignant bladder tumor. In earlier work, we reported the synthesis of *p*-aminobenzoic acid derivatives that act as anti-inflammatory compounds able to inhibit the pro-inflammatory markers present in bladder cancer microenvironment. **DAB-1** rapidly emerged as an effective lead candidate in this investigation, with its ability to shrink by 90% in 25 days the size of human bladder cancer tumors in an ectopic mouse model. This manuscript discloses the synthesis of 23 new hydrazide derivatives of **DAB-1** and reports their in vitro and in vivo biological evaluation. It was discovered that most of the new compounds are essentially nontoxic against RAW 264.7 cells, as evaluated by an MTT assay. Anti-inflammatory activity of the new derivatives was investigated by evaluation of their impact on cellular nitric oxide production, measured by a Griess assay. Some compounds did significatively inhibit nitric oxide production much more effectively than the original **DAB-1**. Striking activity of **14**, which is around four times more potent than **DAB-1**, promotes this derivative as new lead compound in this study. The study of these analogs reveals that a phenolic/anisole core is a key component to achieve high biological activity. Furthermore, mice models of acute inflammation and invasive BCa tumors were used to assess the in vivo impact of derivative **14**, and it was found that this compound does reduce inflammation in these mice, possess similar anti-inflammatory activity but higher anti-tumoral activity compared to **DAB-1** with no apparent signs of toxicity.

## 1. Introduction

Despite the many efforts of the scientific community, cancer remains a serious challenge to global health [1]. In the United States alone, cancer is the second-leading cause of death for people under 85 years old, accounting for more than 611,720 deaths in 2024 [2]. It is estimated that 16,840 of those deaths are directly attributed to urinary bladder cancer (BCa), a type of neoplasm known to cause severe discomfort to the patients and whose surgical removal can significantly impair one’s life quality [3]. BCa can be muscle invasive and afflicts men approximately 4 times more often than women, which is partly due to the role of sexual hormones in this cancer development [4,5]. Moreover, this type of cancer is known to metastasize if not treated early, and the survival rate for stage IV BCa is around 7%, which underscores the need to develop new therapies that rapidly curb the growth of these malignant tumors [6,7].

A prime characteristic of BCa is the presence of an inflammatory microenvironment, which directly influences the proliferation of cancer cells. Not only inflammatory markers such as TNFα, NF-κB, and Interleukin-I are known to promote BCa’s growth, but chronic inflammation of the urinary tract has also been found to increase the risk of BCa and is associated with worse prognoses in both men and women [8,9]. Inflammation and BCa are so closely related that regular use of nonsteroidal anti-inflammatory drugs has been shown to decrease the risk of contracting BCa [10]. These observations prompted us to design new compounds displaying anti-inflammatory properties against BCa cells. Such molecules are expected to repress the pro-inflammatory tumor microenvironment and to speed up tissue recovery.

**Figure 1:**
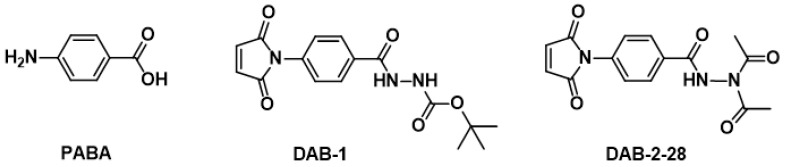
Structure of **DAB-1** and **DAB-2-28** made from the natural product *p*-aminobenzoic acid (PABA).

*p*-Aminobenzoic acid (PABA) is an important cofactor that enables folic acid production in bacteria and possesses mild anti-inflammatory activity [11]. Multiple research programs have already established the relevance of PABA and PABA analogs in a medicinal context, as these compounds can exert a wide range of effects on biological systems [12,13,14]. Our group previously reported the synthesis of **DAB-1**, a synthetic derivative of PABA functionalized with a hydrazinamide and a maleimide moiety (Figure 1) [15]. **DAB-1** was shown to possess good anti-inflammatory activity against various cell lines and relatively low toxicity, albeit in the presence of the electrophilic maleimide. Both the hydrazinamide and the maleimide fragments were found to be essential for retention of the activity, and it was also established that interactions of **DAB-1** with the transport protein happen mainly through ionic interactions [16]. Furthermore, in vivo investigation revealed that **DAB-1** exerts its anti-inflammatory effects through the inhibition of TNFα/NFκB and iNOS/NO pathways [17]. In subsequent medicinal chemistry endeavors, we sought to amplify the biological potential of **DAB-1** through acetylation of the hydrazinamide right-hand side, which resulted in **DAB-2-28**, a compound displaying enhanced anti-inflammatory and lower toxicity compared to the original **DAB-1**, both in vitro and in vivo [17,18]. More recently, we reported the synthesis of polyacylated analogs of **DAB-2-28** and uncovered that the substitution pattern on the hydrazinamide can significantly impact the biological potential of these analogs [19].

With these results in hand, it became clear that the hydrazinamide group can be tuned to effectively modulate the anti-inflammatory properties of these compounds. Herein, we undertook the synthesis of a series of hydrazide derivatives with the aim to further refine the biological potential of our PABA analogs. The hydrazide moiety is a functional group of great interest in medicinal chemistry. It is present in many examples of lead compounds displaying a wide range of relevant properties, including anti-inflammatory activity [20]. Hydrazides exhibit good in vivo hydrolytic stability, and the NH alpha to the carbonyl acts as a powerful hydrogen bond donor/acceptor, which could enhance the pharmacodynamic of our derivatives [21]. Our overarching objective with this program is to explore the general potential of these hydrazides to generate new nontoxic candidates possessing in vitro and in vivo anti-inflammatory activity in the context of BCa.

## 2. Results and Discussion

### 2.1. Design and Chemistry

**Scheme 1:**
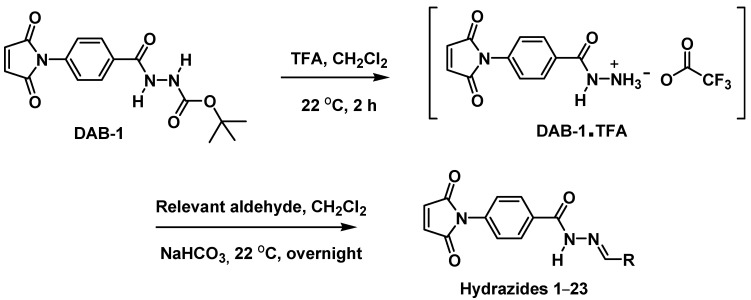
Synthesis of hydrazides from **DAB-1**.

The 4-maleimidylphenyl-hydrazide molecules were obtained by reacting the known **DAB-1** molecule with a variety of aldehydes. Two methods were used to synthesize these molecules, which depended on the solubility of the resulting hydrazides (Scheme 1). Insoluble hydrazides were isolated by simple filtration with a sequence of dichloromethane and water, as described in method A. Soluble hydrazides were extracted with appropriate solvent, the organic phase dried and evaporated to the desired material, as described in method B. By means of these methods, a series of 23 hydrazides were made and characterized by infrared spectroscopy (IR), nuclear magnetic resonance spectroscopy (proton and carbon NMR), and by high resolution mass analysis (Table 1).

### 2.2. Biological Results

#### 2.2.1. Antiproliferative Activity on Macrophage Cell Lines

The antiproliferative activity of compounds **1–23** was measured using the MTT assay on RAW 264.7 cells, a malignant macrophage cell line widely used as an in vitro benchmark in the development of anti-inflammatory drugs [22]. The cells were pretreated for 30 min with the vehicle (0.1% DMSO in PBS) or with **DAB-1** or compounds **1–23** at 15 µM and then washed and incubated for 24 h. The dose concentration at 15 μM was chosen on the basis that at this dose, **DAB-1** efficiently inhibits biological processes (cell signaling activation and NO production) without affecting cell viability [15,18,19]. The cultured cells were recovered, and the relative number of viable, proliferating cells was subsequently evaluated by an MTT assay.

The results demonstrate that most compounds retained the innocuous nature of **DAB-1**, with most of them being nontoxic against RAW 264.7 cells (Table 2). Only the unsubstituted phenyl **1** (41 ± 2% cell viability), the styrene derivative **2** (67 ± 2% cell viability), and the *o*-phenol **6** (35 ± 1% cell viability) exhibit toxic cellular behaviors at the tested concentration, along with a few analogs bearing electron-rich aromatic rings, which showed mild toxicity. Contrarily, every derivative possessing electron-deficient aryl rings was found to be non-toxic in these conditions. These divergences might be due to off-target actions of the former or enhanced metabolism rate for these analogs, which could generate higher concentrations of potentially toxic by-products. Nevertheless, most compounds were found not to compromise cellular health at 15 µM.

#### 2.2.2. Evaluation of Anti-Inflammatory Properties of the Structural Components of **DAB-1** and Its Derivatives **1–23**

Following cellular toxicological evaluation, we sought to assess the ability of our compounds to reduce inflammation in cells. To this end, RAW 264.7 cells were pretreated for 30 min with either the vehicle (0.1% DMSO in PBS) or with **DAB-1** and compounds **1–23** at 15 µM and then washed and incubated for 24 h. The culture media were recovered, and NO production was subsequently quantified by the Griess reagent. Results indicate a major enhancement of the anti-inflammatory properties of some hydrazides compared to former **DAB-1** (Table 3). The most notable result arises from **14**, which displays a normalized NO inhibition level of over 84%, a four-fold potency improvement over **DAB-1,** which showed 19% inhibition at the tested concentration. Moreover, we were delighted to observe that **14** is nontoxic towards the cells at this concentration, which motivates us to instigate this derivative as a new prospective candidate in this study. Interestingly, closely related compounds **15** and **16** were essentially inactive, displaying 1% and 0% NO inhibition, respectively. This emphasizes that the substitution pattern of the methoxyphenol core is of paramount importance in achieving high biological potency, and that the activity of **14** is not simply due to non-specific mechanisms but is tied to specific interactions determined by the study of these analogs. Hydrazide **14** may acquire its enriched anti-inflammatory activity from H-bonding interactions involving the oxygen atoms on the aromatic ring, or via π interactions with relevant residues. Furthermore, hydrazide **10**, which also possesses a methoxy group in the meta position, displays significant NO inhibition (36%), and so does its demethylated analog **7** (22%), highlighting the crucial role of the oxygen atom at this position.

Other interesting results come from compounds **17** and **18**, which, respectively, show normalized NO inhibition levels of 71% and 75%. These compounds show outstanding anti-inflammatory potential and were not found to be harmful to RAW 264.7 cells, which thereby brings special interest to furan-type hydrazide in this current project. Moreover, the anti-inflammatory properties of 4-nitrophenyl-furan derivative **22** (48% NO inhibition) and pyridine derivative **23** (44% NO inhibition) expand the scope of possible aromatic cores able to induce positive biological responses. Additionally, it is interesting to note that between compounds **3**, **4**, and **5**, only the *m*-tolyl derivative **4** and the *p*-tolyl derivative **5** were active, with both compounds possessing 39% normalized NO inhibition and the *o*-tolyl derivative **3** being essentially inert. This effect in the methyl substitution pattern of the aryl ring might arise from preferential docking of compounds **4** and **5** to the relevant biological target, or it could also be due to differences in the in vitro stability of the *o*-tolyl **3**. Further encouraging results come from compounds **10** and **11**, which, respectively, show normalized NO inhibition levels of 36% and 35%, a considerable upswing compared to previous DAB generations. Unfortunately, derivative **10** exhibits slightly toxic behaviors at 15 µM, which prevents further investigation of this compound without addressing toxicological problems beforehand.

#### 2.2.3. In Vivo Evaluation of Anti-Inflammatory and Anti-Tumoral Properties of Hydrazide Derivative **14**

**Figure 2:**
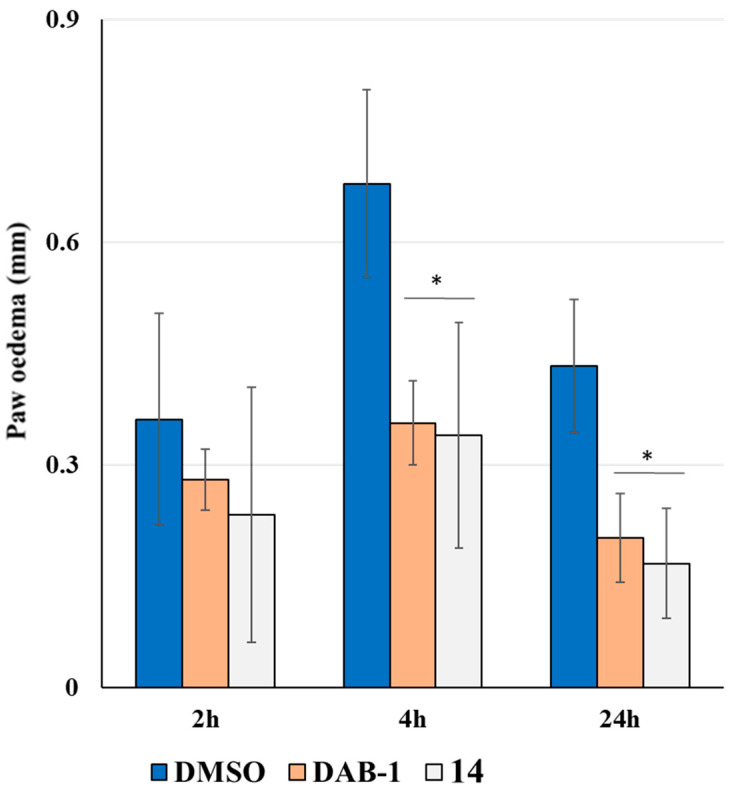
Effect of **DAB-1** and **14** in an acute inflammation model. Female CD-1 mice (5 mice/group) received an intraperitoneal (i.p.) injection of DMSO, **DAB-1,** and **14** at day −1. Carrageenan was injected at day 1 in the right paw, and then the mice were treated with DMSO (control) or DABs 1 h before and 24 h later. Paw thickness was then measured using a micrometer at t = 2 h, t = 4 h, and t = 24 h. * *p* < 0.01 compared to control.

Considering the phenomenal in vitro anti-inflammatory properties and low toxicity of **14**, we sought to evaluate if the observed activities transmute in vivo. Prior to animal testing, the impact of **14** on cellular proliferation was re-evaluated at multiple concentrations on RAW 264.7 cells to confirm a dose-dependent effect and to attest that its IC_50_ is similar to **DAB-1**, thus validating a high toxicity threshold for this compound (Figure S1). Subsequently, an acute inflammation model was performed to assess the in vivo potency of **14**. CD-1 female mice were pretreated with DMSO (control), **DAB-1**, or **14,** 24 h, and 1 h before an edema on the right paw was induced by a subplantar carrageenan injection. Intraperitoneal injections of DMSO and the two compounds were once again performed 24 h later, and paw thickness was measured at various times using a micrometer. The compiled data clearly demonstrate that **DAB-1** and derivatives **14** are both superior to control to alleviate acute inflammation in CD-1 mice (Figure 2). However, the results indicate that **DAB-1** and **14** have similar anti-inflammatory activity, even though the new hydrazide derivative was significantly more potent in vitro. Those discrepancies might be due to pharmacokinetic issues related to **14**, like drug distribution or metabolic liabilities. Nevertheless, both compounds did show a significant anti-inflammatory effect in vivo, and they did not seem to affect normal mouse development, as no variation in body weight was observed. The body weight at the endpoint was 29.9 ± 2.5 in the DMSO group, 30.8 ± 3.2 in the **DAB-1** group, and 30.1 ± 2.8 in the **14** group.

**Figure 3:**
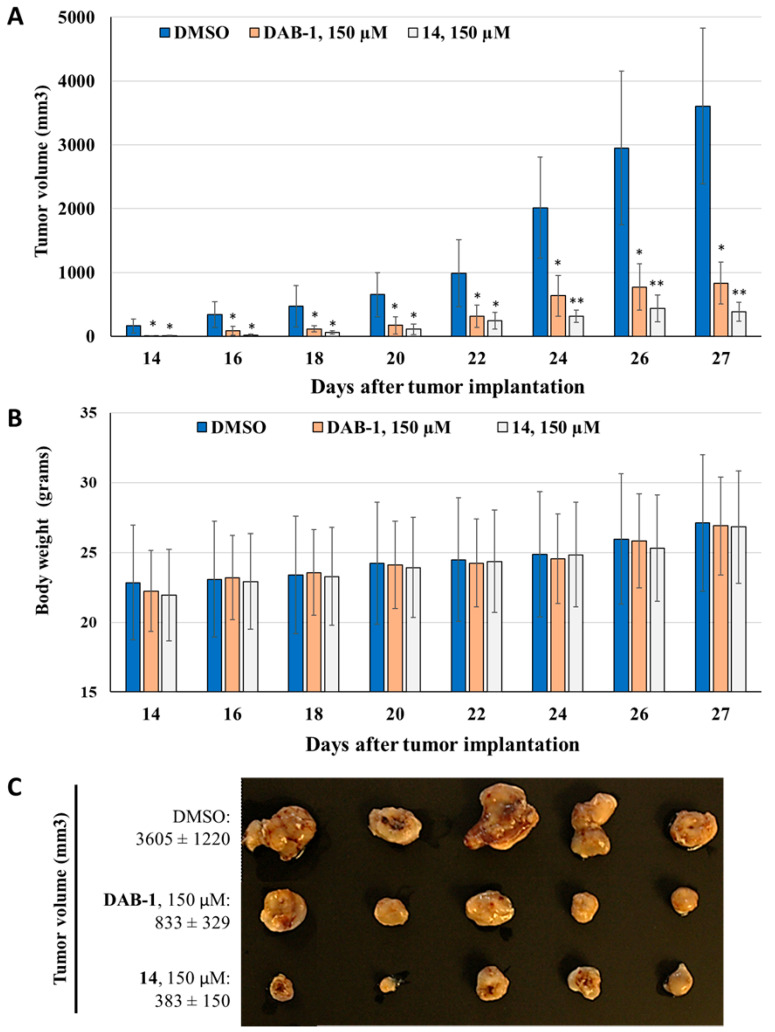
Molecular targeting of ectopic MB49-I tumors with **DAB-1** and **14**. (**A**) Graphical representation showing the effects of **DAB-1** and **14** on the development of MB49-I tumors subcutaneously implanted in male mice (n = 8); volume is expressed in mm^3^ and corresponds to the mean for each group of mice. (**B**) Body weight measurements in different treated subgroups of male mice; data is expressed in grams and correspond to the mean for each group of mice. Error bars represent ± SEM. * *p* < 0.05 and ** *p* < 0.01 denote significant differences compared with the vehicle control group. (**C**) Representative images showing MB49-I tumors that were surgically recovered at endpoint (day 27 after tumor implantation).

To investigate the in vivo efficacy of **DAB-1** and **14** to target subcutaneous MB49-I tumors, tumor-bearing mice received i.p. treatment with vehicle (0.1% DMSO in PBS; control group) or solutions of **DAB-1** and **14**, both at 150 μM. The molecules were injected on day 6 post-inoculation. Assessment of tumor growth kinetics indicated that tumors from mice treated with **DAB-1** and **14** grew at a significantly lower rate than tumors from mice in the control group (Figure 3A). The mice were sacrificed on the 27th day after inoculation when the tumor had reached their limit points. A significant reduction in tumor growth was obvious starting at day 14 post-implantation and was maintained until the endpoint at day 27 post-implantation (Figure 3A) without affecting the mice’s body weight throughout the therapy (Figure 3B). However, results showed that molecular therapy with **14** was more efficient in inhibiting tumor growth than **DAB-1**, the inhibition rates being evaluated at 89% with **14** versus 77% with **DAB-1** after 27 days of targeted therapy (Figure 3A). Tumor tissue macroscopic analysis at the endpoint confirmed that **14** was more efficient than both controls, DMSO (no treatment) and **DAB-1**, in inhibiting the growth of ectopic MB49-I tumors (Figure 3C).

## 3. Materials and Methods

### 3.1. Biological Methods

#### 3.1.1. Evaluation of NO Production and Cell Viability/Proliferation

Cell viability and NO production assays were performed on cultured cells and their corresponding cell culture-derived supernatants. Briefly, murine RAW 264.7 cells were seeded into a 96-well plate (7.5 × 10^3^ cells/well) and cultured for 24 h at 37 °C and 5% CO_2_. Cells were pretreated with 0.1% DMSO (control), **DAB-1,** and its derivatives at 15 μM for 30 min, as described [15]. Then, the cells were washed and left either unstimulated or stimulated with cytokines IFN-γ (5 ng/mL) and TNF-α (25 ng/mL) for a period of 24 h. At the end of the stimulation period, cells and supernatants were harvested and prepared for cell viability and NO measurements. As previously described, NO production and cell viability/proliferation were measured by the Griess reagent and the MTT reagent methods, respectively [19]. Data is presented as a percentage of control (0.1% DMSO) for NO production and cell viability. Data normalization for the levels of NO production and NO production inhibition was computed taking into account the relative number of viable cells for each condition.

#### 3.1.2. Acute Inflammation Study in Mice

The influence of DAB molecules on acute inflammatory response was assessed in vivo using the carrageenan-induced paw edema model as described [23]. Briefly, 5 CD-1 mice were treated with intraperitoneal (i.p.) injections of 100 μL of vehicle (0.1% DMSO in PBS), **DAB-1** (150 μM), and **14** (150 μM) 24 h and 1 h before administering carrageenan. Paw edema was induced in all animals by subplantar injection (50 μL) of carrageenan (1% *v*/*v*) into the paws of the right hind legs of mice. Then, the vehicle and the DAB molecules, at 150 μM, were administered 24 h after the subplantar injection of carrageenan. To verify the presence of edema, paw thickness was measured using a micrometer 2 h, 4 h, and 24 h after the carrageenan injection. To evaluate the normal development of mice, the body weight of each animal was recorded 1 h before and 24 h after carrageenan subplantar injection. This animal study was conducted in accordance with the rules of the Animal Care and Use Committee of the Université du Québec à Trois-Rivières (Trois-Rivières, Canada).

#### 3.1.3. Growth Kinetics and Treatment of Murine Bladder Tumors

This in vivo study was planned to investigate the impact of DAB molecules on tumor growth in an ectopic (subcutaneous) model of bladder cancer (BCa), which was developed using the murine MB49-I BCa cells, as described [18,24]. Briefly, tumor cells (5 × 10^4^ in 100 μL PBS) were injected subcutaneously into the left flank of 4-week-old C57Bl/6 male mice. At day 4 post tumor implantation, when the tumor reached a size of approximately 10 mm^3^, each group of mice (n = 8) received i.p. injections with 100 μL solution of either 0.1% DMSO in PBS (group 1), **DAB-1** at 150 μM (group 2), or **14** at 150 μM (group 3). I.p. injections were repeated every 2 to 3 days for three weeks. Growth rates of the subcutaneous tumors were monitored by measuring the tumor size every 3–4 days using a digital caliper. The volume of the tumor was calculated using the following formula: length × (width)^2^ × 0.52. Mice were examined daily for signs of tumor progression, including visible signs of tumor growth, apathy, or weight loss (2–3 g overnight), and were sacrificed when any of these signs became apparent. To directly evaluate the impact of treatment on tumor growth, ectopic MB49-I tumors were surgically recovered at the endpoint. To evaluate the normal development of mice, the body weight of each animal was recorded on the same days that the tumor volume was monitored. Mice were sacrificed 27 days after inoculation of MB49-I cells. The Animal Care and Use Committee of the Université du Québec à Trois-Rivières (Trois-Rivières, Canada) has approved all animal procedures.

#### 3.1.4. Statistical Analyses

Data obtained from in vitro experiments are presented as means ± SEM from at least three independent experiments performed in triplicate. The difference between groups was evaluated by two-way ANOVA followed by a Bonferroni post-test, as described in [18]. In the preclinical model of BCa, the difference between groups was evaluated by unpaired two-tailed Student’s t-test or two-way ANOVA followed by Bonferroni post-test, as described [24]. Statistical differences were significant at the value of *p* < 0.05 (* *p* < 0.05, ** *p* < 0.01).

### 3.2. Chemistry

Anhydrous reactions were performed under an inert atmosphere of nitrogen. The starting material, reactant and solvents were obtained commercially and were used as such or purified and dried by standard means [25]. Organic solutions were dried over magnesium sulfate (MgSO_4_), filtered and evaporated on a rotary evaporator under reduced pressure. All reactions were monitored by UV fluorescence. Commercial TLC plates were Sigma T 6145 (polyester silica gel 60 Ǻ, 0.25 mm). Flash column chromatography was performed according to the method of Still et al. on Merck grade 60 silica gel, 230–400 mesh [26]. All solvents used in chromatography were distilled.

The infrared spectra were taken on a Nicolet Impact 420 FT-IR spectrophotometer. Mass spectral assays were obtained using an MS model 6210, Agilent technology instrument. The high-resolution mass spectra (HRMS) were obtained by TOF (time of flight) using ESI (electrospray ionization) using the positive mode (ESI+) (Université du Québec à Montréal). Nuclear magnetic resonance (NMR) spectra were recorded on a Varian 200 MHz NMR apparatus. Samples were dissolved in deuterated dimethyl sulfoxide (DMSO-*d*_6_) for data acquisition, using the residual solvent signal as internal standard (dimethyl sulfoxide, δ 2.50 ppm for ^1^H NMR and δ 39.52 ppm for ^13^C NMR). Chemical shifts (δ) are expressed in parts per million (ppm), and the coupling constants (*J*) are expressed in hertz (Hz). Multiplicities are described by the following abbreviations: s for singlet, d for doublet, t for triplet, m for multiplet, and br s for broad singlet.

Note: The products **1–23** were obtained as amorphous solids and decomposed upon heating. Hence, no melting points are reported in this publication.

#### 3.2.1. Synthesis of 4-Maleimidylphenyl-Hydrazide Molecules

##### Synthesis of Tert-Butyl 2-(4-(2,5-Dioxo-2,5-Dihydro-1H-Pyrrol-1-yl)Benzoyl)Hydrazinecarboxylate (**DAB-1**)

**DAB-1** was synthesized using a procedure reported in earlier manuscripts [15]. The ^1^H-NMR and ^13^C-NMR spectral data are given to facilitate comparison with the novel molecules reported in this manuscript. ^1^H NMR (200 MHz, DMSO-*d*_6_, δ ppm): 10.25 and 8.93 (2 × s, 2H, 2 × NH), 7.92 and 7.46 (2 × d, *J* = 8.4 Hz, 4H, aromatic), 7.20 (s, 2H, maleimide), 1.41 (s, 9H, 3 × CH_3_); ^13^C NMR (50 MHz, DMSO-*d*_6_, δ ppm): 170.1, 165.8, 155.9, 135.3, 135.0, 131.9, 128.4, 126.7, 79.7, 28.5.

Synthesis of hydrazide derivatives

Procedure A—for insoluble final products

**DAB-1** (1 eq.) was dissolved in dichloromethane (0.3 M), and trifluoroacetic acid (10 eq.) was added. The solution was stirred at 22 °C for a period of 2 h and then evaporated under vacuum to give intermediate **DAB-1**. **TFA** is a viscous oil. It was dissolved in dichloromethane (0.15 M) and cooled in an ice bath. Relevant aldehyde (1 to 3 eq.) along with sodium bicarbonate (1 eq.) were added to the mixture, which was then stirred overnight at 22 °C. Afterwards, hexane was added to complete the precipitation, and the suspension was filtered on a fritted glass filter under vacuum. The solid product was washed with dichloromethane and water before being dried under vacuum to give the desired material.

Procedure B—for soluble final products

**DAB-1** (1 eq.) was dissolved in dichloromethane (0.3 M), and trifluoroacetic acid (10 eq.) was added. The solution was stirred at 22 °C for a period of 2 h and then evaporated under vacuum to give intermediate **DAB-1**. **TFA** is a viscous oil. It was dissolved in dichloromethane (0.15 M) and cooled in an ice bath. A relevant aldehyde (1 to 3 eq.), along with sodium bicarbonate (1 eq.), was added to the mixture, which was then stirred overnight at 22 °C. Afterwards, the solution was evaporated, resuspended in ethyl acetate, and washed 5 times with distilled water. The organic phase was dried on magnesium sulfate, filtered, and evaporated to dryness to give the desired material.

##### Synthesis of *N*-Benzylidene-4-(2,5-Dioxo-2,5-Dihydro-1*H*-Pyrrol-1-yl)Benzohydrazide (**1**)

Procedure A was followed with 101.3 mg (0.31 mmol) of **DAB-1** and 100 μL (0.98 mmol) of benzaldehyde, which yielded 60.4 mg (62%) of the desired material **1**. IR (ν, cm^−1^): 3481 (N-H), 3165–3011 (Csp^2^-H), 1710 (C=O), 1685 (C=O), 1609 (C=C); ^1^H NMR (200 MHz, DMSO-*d*_6_, δ ppm): 11.92 (s, 1H), 8.47 (s, 1H), 8.02 (d, *J* = 8.3 Hz, 2H), 7.75 (m, 2H), 7.50 (m, 5H), 7.23 (s, 2H, maleimide); ^13^C NMR (50 MHz, DMSO-*d*_6_, δ ppm): 169.64, 162.50, 148.02, 134.85, 134.49, 134.26, 132.36, 130.14, 128.86, 128.26, 127.12, 126.27; ESI+ HRMS: (M+H)+ calculated for C_18_H_14_N_3_O_3_ = 320.1030; found = 320.1028.

##### Synthesis of 4-(2,5-Dioxo-2,5-Dihydro-1*H*-Pyrrol-1-yl)-*N*-(3-Phenyallylidene)Benzohydrazide (**2**)

Procedure A was followed with 106.0 mg (0.31 mmol) of **DAB-1** and 40 μL (0.32 mmol) of *trans*-cinnamaldehyde, which yielded 80.6 mg (76%) of the desired material **2**. IR (ν, cm^−1^): 3468 (N-H), 3083–3035 (Csp^2^-H), 1700 (C=O), 1646 (C=O), 1607 (C=C); ^1^H NMR (200 MHz, DMSO-d6, δ ppm): 11.82 (s, 1H), 8.23 (s, 1H), 7.99 (d, *J* = 8.1 Hz, 2H), 7.69–7.31 (m, 7H), 7.20 (s, 2H, maleimide), 7.06 (s, 2H); ^13^C NMR (50 MHz, DMSO-*d*_6_, δ ppm) ^13^C NMR (50 MHz, DMSO-*d*_6_, δ ppm): 169.78, 162.60, 150.23, 139.48, 135.98, 134.96, 134.60, 132.49, 132.43, 129.01, 128.40, 127.27, 126.42, 125.67; ESI+ HRMS: (M+H)+ calculated for C_20_H_16_N_3_O_3_ = 346.1186; found = 346.1178.

##### Synthesis of 4-(2,5-Dioxo-2,5-Dihydro-1*H*-Pyrrol-1-yl)-*N*-(2-Methylbenzylidene)Benzohydrazide (**3**)

Procedure A was followed with 100.3 mg (0.30 mmol) of **DAB-1** and 36 μL (0.31 mmol) of *o*-tolualdehyde, which yielded 57.8 mg (57%) of the desired material **3**. IR (ν, cm^−1^): 3474 (N-H), 3182–3024 (Csp^2^-H), 2856 (Csp^3^-H), 1726 (C=O), 1711 (C=O), 1655 (C=C); ^1^H NMR (200 MHz, DMSO-*d*_6_, δ ppm): 11.89 (s, 1H), 8.76 (s, 1H), 8.03 (d, *J* = 8.4 Hz, 2H), 7.86 (d, *J* = 7.0 Hz, 1H), 7.53 (d, *J* = 8.4 Hz, 2H), 7.41–7.22 (m, 5H), 2.46 (s, 3H); ^13^C NMR (50 MHz, DMSO-*d*_6_, δ ppm): 169.63, 162.32, 146.59, 136.90, 134.85, 134.48, 132.36, 132.24, 130.88, 129.82, 128.20, 126.26, 126.21, 125.86, 19.03; ESI+ HRMS: (M+H)+ calculated for C_19_H_16_N_3_O_3_ = 334.1186; found = 334.1180.

##### Synthesis of 4-(2,5-Dioxo-2,5-Dihydro-1*H*-Pyrrol-1-yl)-*N*-(3-Methylbenzylidene)Benzohydrazide (**4**)

Procedure A was followed with 100.4 mg (0.30 mmol) of **DAB-1** and 36 μL (0.31 mmol) of *m*-tolualdehyde, which yielded 90.8 mg (90%) of the desired material **4**. IR (ν, cm^−1^): 3460 (N-H), 3170–3090 (Csp^2^-H), 2957 (Csp^3^-H), 1710 (C=O), 1663 (C=O), 1610 (C=C); ^1^H NMR (200 MHz, DMSO-*d*_6_, δ ppm): 11.91 (s, 1H), 8.42 (s, 1H), 8.02 (d, *J* = 8.4 Hz, 2H), 7.59–7.49 (m, 4H), 7.44–7.20 (m, 4H), 2.37 (s, 3H); ^13^C NMR (50 MHz, DMSO-*d*_6_, δ ppm): 169.64, 162.48, 148.05, 138.10, 134.85, 134.22, 132.37, 130.86, 128.76, 128.26, 127.38, 126.26, 124.56, 20.90; ESI+ HRMS: (M+H)+ calculated for C_19_H_16_N_3_O_3_ = 334.1186; found = 334.1182.

##### Synthesis of 4-(2,5-Dioxo-2,5-Dihydro-1*H*-Pyrrol-1-yl)-*N*-(4-Methylbenzylidene)Benzohydrazide (**5**)

Procedure A was followed with 100.4 mg (0.30 mmol) of **DAB-1** and 36 μL (0.31 mmol) of *p*-tolualdehyde, which yielded 86.2 mg (85%) of the desired material **5**. IR (ν, cm^−1^): 3503 (N-H), 3170–3033 (Csp^2^-H), 2921–2861 (Csp^3^-H), 1716 (C=O), 1650 (C=O), 1612 (C=C); ^1^H NMR (200 MHz, DMSO-*d*_6_, δ ppm): 11.85 (s, 1H), 8.42 (s, 1H), 8.01 (d, *J* = 8.5 Hz, 2H), 7.64 (d, *J* = 8.0 Hz, 2H), 7.52 (d, *J* = 8.5 Hz, 2H), 7.37–7.09 (m, 4H), 2.35 (s, 3H); ^13^C NMR (50 MHz, DMSO-*d*_6_, δ ppm): 169.64, 162.40, 148.04, 139.98, 134.85, 134.43, 132.44, 131.56, 129.47, 128.23, 127.11, 126.26, 21.05; ESI+ HRMS: (M+H)+ calculated for C_19_H_16_N_3_O_3_ = 334.1186; found = 334.1181.

##### Synthesis of 4-(2,5-Dioxo-2,5-Dihydro-1*H*-Pyrrol-1-yl)-*N*-(2-Hydroxybenzylidene)Benzohydrazide (**6**)

Procedure A was followed with 101.1 mg (0.31 mmol) of **DAB-1** and 100 μL (0.94 mmol) of salicylaldehyde, which yielded 78.5 mg (78%) of the desired material **6**. IR (ν, cm^−1^): 3601 (O-H), 3471 (N-H), 3184–3059 (Csp^2^-H), 1708 (C=O), 1655 (C=O), 1617 (C=C), 1157 (C-O); ^1^H NMR (200 MHz, DMSO-*d*_6_, δ ppm): 12.16 (s, 1H), 11.24 (s, 1H), 8.66 (s, 1H), 8.04 (d, *J* = 8.5 Hz, 2H), 7.58–7.59 (m, 3H), 7.41–7.20 (m, 3H), 6.95 (d, *J* = 7.8 Hz, 2H); ^13^C NMR (50 MHz, DMSO-*d*_6_, δ ppm): 169.62, 162.18, 157.46, 148.36, 134.87, 134.68, 131.74, 131.47, 129.45, 128.29, 126.30, 119.37, 118.69, 116.42; ESI+ HRMS: (M+H)+ calculated for C_18_H_14_N_3_O_4_ = 336.0979; found = 336.0973.

##### Synthesis of 4-(2,5-Dioxo-2,5-Dihydro-1*H*-Pyrrol-1-yl)-*N*-(3-Hydroxybenzylidene)Benzohydrazide (**7**)

Procedure A was followed with 101.5 mg (0.31 mmol) of **DAB-1** and 37.4 mg (0.31 mmol) of 3-hydroxybenzaldehyde, which yielded 90.6 mg (88%) of the desired material **7**. IR (ν, cm^−1^): 3467 (N-H), 3271 (O-H), 3093 (Csp^2^-H), 1709 (C=O), 1641 (C=O), 1614 (C=C), 1146 (C-O); ^1^H NMR (200 MHz, DMSO-*d*_6_, δ ppm): 11.86 (s, 1H), 9.64 (s, 1H), 8.37 (s, 1H), 8.01 (d, *J* = 8.0 Hz, 2H), 7.52 (d, *J* = 8.1 Hz, 2H), 7.30–7.01 (m, 5H), 6.84 (d, *J* = 7.0 Hz, 1H); ^13^C NMR (50 MHz, DMSO-*d*_6_, δ ppm): 169.67, 162.51, 157.70, 148.14, 135.55, 134.88, 134.51, 132.40, 129.95, 128.28, 126.31, 118.91, 117.55, 112.67; ESI+ HRMS: (M+H)+ calculated for C_18_H_14_N_3_O_4_ = 336.0979; found = 336.0978.

##### Synthesis of 4-(2,5-Dioxo-2,5-Dihydro-1*H*-Pyrrol-1-yl)-*N*-(4-Hydroxybenzylidene)Benzohydrazide (**8**)

Procedure A was followed with 101.3 mg (0.30 mmol) of **DAB-1** and 37.0 mg (0.30 mmol) of 4-hydroxybenzaldehyde, which quantitatively yielded the desired material **8**. IR (ν, cm^−1^): 3487 (N-H), 3396 (O-H), 3172–3022 (Csp^2^-H), 1707 (C=O), 1648 (C=O), 1609 (C=C), 1142 (C-O); ^1^H NMR (200 MHz, DMSO-*d*_6_): 11.72 (s, 1H), 9.95 (s, 1H), 8.36 (s, 1H), 8.00 (d, *J* = 8.4 Hz, 2H), 7.60–7.46 (m, 4H), 7.23 (s, 2H, maleimide), 6.85 (d, *J* = 8.4 Hz, 2H); ^13^C NMR (50 MHz, DMSO-*d*_6_, δ ppm): 169.65, 162.21, 159.48, 148.34, 134.85, 134.32, 132.61, 128.90, 128.16, 126.25, 125.24, 115.73; ESI+ HRMS: (M+H)+ calculated for C_18_H_14_N_3_O_4_ = 336.0979; found = 336.0978.

##### Synthesis of 4-(2,5-Dioxo-2,5-Dihydro-1*H*-Pyrrol-1-yl)-*N*-(2-Methoxybenzylidene)Benzohydrazide (**9**)

Procedure A was followed with 100.5 mg (0.30 mmol) of **DAB-1** and 42.8 mg (0.31 mmol) of 2-methoxybenzaldehyde, which yielded 78.7 mg (74%) of the desired material **9**. IR (ν, cm^−1^): 3464 (N-H), 3116–3024 (Csp^2^-H), 2987–2849 (Csp^3^-H), 1755 (C=O), 1705 (C=O), 1599 (C=C), 1155 (C-O); ^1^H NMR (200 MHz, DMSO-*d*_6_, δ ppm): 11.90 (s, 1H), 8.82 (s, 1H), 8.03 (d, *J* = 8.5 Hz, 2H), 7.89 (d, *J* = 6.4 Hz, 1H), 7.55–7.38 (m, 3H), 7.23 (s, 2H, maleimide), 7.18–6.93 (m, 2H), 3.87 (s, 3H); ^13^C NMR (50 MHz, DMSO-*d*_6_, δ ppm): 169.65, 162.25, 157.79, 143.43, 134.85, 134.45, 132.32, 131.64, 128.24, 126.23, 125.52, 122.26, 120.77, 111.87, 55.71 ESI+ HRMS: (M+H)+ calculated for C_19_H_16_N_3_O_4_ = 350.1135; found = 350.1132.

##### Synthesis of 4-(2,5-Dioxo-2,5-Dihydro-1*H*-Pyrrol-1-yl)-*N*-(3-Methoxybenzylidene)Benzohydrazide (**10**)

Procedure B was followed with 100.2 mg (0.30 mmol) of **DAB-1** and 41.3 mg (0.30 mmol) of 3-methoxybenzaldehyde, which yielded 90.5 mg (86%) of the desired material **10**. IR (ν, cm^−1^): 3476 (N-H), 3183–3073 (Csp^2^-H), 2999–2833 (Csp^3^-H), 1736 (C=O), 1713 (C=O), 1647 (C=C), 1172 (C-O); ^1^H NMR (200 MHz, DMSO-*d*_6_, δ ppm): 11.92 (s, 1H), 8.44 (s, 1H), 8.01 (d, *J* = 8.4 Hz, 2H), 7.66–7.14 (m, 7H), 7.03 (d, *J* = 8.0 Hz, 1H), 3.82 (s, 3H); ^13^C NMR (50 MHz, DMSO-*d*_6_, δ ppm): 169.63, 159.55, 147.90, 135.69, 134.86, 134.50, 132.34, 129.97, 128.35, 128.27, 126.28, 120.06, 116.27, 111.25, 55.18; ESI+ HRMS: (M+H)+ calculated for C_19_H_16_N_3_O_4_ = 350.1135; found = 350.1128.

##### Synthesis of 4-(2,5-Dioxo-2,5-Dihydro-1*H*-Pyrrol-1-yl)-*N*-(2-Nitrobenzylidene)Benzohydrazide (**11**)

Procedure A was followed with 100.1 mg (0.30 mmol) of **DAB-1** and 46.9 mg (0.31 mmol) of 2-nitrobenzaldehyde, which yielded 73.7 mg (68%) of the desired material **11**. IR (ν, cm^−1^): 3465 (N-H), 3193–3050 (Csp^2^-H), 1699 (C=O), 1648 (C=O), 1584 (C=C); ^1^H NMR (200 MHz, DMSO-*d*_6_, δ ppm): 12.27 (s, 1H), 8.88 (s, 1H), 8.20–8.01 (m, 4H), 7.84 (t, *J* = 7.3 Hz, 1H), 7.73–7.62 (m, 1H), 7.54 (d, *J* = 8.3 Hz, 2H), 7.24 (s, 2H, maleimide); ^13^C NMR (50 MHz, DMSO-*d*_6_, δ ppm): 169.61, 162.69, 148.25, 143.16, 134.86, 134.72, 133.75, 131.88, 130.72, 128.67, 128.39, 127.96, 126.23, 124.68; ESI+ HRMS: (M+H)+ calculated for C_18_H_13_N_4_O_5_ = 365.0880; found = 365.0874.

##### Synthesis of 4-(2,5-Dioxo-2,5-Dihydro-1*H*-Pyrrol-1-yl)-*N*-(3-Nitrobenzylidene)Benzohydrazide (**12**)

Procedure A was followed with 100.5 mg (0.30 mmol) of **DAB-1** and 46.7 mg (0.31 mmol) of 3-nitrobenzaldehyde, which yielded 95.0 mg (86%) of the desired material **12**. IR (ν, cm^−1^): 3382 (N-H), 3082–3042 (Csp^2^-H), 1709 (C=O), 1658 (C=O), 1621 (C=C); ^1^H NMR (200 MHz, DMSO-*d*_6_, δ ppm): 12.19 (s, 1H), 8.58 (s, 2H), 8.23 (dd, *J* = 19.7, 7.7 Hz, 2H), 8.04 (d, *J* = 7.9 Hz, 2H), 7.77 (t, *J* = 7.9 Hz, 1H), 7.55 (d, *J* = 7.2 Hz, 2H), 7.24 (s, 2H, maleimide); ^13^C NMR (50 MHz, DMSO-*d*_6_, δ ppm): 169.62, 162.76, 148.25, 145.54, 136.15, 134.87, 134.68, 133.41, 132.05, 130.50, 128.38, 126.27, 124.31, 120.97; ESI+ HRMS: (M+H)+ calculated for C_18_H_13_N_4_O_5_ = 365.0880; found = 365.0873.

##### Synthesis of 4-(2,5-Dioxo-2,5-Dihydro-1*H*-Pyrrol-1-yl)-*N*-(4-Bromobenzylidene)Benzohydrazide (**13**)

Procedure A was followed with 100.3 mg (0.30 mmol) of **DAB-1** and 56.3 mg (0.30 mmol) of 4-bromobenzaldehyde, which yielded 94.5 mg (78%) of the desired material **13**. IR (ν, cm^−1^): 3383 (N-H), 3167–3011 (Csp^2^-H), 1714 (C=O), 1650 (C=O), 1614 (C=C); ^1^H NMR (200 MHz, DMSO-*d*_6_, δ ppm): 11.99 (s, 1H), 8.44 (s, 1H), 8.02 (d, *J* = 7.7 Hz, 2H), 8.07–7.42 (m, 6H), 7.23 (s, 2H, maleimide); ^13^C NMR (50 MHz, DMSO-*d*_6_, δ ppm): 169.62, 162.54, 146.73, 134.85, 134.55, 133.56, 132.23, 131.87, 128.98, 128.29, 126.26, 123.36; ESI+ HRMS: (M+H)+ calculated for C_18_H_13_BrN_3_O_3_ = 398.0135; found = 398.0126.

##### Synthesis of 4-(2,5-Dioxo-2,5-Dihydro-1*H*-Pyrrol-1-yl)-*N*-(2-Hydroxy-3-Methoxybenzylidene)Benzohydrazide (**14**)

Procedure A was followed with 300.2 mg (0.91 mmol) of **DAB-1** and 138.5 mg (0.91 mmol) of *o*-vanillin, which yielded 300.6 mg (91%) of the desired material **14**. IR (ν, cm^−1^): 3504 (O-H), 3451 (N-H), 3103 (Csp^2^-H), 2942 (Csp^3^-H), 1700 (C=O), 1654 (C=O), 1610 (C=C), 1133 (C-O); ^1^H NMR (200 MHz, DMSO-*d*_6_, δ ppm): 12.13 (s, 1H), 10.92 (s, 1H), 8.67 (s, 1H), 8.04 (d, *J* = 8.5 Hz, 2H), 7.54 (d, *J* = 8.5 Hz, 2H), 7.33–7.12 (m, 3H), 7.05 (d, *J* = 7.5 Hz, 1H), 6.87 (t, *J* = 7.9 Hz, 1H), 3.82 (s, 3H); ^13^C NMR (50 MHz, DMSO-*d*_6_, δ ppm): 169.63, 162.18, 148.26, 147.96, 147.17, 134.88, 134.67, 131.80, 128.30, 126.31, 120.74, 119.08, 118.95, 113.84, 55.83; ESI+ HRMS: (M+H)+ calculated for C_19_H_16_N_3_O_5_ = 366.1084; found = 366.1075.

##### Synthesis of 4-(2,5-Dioxo-2,5-Dihydro-1*H*-Pyrrol-1-yl)-*N*-(3-Hydroxy-4-Methoxybenzelidene)Benzohydrazide (**15**)

Procedure B was followed with 101.9 mg (0.31 mmol) of **DAB-1** and 46.8 mg (0.31 mmol) of isovanillin, which yielded 39.7 mg (35%) of the desired material **15**. IR (ν, cm^−1^): 3471 (N-H), 3330 (O-H), 3096 (Csp^2^-H), 2843 (Csp^3^-H), 1711 (C=O), 1643 (C=O), 1603 (C=C), 1141 (C-O); ^1^H NMR (200 MHz, DMSO-*d*_6_, δ ppm): 11.73 (s, 1H), 9.32 (s, 1H), 8.30 (s, 1H), 8.00 (d, *J* = 8.5 Hz, 2H), 7.51 (d, *J* = 8.6 Hz, 2H), 7.30–7.21 (m, 3H), 7.12–6.95 (m, 2H), 3.81 (s, 3H); ^13^C NMR (50 MHz, DMSO-*d*_6_, δ ppm): 169.65, 162.25, 149.85, 148.16, 146.88, 134.85, 134.35, 132.57, 128.17, 127.09, 126.26, 120.36, 112.29, 111.88, 55.58; ESI+ HRMS: (M+H)+ calculated for C_20_H_20_N_3_O_6_ = 398.1347; found = 398.1318.

##### Synthesis of 4-(2,5-Dioxo-2,5-Dihydro-1*H*-Pyrrol-1-yl)-*N*-(4-Hydroxy-3-Methoxybenzelidene)Benzohydrazide (**16**)

Procedure B was followed with 100.3 mg (0.30 mmol) of **DAB-1** and 46.1 mg (0.30 mmol) of vanillin, which yielded 110.6 mg (80%) of the desired material **16**. IR (ν, cm^−1^): 3395 (N-H), 3244 (O-H), 3097 (Csp^2^-H), 2958–2858 (Csp^3^-H), 1715 (C=O), 1642 (C=O), 1605 (C=C), 1133 (C-O); ^1^H NMR (200 MHz, DMSO-*d*_6_, δ ppm): 11.72 (s, 1H), 9.57 (s, 1H), 8.34 (s, 1H), 8.00 (d, *J* = 8.3 Hz, 2H), 7.51 (d, *J* = 8.3 Hz, 2H), 7.33 (s, 1H), 7.23 (s, 2H, maleimide), 7.10 (d, *J* = 7.1 Hz, 1H), 6.85 (d, *J* = 8.5 Hz, 1H), 3.84 (s, 3H); ^13^C NMR (50 MHz, DMSO-*d*_6_, δ ppm): 169.68, 162.39, 150.29, 149.18, 149.08, 148.81, 148.61, 148.07, 134.87, 133.91, 132.64, 128.20, 126.31, 125.68, 55.59; ESI+ HRMS: (M+H)+ calculated for C_20_H_20_N_3_O_6_ = 398.1347; found = 398.1325.

##### Synthesis of 4-(2,5-Dioxo-2,5-Dihydro-1*H*-Pyrrol-1-yl)-*N*-(Furan-2-Ylmethylene)Benzohydrazide (**17**)

Procedure B was followed with 102.0 mg (0.31 mmol) of **DAB-1** and 50 µL (0.61 mmol) of furfural, which yielded 89.5 mg (94%) of the desired material **17**. IR (ν, cm^−1^): 3474 (N-H), 3272–3093 (Csp^2^-H), 1706 (C=O), 1654 (C=O), 1619 (C=C), 1150 (C-O); ^1^H NMR (200 MHz, DMSO-*d*_6_, δ ppm): 11.85 (s, 1H), 8.34 (s, 1H), 8.08–7.76 (m, 3H), 7.52 (d, *J* = 8.4 Hz, 2H), 7.23 (s, 2H, maleimide), 6.96 (s, 1H), 6.65 (s, 1H); ^13^C NMR (50 MHz, DMSO-*d*_6_, δ ppm): 169.63, 162.41, 149.38, 145.28, 137.72, 134.85, 134.49, 132.28, 128.22, 126.27, 113.70, 112.22; ESI+ HRMS: (M+H)+ calculated for C_16_H_12_N_3_O_4_ = 310.0822; found = 310.0820.

##### Synthesis of 4-(2,5-Dioxo-2,5-Dihydro-1*H*-Pyrrol-1-yl)-*N*-(Furan-3-Ylmethylene)Benzohydrazide (**18**)

Procedure A was followed with 100.3 mg (0.30 mmol) of **DAB-1** and 27 µL (0.31 mmol) of 3-furaldehyde, which yielded 24.5 mg (26%) of the desired material **18**. IR (ν, cm^−1^): 3465 (N-H), 3152–3065 (Csp^2^-H), 1709 (C=O), 1645 (C=O), 1622 (C=C), 1159 (C-O); ^1^H NMR (200 MHz, DMSO-*d*_6_, δ ppm): 11.76 (s, 1H), 8.41 (s, 1H), 8.18 (s, 1H), 7.98 (d, *J* = 8.4 Hz, 2H), 7.78 (s, 1H), 7.51 (d, *J* = 8.5 Hz, 2H), 7.23 (s, 2H, maleimide), 6.82 (s, 1H); ^13^C NMR (50 MHz, DMSO-*d*_6_, δ ppm): 169.64, 162.33, 145.52, 144.87, 140.91, 134.84, 134.40, 132.50, 128.19, 126.26, 122.60, 107.15; ESI+ HRMS: (M+H)+ calculated for C_16_H_12_N_3_O_4_ = 310.0822; found = 310.0816.

##### Synthesis of 4-(2,5-Dioxo-2,5-Dihydro-1*H*-Pyrrol-1-yl)-*N*-(5-Methylfuran-2-Ylmethylene)Benzohydrazide (**19**)

Procedure A was followed with 101.6 mg (0.31 mmol) of **DAB-1** and 31 µL (0.31 mmol) of 5-methylfurfural, which quantitatively yielded of the desired material **19**. IR (ν, cm^−1^): 3472 (N-H), 3099 (Csp^2^-H), 2922 (Csp^3^-H), 1706 (C=O), 1663 (C=O), 1623 (C=C), 1147 (C-O); ^1^H NMR (200 MHz, DMSO-*d*_6_, δ ppm): 11.77 (s, 1H), 8.24 (s, 1H), 7.99 (d, *J* = 8.5 Hz, 2H), 7.51 (d, *J* = 8.5 Hz, 2H), 7.23 (s, 2H, maleimide), 6.82 (s, 1H), 6.27 (s, 1H), 2.36 (s, 3H); ^13^C NMR (50 MHz, DMSO-*d*_6_, δ ppm): 169.62, 162.30, 154.66, 147.84, 137.59, 134.84, 134.42, 132.37, 128.18, 126.25, 115.54, 108.60, 13.51; ESI+ HRMS: (M+H)+ calculated for C_17_H_14_N_3_O_4_ = 324.0979; found = 324.0971.

##### Synthesis of 4-(2,5-Dioxo-2,5-Dihydro-1*H*-Pyrrol-1-yl)-*N*-(5-Nitrofuran-2-Ylmethylene)Benzohydrazide (**20**)

Procedure A was followed with 102.1 mg (0.31 mmol) of **DAB-1** and 44.7 mg (0.32 mmol) of 5-nitrofurfural, which yielded 89.4 mg (82%) of the desired material **20**. IR (ν, cm^−1^): 3472 (N-H), 3113 (Csp^2^-H), 1719 (C=O), 1685 (C=O), 1654 (C=C), 1140 (C-O); ^1^H NMR (200 MHz, DMSO-*d*_6_, δ ppm): 12.29 (s, 1H), 8.41 (s, 1H), 8.02 (d, *J* = 8.5 Hz, 2H), 7.81 (d, *J* = 3.9 Hz, 1H), 7.55 (d, *J* = 8.5 Hz, 2H), 7.35–7.16 (m, 3H); ^13^C NMR (50 MHz, DMSO-*d*_6_, δ ppm): 169.58, 162.80, 151.94, 151.66, 135.69, 135.67, 134.87, 131.68, 128.45, 126.28, 115.46, 114.64; ESI+ HRMS: (M+H)+ calculated for C_16_H_11_N_4_O_6_ = 355.0673; found = 355.0667.

##### Synthesis of 4-(2,5-Dioxo-2,5-Dihydro-1*H*-Pyrrol-1-yl)-*N*-(5-(Hydroxymethyl)Furan-2-Ylmethylene)Benzohydrazide (**21**)

Procedure B was followed with 100.5 mg (0.30 mmol) of **DAB-1** and 31 µL (0.31 mmol) of 5-(hydroxymethyl)furfural, which yielded 50.9 mg (50%) of the desired material **21**. IR (ν, cm^−1^): 3399 (N-H), 3233 (O-H), 3099 (Csp^2^-H), 2931–2867 (Csp^3^-H), 1710 (C=O), 1655 (C=O), 1608 (C=C), 1141 (C-O); ^1^H NMR (200 MHz, DMSO-*d*_6_, δ ppm): 11.82 (s, 1H), 8.28 (s, 1H), 7.99 (d, *J* = 8.3 Hz, 2H), 7.52 (d, *J* = 8.4 Hz, 2H), 7.23 (s, 2H, maleimide), 6.87 (s, 1H), 6.45 (s, 1H), 4.46 (s, 2H); ^13^C NMR (50 MHz, DMSO-*d*_6_, δ ppm): 169.64, 162.38, 157.96, 148.56, 137.60, 134.86, 134.48, 132.31, 128.23, 126.27, 114.92, 109.24, 55.74; ESI+ HRMS: (M+H)+ calculated for C_17_H_14_N_3_O_5_ = 340.0928; found = 340.0924.

##### Synthesis of 4-(2,5-Dioxo-2,5-Dihydro-1*H*-Pyrrol-1-yl)-*N*-(5-(4-Nitrophenyl)Furan-2-Ylmethylene)Benzohydrazide (**22**)

Procedure B was followed with 100.7 mg (0.30 mmol) of **DAB-1** and 67.8 mg (0.31 mmol) of 5-(4-nitrophenyl)-2-furaldehyde, which yielded 99.0 mg (76%) of the desired material **22**. IR (ν, cm^−1^): 3467 (N-H), 3105–3011 (Csp^2^-H), 2931–2867, 1717 (C=O), 1664 (C=O), 1610 (C=C), 1145 (C-O); ^1^H NMR (200 MHz, DMSO-*d*_6_, δ ppm): 12.01 (s, 1H), 8.43 (s, 1H), 8.32 (d, *J* = 9.0 Hz, 2H), 8.09–7.96 (m, 4H), 7.59–7.47 (m, 3H), 7.26–7.14 (m, 3H); ^13^C NMR (50 MHz, DMSO-*d*_6_, δ ppm): 169.61, 162.53, 152.50, 150.93, 146.33, 137.17, 135.17, 134.86, 134.60, 132.17, 128.28, 126.28, 124.63, 124.54, 116.40, 112.47; ESI+ HRMS: (M+H)+ calculated for C_22_H_15_N_4_O_6_ = 431.0986; found = 431.0981.

##### Synthesis of 4-(2,5-Dioxo-2,5-Dihydro-1*H*-Pyrrol-1-yl)-*N*-(Pyridin-3-Ylmethylene)Benzohydrazide (**23**)

Procedure A was followed with 105.6 mg (0.32 mmol) of **DAB-1** and 60 µL (0.64 mmol) of nicotinaldehyde, which yielded 100.7 mg (99%) of the desired material **23**. IR (ν, cm^−1^): 3470 (N-H), 3172–3024 (Csp^2^-H), 1712 (C=O), 1646 (C=O), 1608 (C=C); ^1^H NMR (200 MHz, DMSO-*d*_6_, δ ppm): 12.09 (s, 1H), 8.88 (s, 1H), 8.65–8.45 (m, 2H), 8.21–7.90 (m, 3H), 7.57–7.33 (m, 3H), 7.23 (s, 2H, maleimide); ^13^C NMR (50 MHz, DMSO-*d*_6_, δ ppm): 169.65, 162.66, 150.60, 148.62, 145.22, 134.88, 134.62, 133.74, 132.16, 130.29, 128.35, 126.32, 124.14; ESI+ HRMS: (M+H)+ calculated for C_17_H_13_N_4_O_3_ = 321.0982; found = 321.0979.

## 4. Conclusions

This study presents the synthesis of a series of 23 new hydrazide molecules and reports on their biological potential. Many of the compounds possess increased in vitro anti-inflammatory activity compared to the previous **DAB-1**. This work demonstrates that the hydrazinamide group of former DAB generations can be substituted for a hydrazide moiety to successfully enhance the biological potential of these compounds. Furthermore, the new derivatives typically display low toxicity, and this study uncovered that hydrazides possessing hydroxy/methoxy-substituted aromatic cores generally exhibit higher anti-inflammatory activity compared to their congeners. Of these compounds, methoxyphenol **14** bears the most pronounced activity against RAW 264.7 cells, although it displays similar activity levels as **DAB-1** in CD-1 mice. Further in vivo studies on the structure-activity relationship of DABs revealed that **14** exhibit greater efficiency than **DAB-1** in significantly inhibiting the growth of subcutaneous, highly invasive MB49-I tumors.

## Figures and Tables

**Table 1:** Structure of hydrazide derivatives **1–23**.

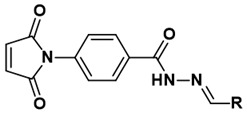					
Compound	R	Yield (%)	Compound	R	Yield (%)
**1**	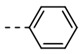	62	**13**	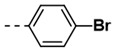	78
**2**	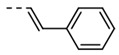	76	**14**	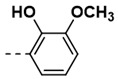	91
**3**	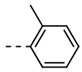	57	**15**	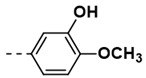	35
**4**	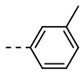	90	**16**	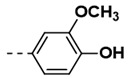	80
**5**	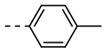	85	**17**	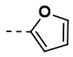	94
**6**	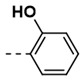	78	**18**	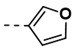	26
**7**	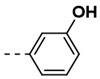	88	**19**	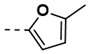	100
**8**	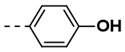	100	**20**	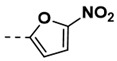	82
**9**	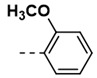	74	**21**	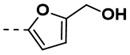	50
**10**	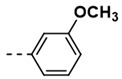	86	**22**	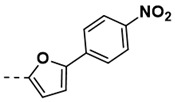	76
**11**	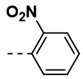	68	**23**	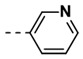	99
**12**	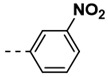	86			

**Table 2:** Relative viability of RAW 264.7 cells evaluated by an MTT assay after 30 min pretreatment with hydrazide derivatives **1–23** at 15 µM.

Compound	% Viability	Compound	% Viability
**DAB-1**	99 ± 2	**13**	91 ± 3
**1**	46 ± 2	**14**	98 ± 1
**2**	42 ± 2	**15**	110 ± 8
**3**	107 ± 4	**16**	110 ± 7
**4**	88 ± 3	**17**	90 ± 7
**5**	102 ± 2	**18**	102 ± 2
**6**	40 ± 1	**19**	111 ± 4
**7**	101 ± 2	**20**	112 ± 3
**8**	105 ± 3	**21**	93 ± 3
**9**	100 ± 2	**22**	102 ± 2
**10**	72 ± 1	**23**	106 ± 7
**11**	96 ± 2		
**12**	105 ± 4		

**Table 3:** Anti-inflammatory activities of hydrazide derivatives **1–23** against RAW 264.7 cells after 30 min pretreatment at 15 µM, evaluated by a Griess assay.

Compound	NO Concentration	Normalized NO	Normalized NO
	(% of Control)	Production (%)	Inhibition (%)
**DAB-1**	80 ± 2	81	19
**1**	19 ± 1	41	59
**2**	19 ± 2	45	55
**3**	100 ± 4	93	7
**4**	54 ± 3	61	39
**5**	62 ± 1	61	39
**6**	31 ± 2	78	22
**7**	56 ± 1	55	45
**8**	89 ± 6	85	15
**9**	80 ± 3	80	20
**10**	46 ± 4	64	36
**11**	62 ± 2	65	35
**12**	92 ± 1	97	3
**13**	94 ± 2	103	−3
**14**	<5%	<5%	>95%
**15**	96 ± 6	87	13
**16**	78 ± 1	71	29
**17**	91 ± 4	101	−1
**18**	23 ± 2	25	75
**19**	93 ± 3	84	16
**20**	84 ± 1	75	25
**21**	98 ± 3	105	-5
**22**	53 ± 4	52	48
**23**	50 ± 2	47	53

## Data Availability

The original contributions presented in this study are included in the article. Further inquiries can be directed to the corresponding author(s).

## Supplementary Materials

The following supporting information can be downloaded at: https://www.mdpi.com/article/10.3390/molecules30204035/s1, Figure S1: Effects of **DAB-1** and **14** on RAW 264.7 cells cell viability. Representative dose–response curves using MTT cell proliferation assay and IC_50_ calculation, using different doses of **DAB-1** or **14** (0, 5, 10, 20, 30 and 40 μM).

## Abbreviations

The following abbreviations are used in this manuscript:

BCa	Bladder cancer
PABA	*p*-aminobenzoic acid
DMSO	Dimethyl sulfoxide
IR	Infrared spectroscopy
NMR	Nuclear magnetic spectroscopy
TFA	Trifluoroacetic acid
NO	Nitric oxide
PBS	Phosphate-buffered saline
MTT	3-(4,5-Dimethylthiazol-2-yl)-2,5-Diphenyltetrazolium Bromide
IP	Intraperitoneal
