# Supplementary material for: Exploring the Synthesis, Anti-Inflammatory and Anti-Tumor Potential of 4-Maleimidylphenyl-Hydrazide Derivatives
Source: Molecules. 2025 Oct 10;30(20):4035. doi: 10.3390/molecules30204035 (PMC12565891; doi:10.3390/molecules30204035)

Supplemental Materials

Exploring the Synthesis, Anti-Inflammatory and Anti-Tumor Potential of 4-Maleimidylphenyl-Hydrazide Derivatives

Francis Cloutier [1], Alexis Paquin [1], Maude Cloutier [1], Yassine Oufqir [2], Laurie Fortin [2], Julie Girouard [2], Heidar-Ali Tajmir-Riahi [1], Carlos Reyes-Moreno [2,*] and Gervais Bérubé [1,*]

[1] Laboratoire de Recherche en Chimie Médicinale (LRCM) et Groupe de Recherche en Signalisation Cellulaire (GRSC), Département de Chimie, Biochimie et Physique, Université du Québec à Trois-Rivières, Trois-Rivières, QC G8Z 4M3, Canada;

[2] Laboratoire de Recherche en Oncologie et Immunobiologie (LROI) et Groupe de Recherche en Signalisation Cellulaire (GRSC), Département de biologie médicale, Université du Québec à Trois-Rivières, Trois-Rivières, QC G8Z 4M3, Canada

* Correspondance: carlos.reyes-moreno@uqtr.ca (C.R.-M.); gervais.berube@uqtr.ca (G.B.); Tel.: +1-819-376-5011 (ext. 3308) (C.R.-M.); +1-819-383-9334 (G.B.)

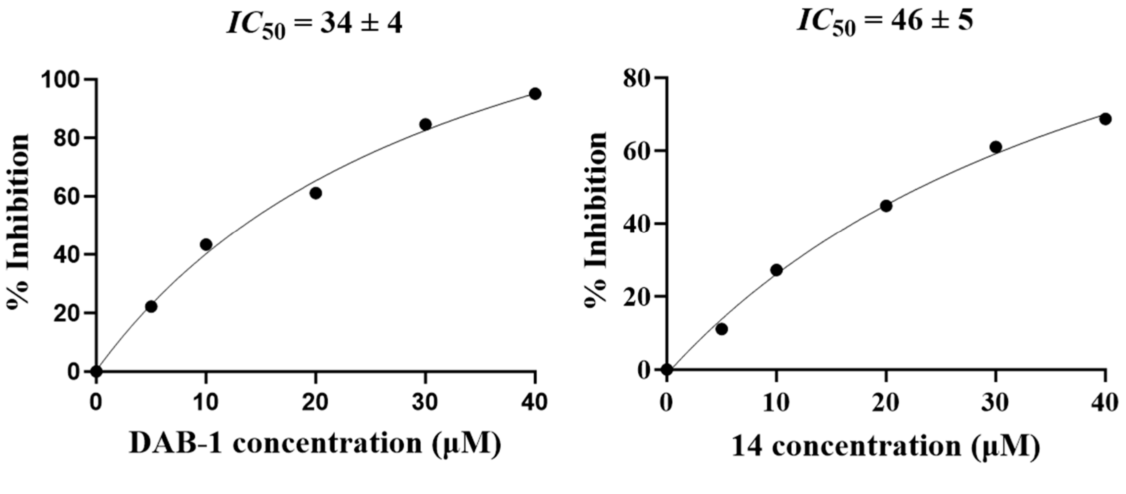

Figure S1. Effects of **DAB-1** and **14** on RAW 264.7 cells cell viability. Representative dose–response curves using MTT cell proliferation assay and IC_{50} calculation, using different doses of **DAB-1** or **14** (0, 5, 10, 20, 30 and 40 µM).

Proton and carbon NMR spectra of the new hydrazide derivatives.

^{1}H NMR: *N*-benzylidene-4-(2,5-dioxo-2,5-dihydro-1*H*-pyrrol-1-yl)benzohydrazide (**1**)

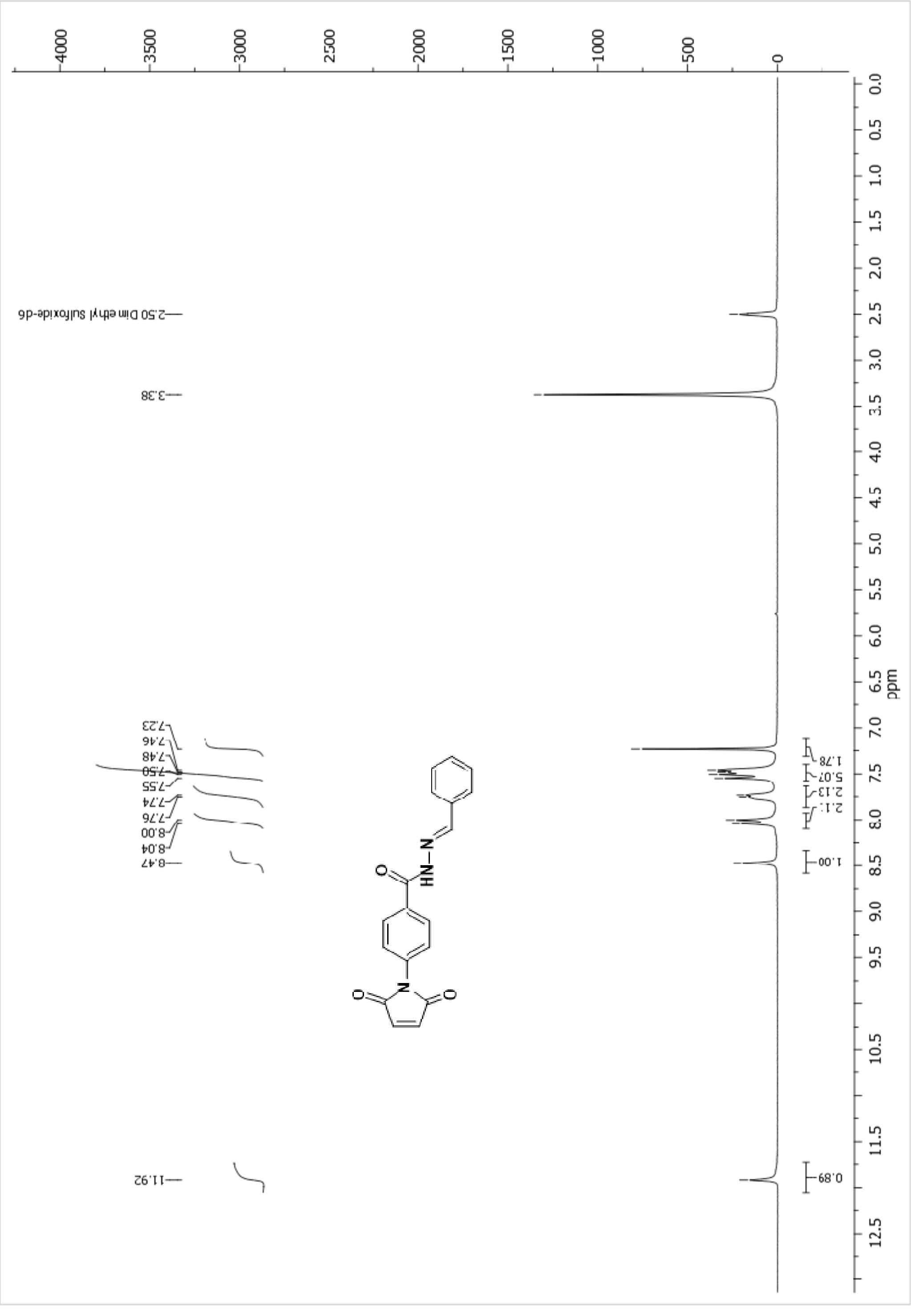

^{13}C NMR: *N*-benzylidene-4-(2,5-dioxo-2,5-dihydro-1*H*-pyrrol-1-yl)benzohydrazide (**1**)

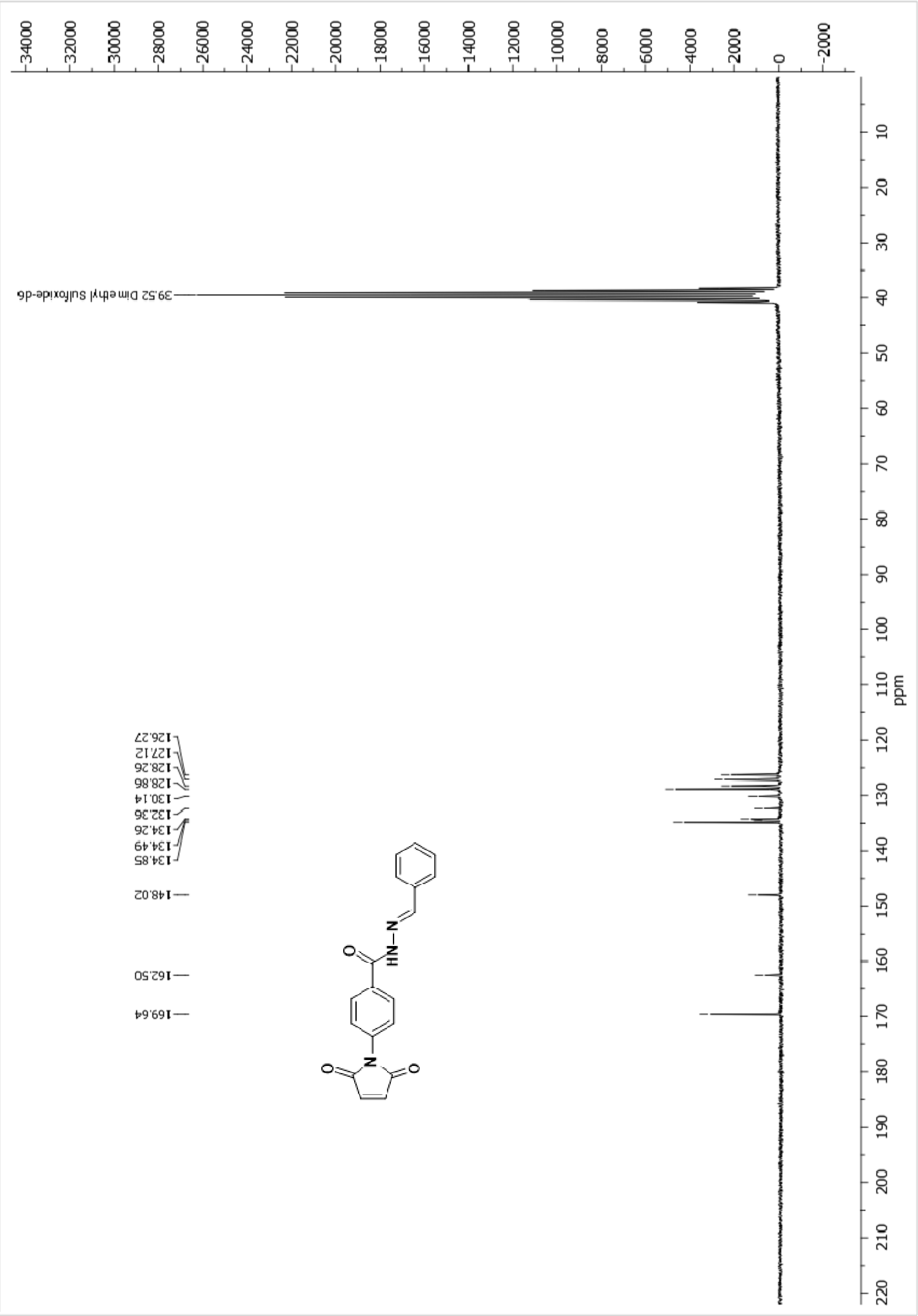

^{1}H NMR: 4-(2,5-dioxo-2,5-dihydro-1*H*-pyrrol-1-yl)-*N*-(3-phenyallylidene)benzohydrazide (**2**)

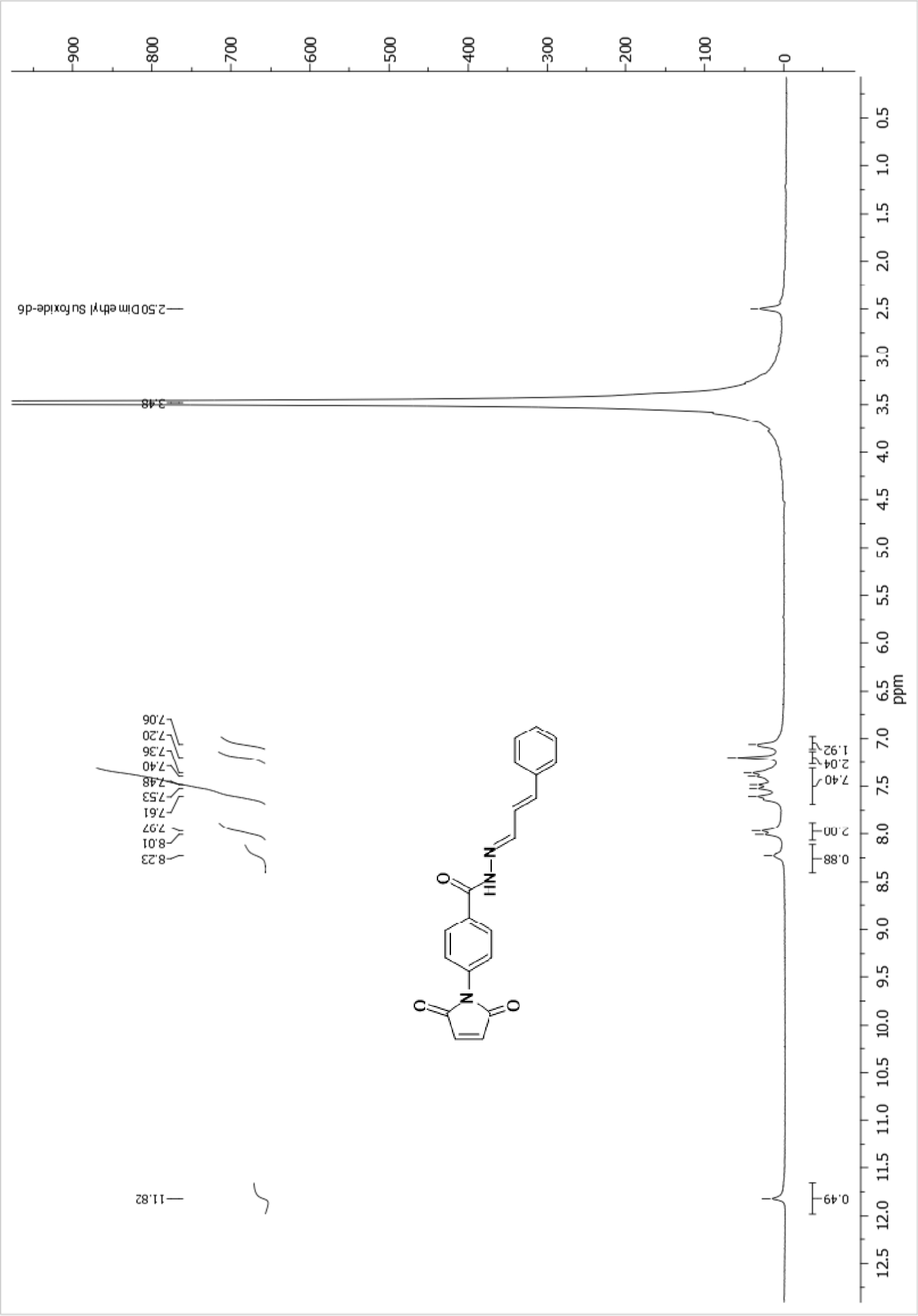

^{13}C NMR: 4-(2,5-dioxo-2,5-dihydro-1*H*-pyrrol-1-yl)-*N*-(3-phenyallylidene)benzohydrazide (**2**)

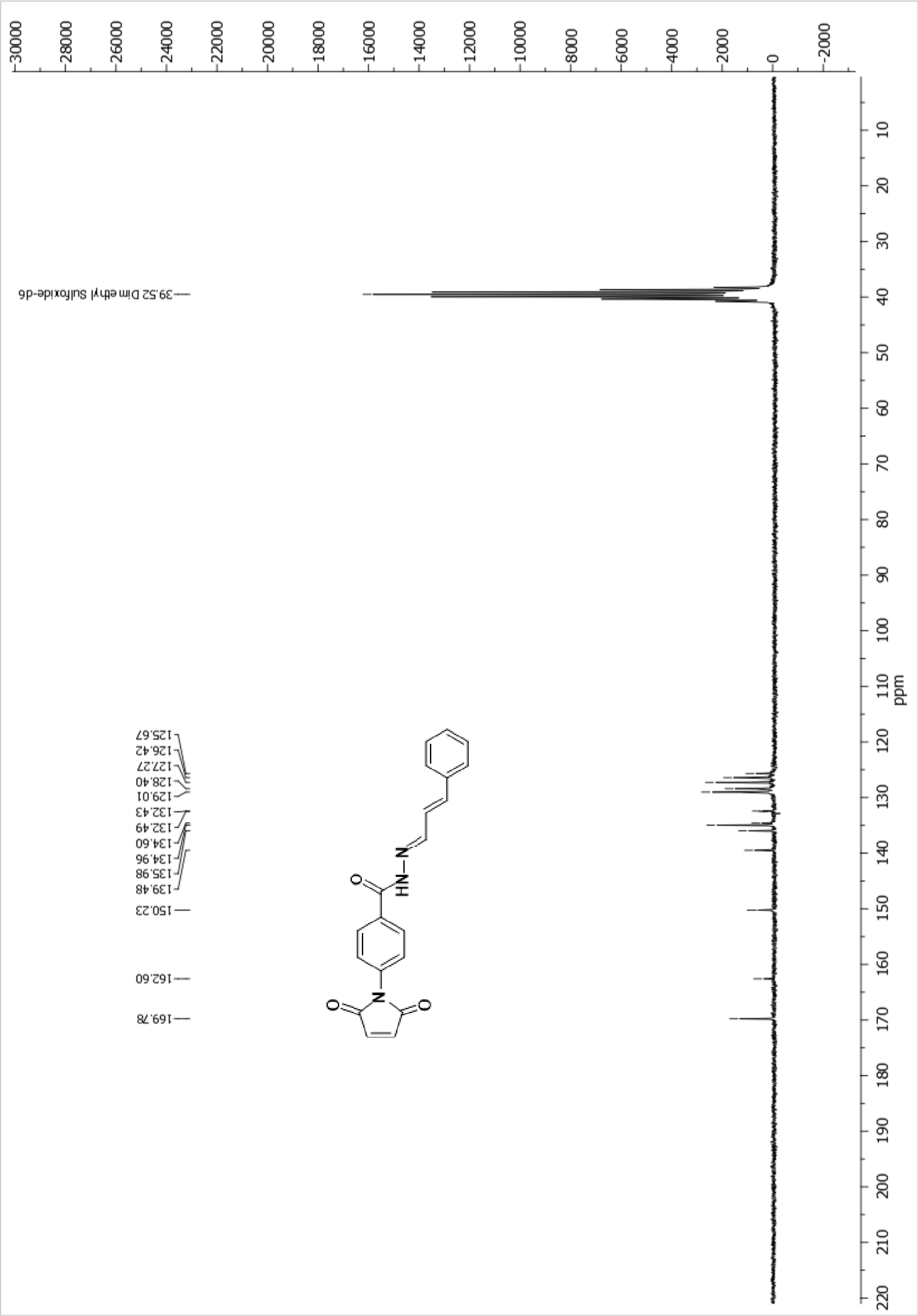

^{1}H NMR: 4-(2,5-dioxo-2,5-dihydro-1*H*-pyrrol-1-yl)-*N*-(2-methylbenzylidene)benzohydrazide (**3**)

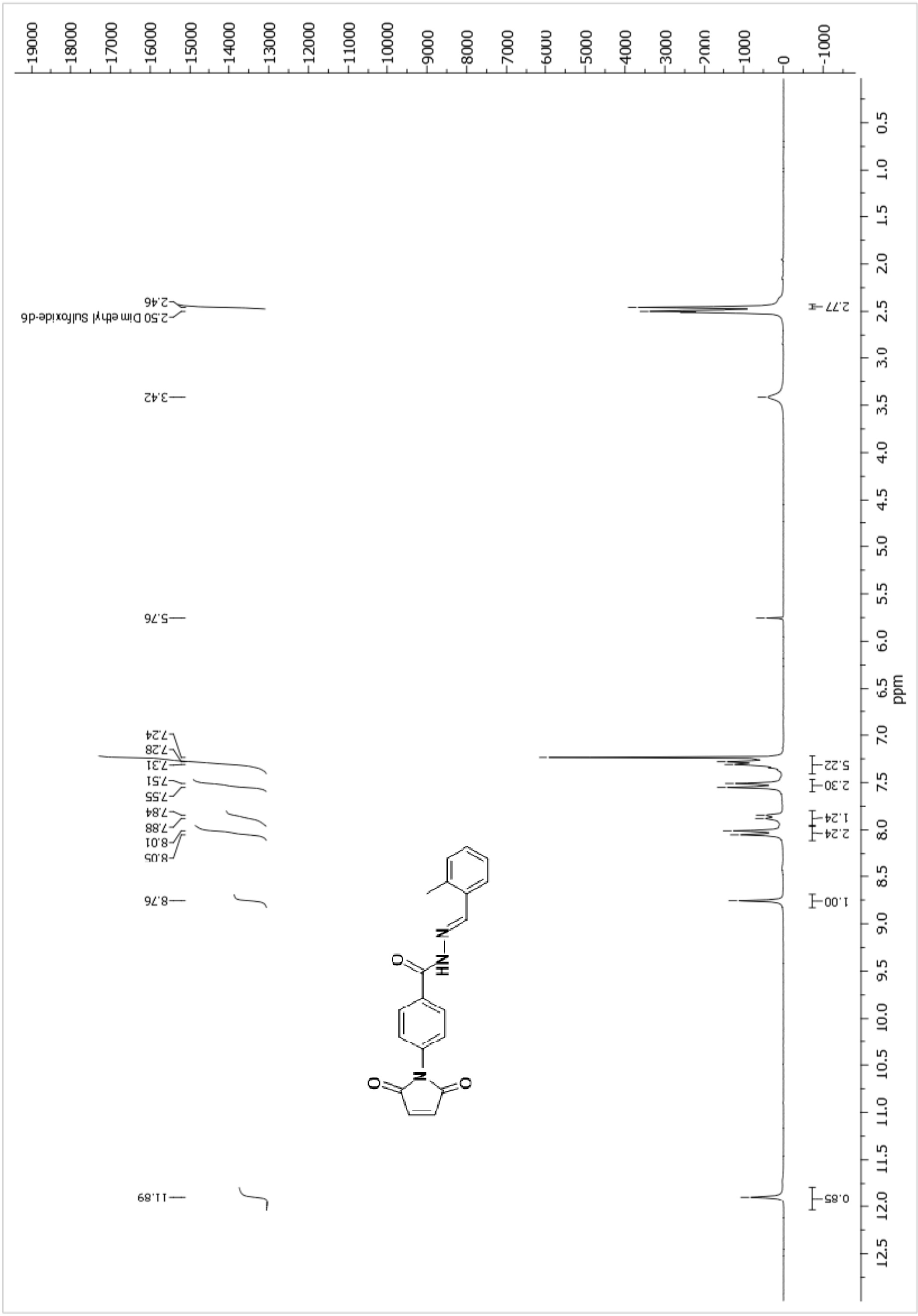

^{13}C NMR: 4-(2,5-dioxo-2,5-dihydro-1*H*-pyrrol-1-yl)-*N*-(2-methylbenzylidene)benzohydrazide (**3**)

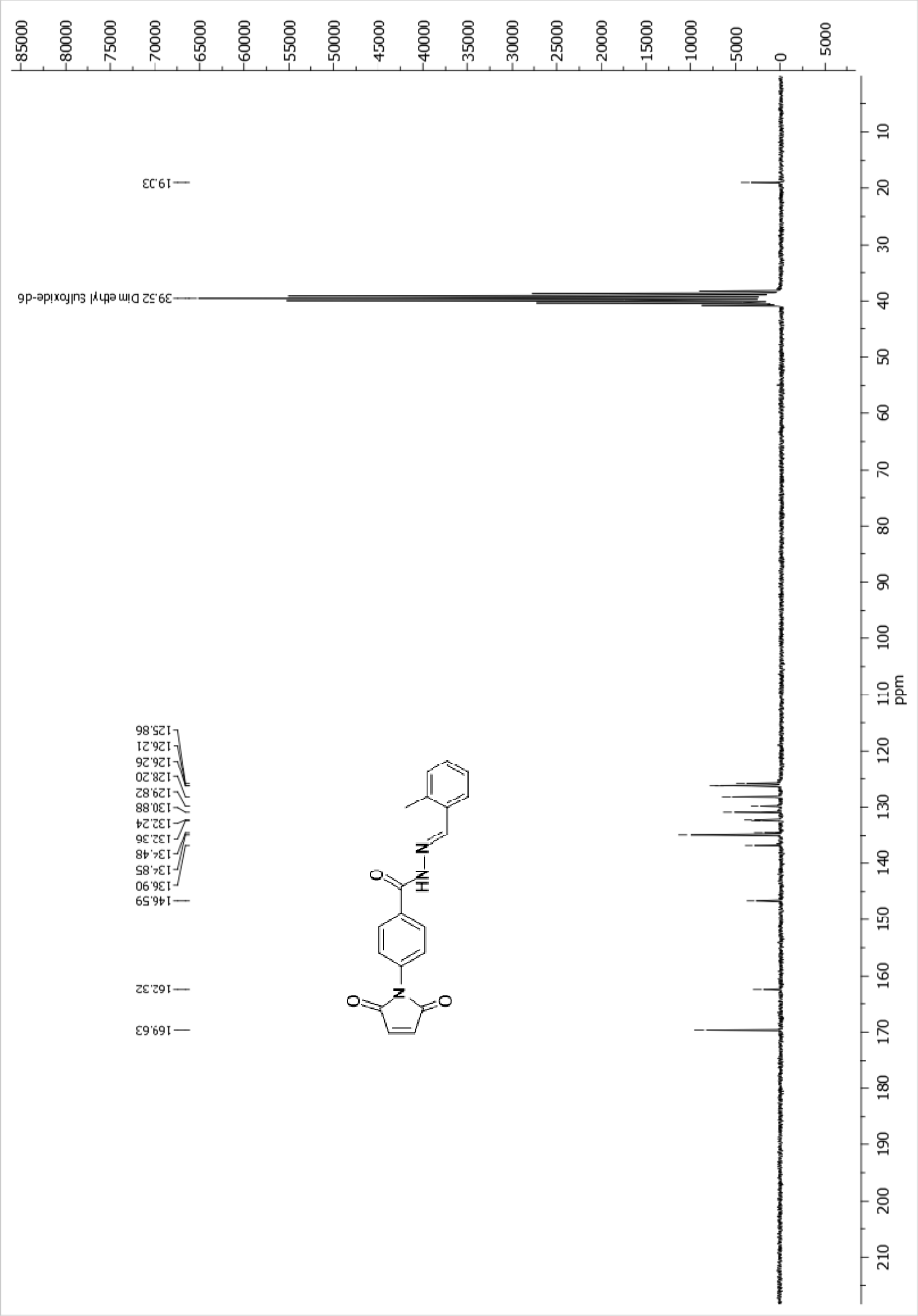

^{1}H NMR: 4-(2,5-dioxo-2,5-dihydro-1*H*-pyrrol-1-yl)-*N*-(3-methylbenzylidene)benzohydrazide (**4**)

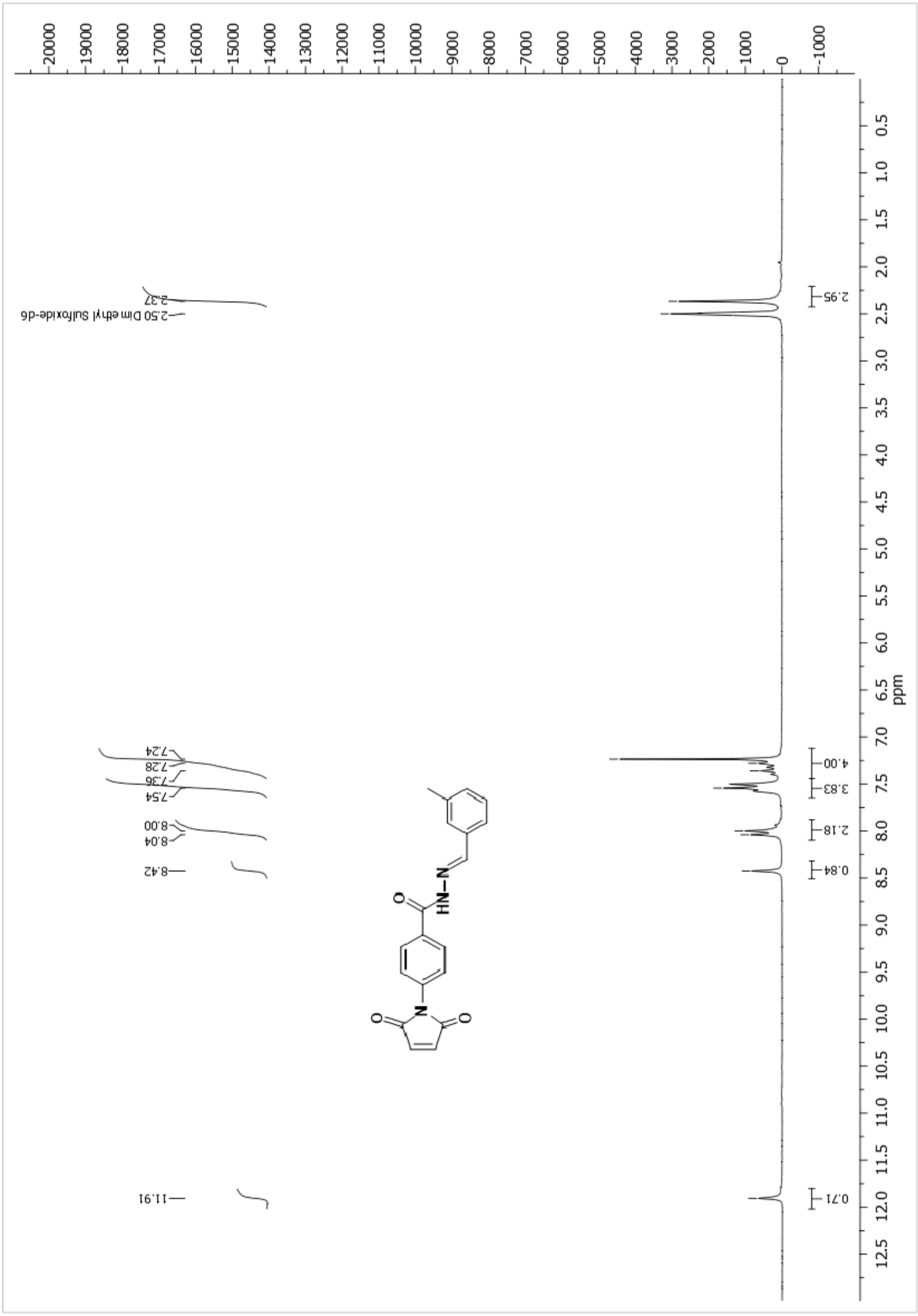

^{13}C NMR: 4-(2,5-dioxo-2,5-dihydro-1*H*-pyrrol-1-yl)-*N*-(3-methylbenzylidene)benzohydrazide (**4**)

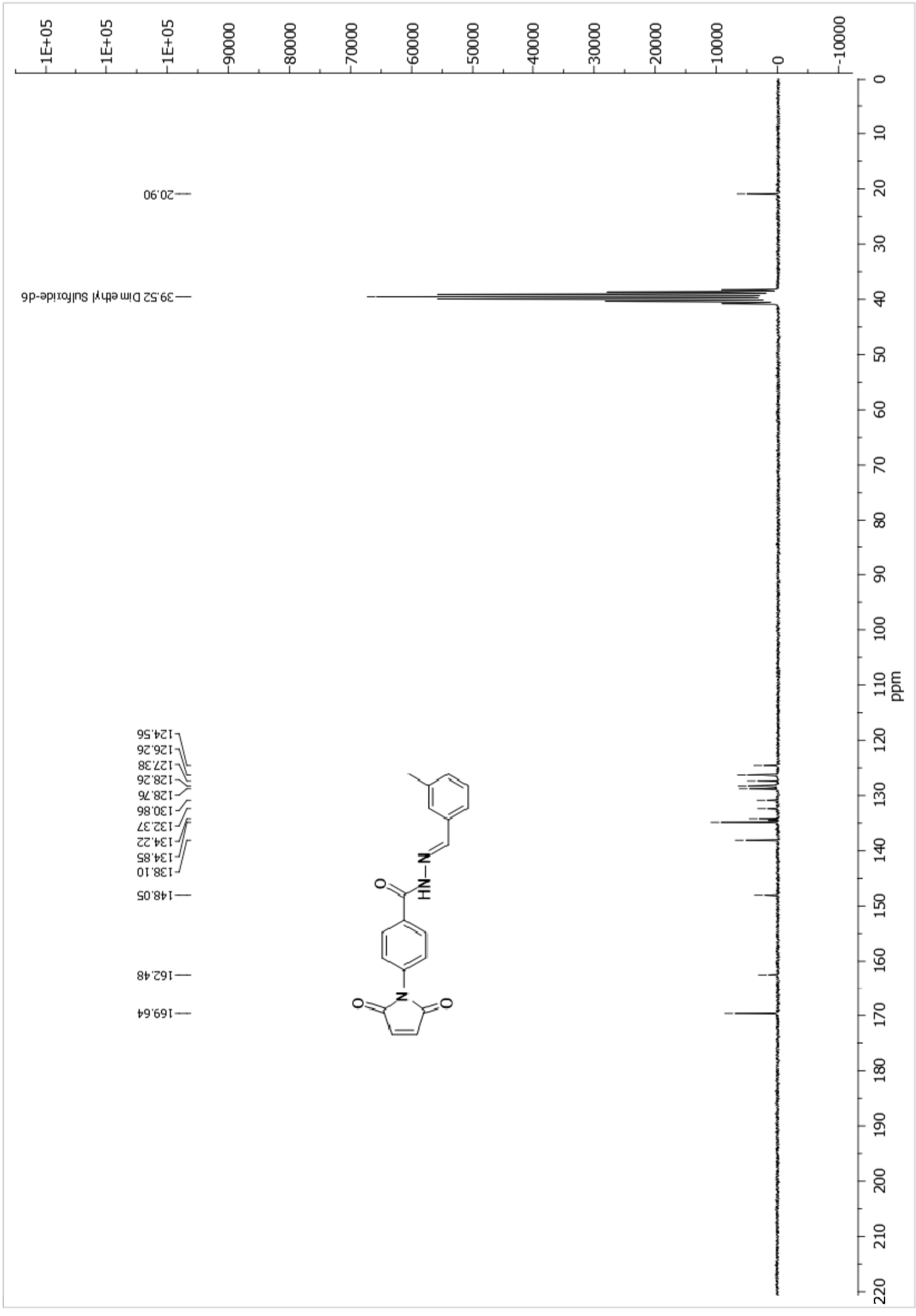

^{1}H NMR: 4-(2,5-dioxo-2,5-dihydro-1*H*-pyrrol-1-yl)-*N*-(4-methylbenzylidene)benzohydrazide (**5**)

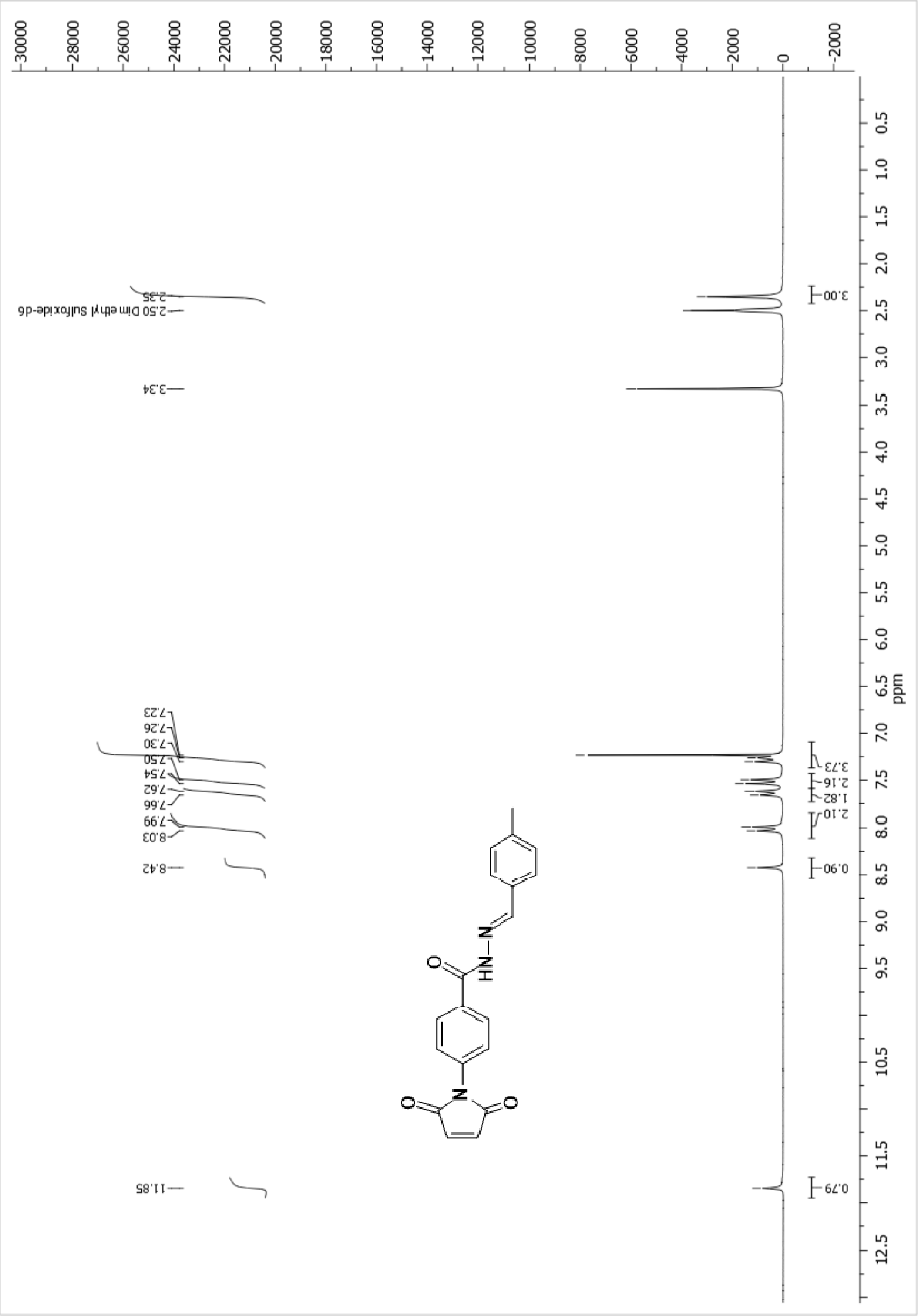

^{13}C NMR: 4-(2,5-dioxo-2,5-dihydro-1*H*-pyrrol-1-yl)-*N*-(4-methylbenzylidene)benzohydrazide (**5**)

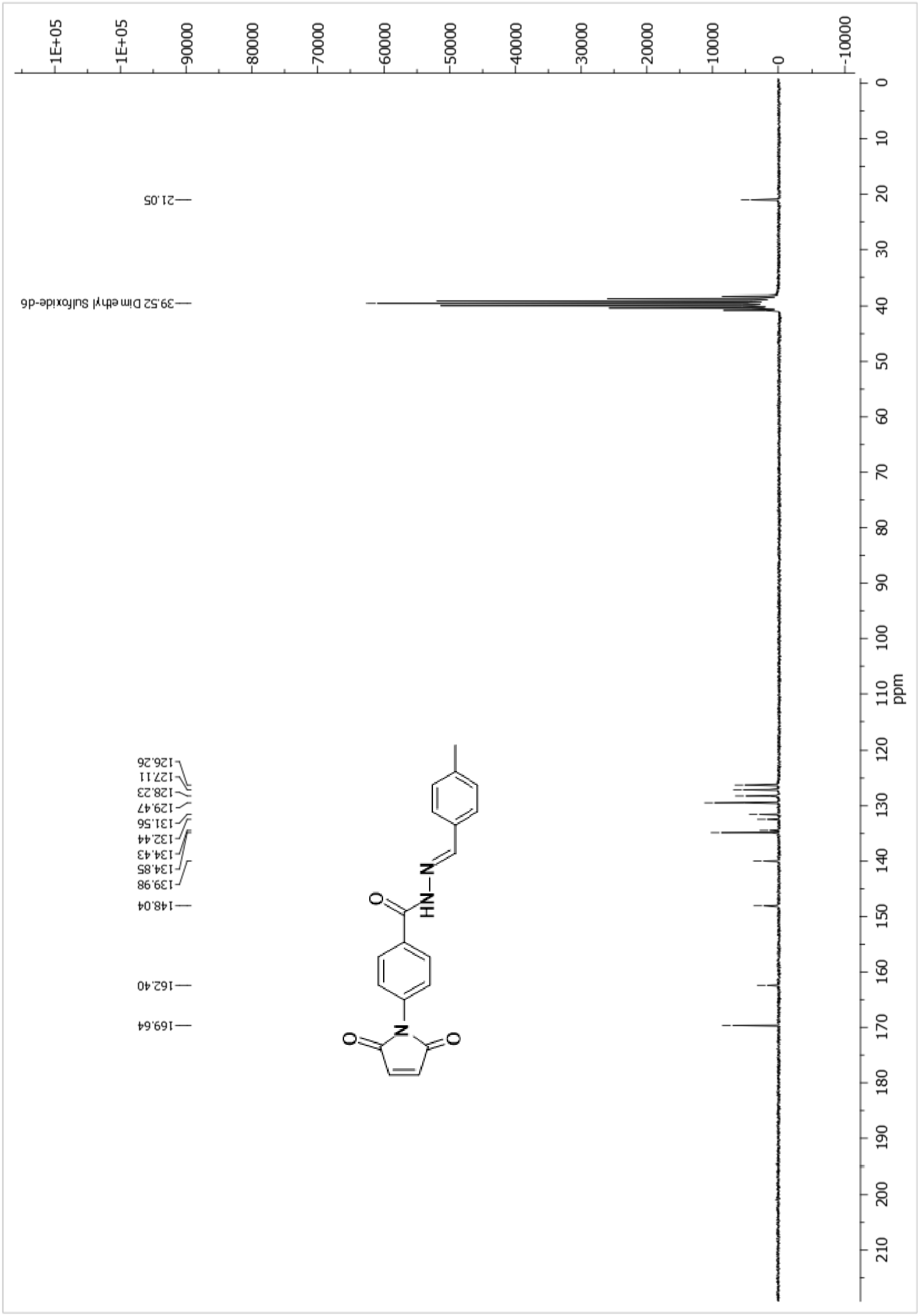

^{1}H NMR: 4-(2,5-dioxo-2,5-dihydro-1*H*-pyrrol-1-yl)-*N*-(2-hydroxybenzylidene)benzohydrazide (**6**)

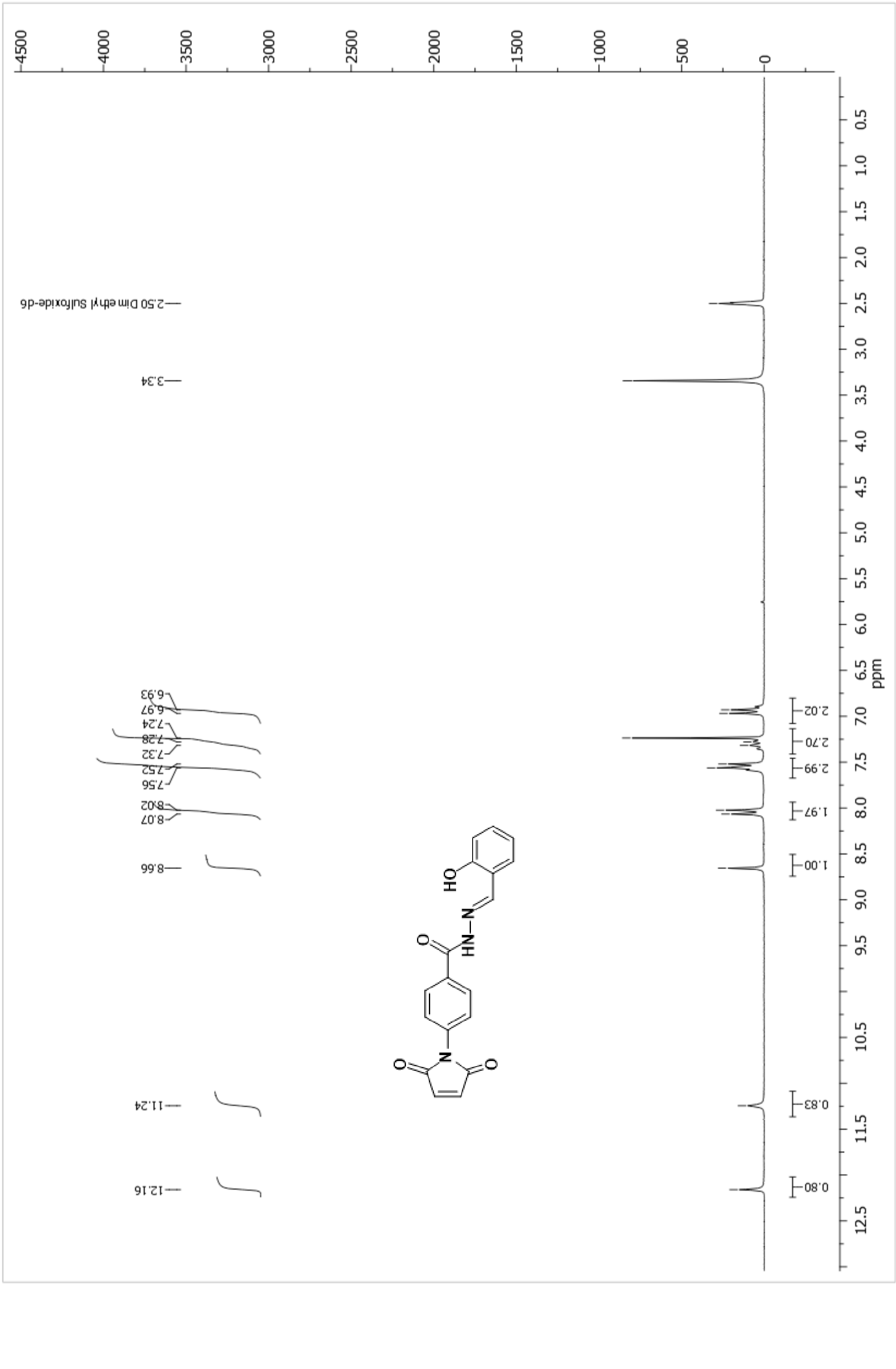

^{13}C NMR: 4-(2,5-dioxo-2,5-dihydro-1*H*-pyrrol-1-yl)-*N*-(2-hydroxybenzylidene)benzohydrazide (**6**)

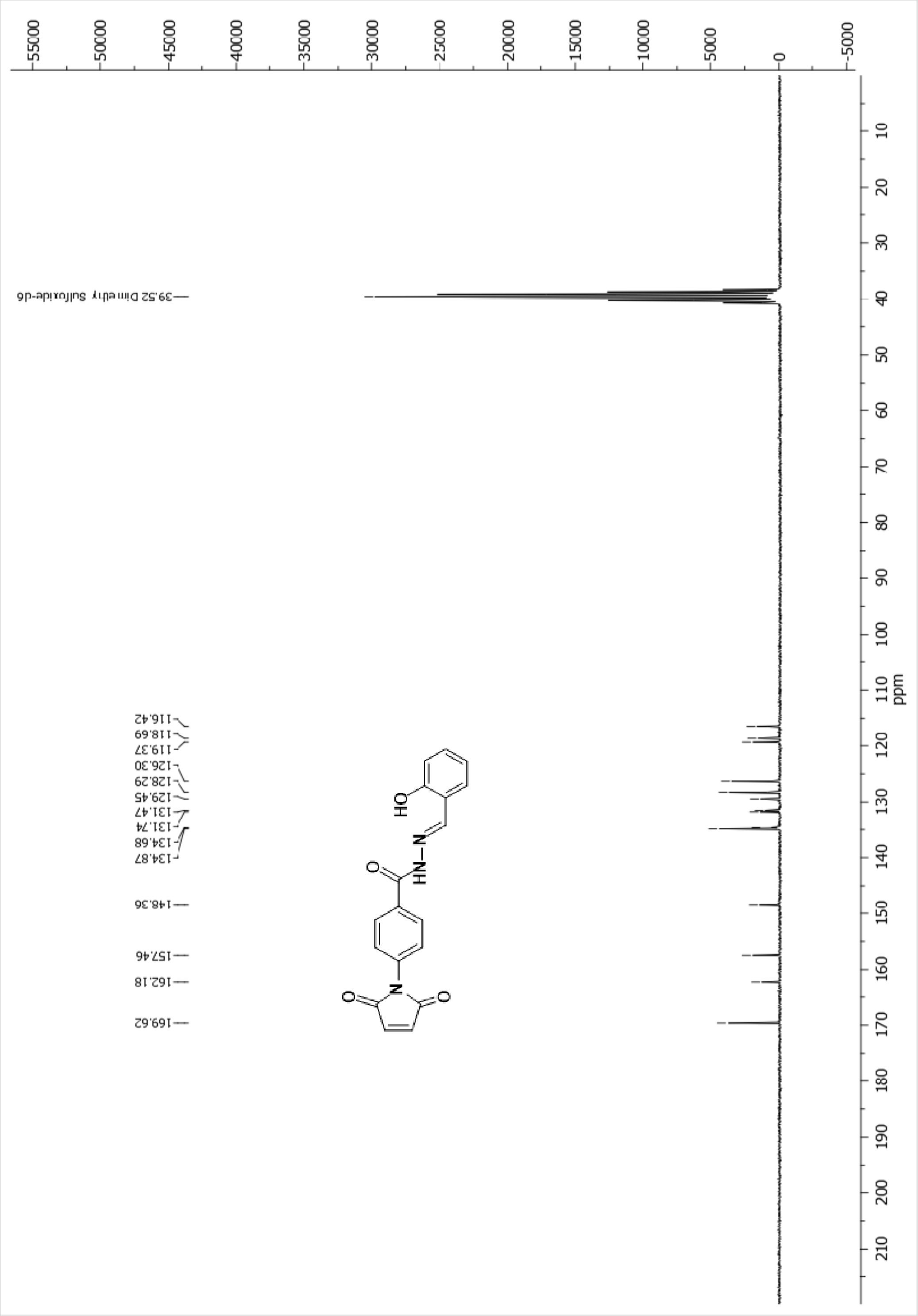

^{1}H NMR: 4-(2,5-dioxo-2,5-dihydro-1*H*-pyrrol-1-yl)-*N*-(3-hydroxybenzylidene)benzohydrazide (**7**)

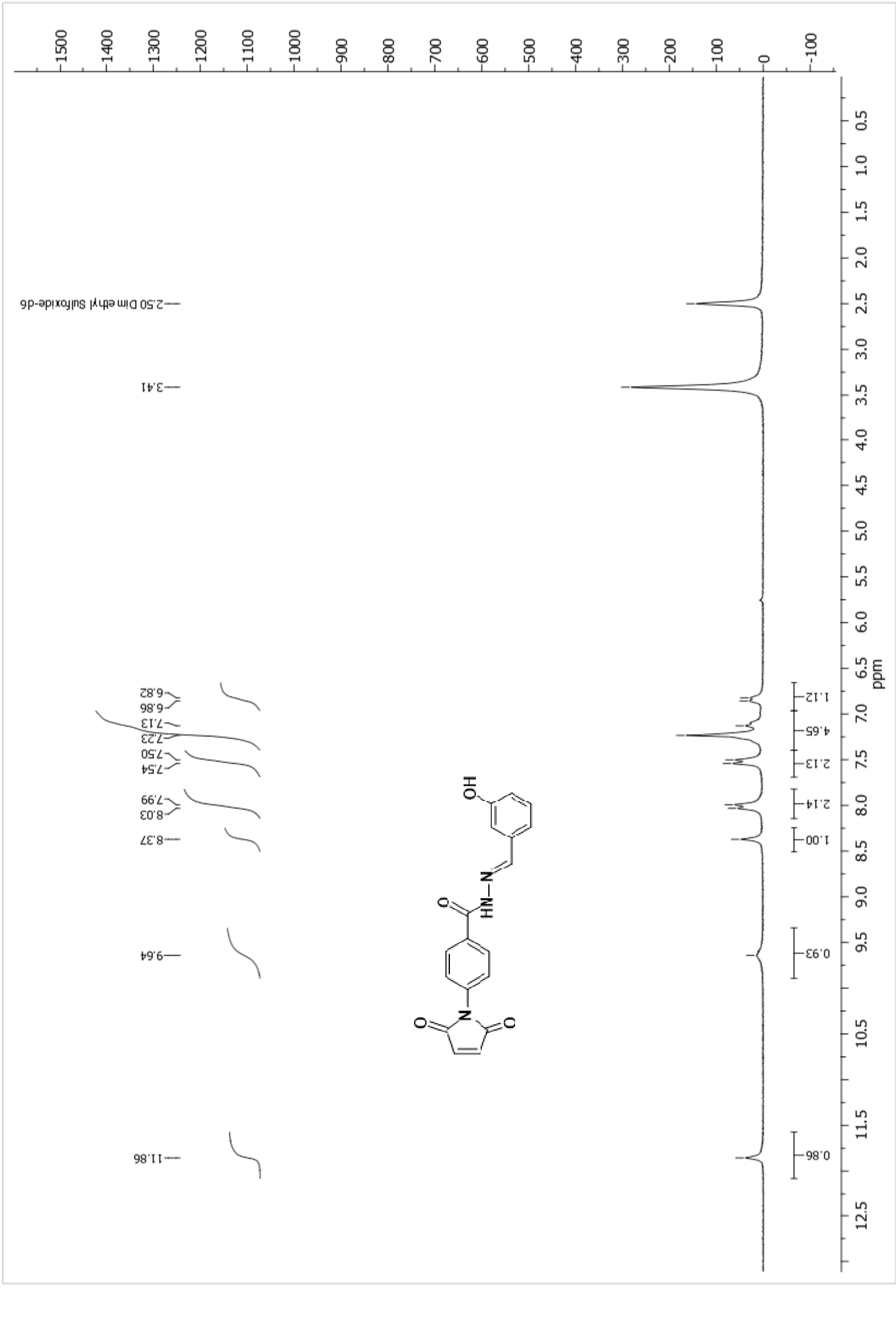

^{13}C NMR: 4-(2,5-dioxo-2,5-dihydro-1*H*-pyrrol-1-yl)-*N*-(3-hydroxybenzylidene)benzohydrazide (**7**)

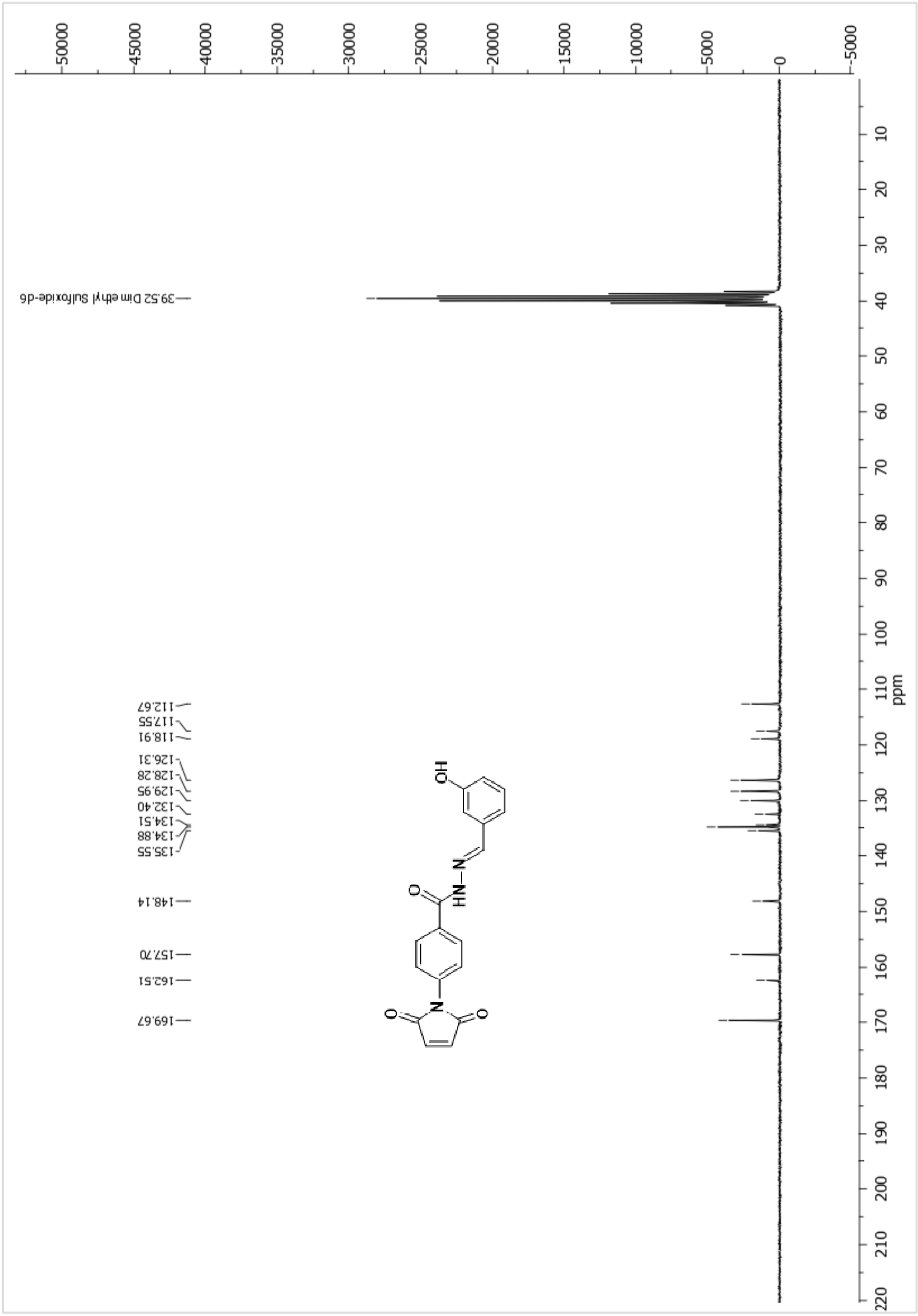

^{1}H NMR: 4-(2,5-dioxo-2,5-dihydro-1*H*-pyrrol-1-yl)-*N*-(4-hydroxybenzylidene)benzohydrazide (**8**)

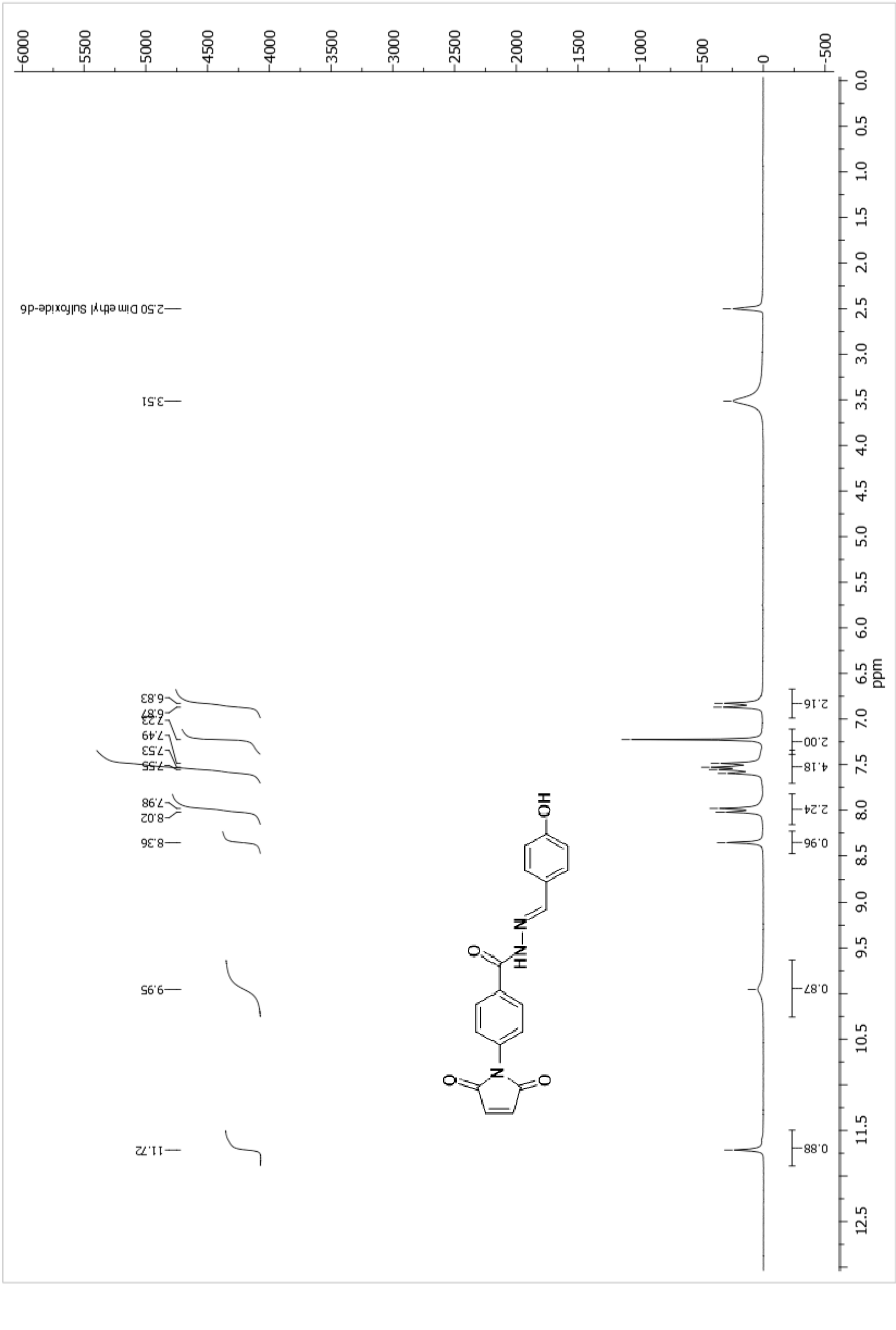

^{13}C NMR: 4-(2,5-dioxo-2,5-dihydro-1*H*-pyrrol-1-yl)-*N*-(4-hydroxybenzylidene)benzohydrazide (**8**)

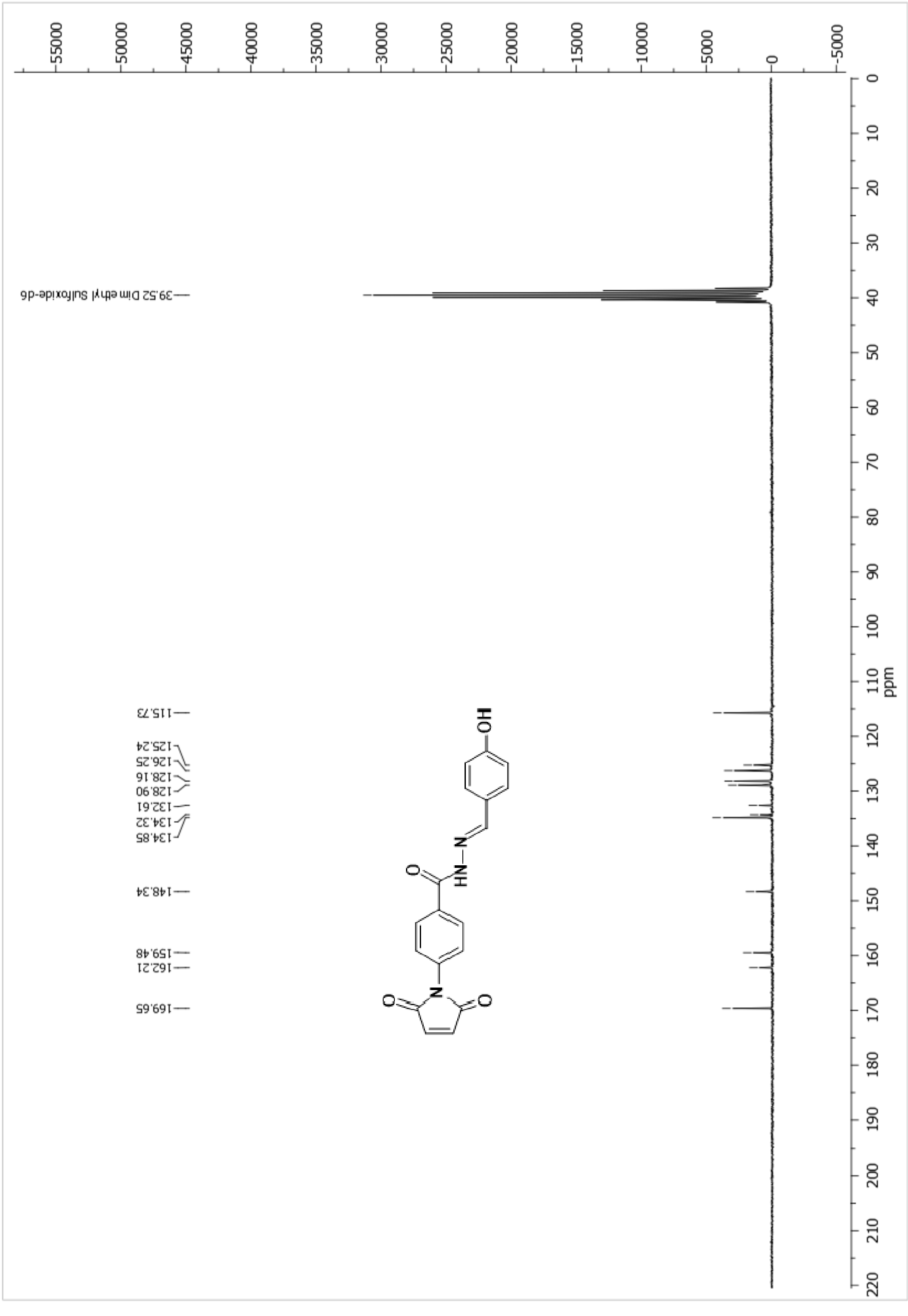

^{1}H NMR: 4-(2,5-dioxo-2,5-dihydro-1*H*-pyrrol-1-yl)-*N*-(2-methoxybenzylidene)benzohydrazide (**9**)

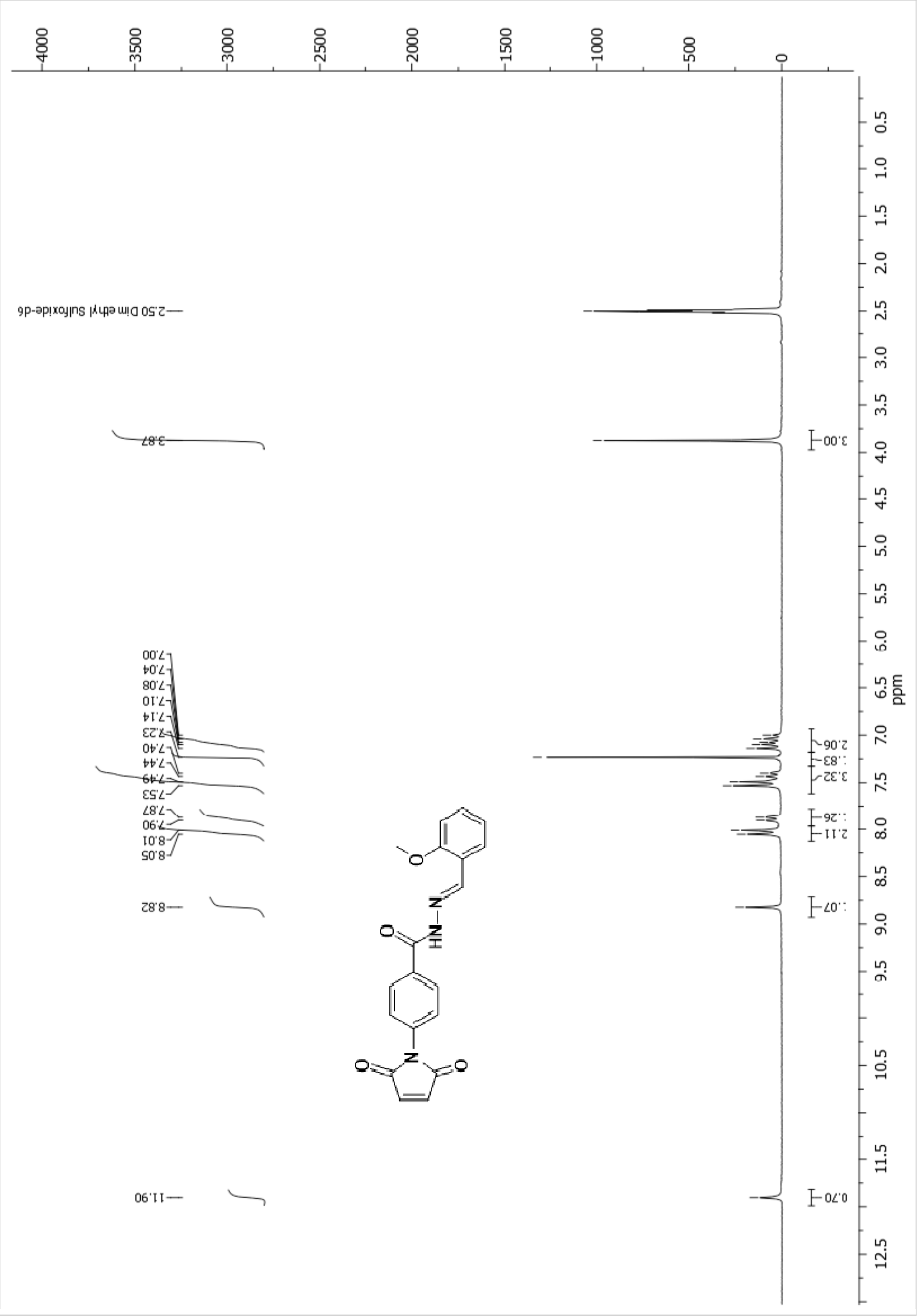

^{13}C NMR: 4-(2,5-dioxo-2,5-dihydro-1*H*-pyrrol-1-yl)-*N*-(2-methoxybenzylidene)benzohydrazide (**9**)

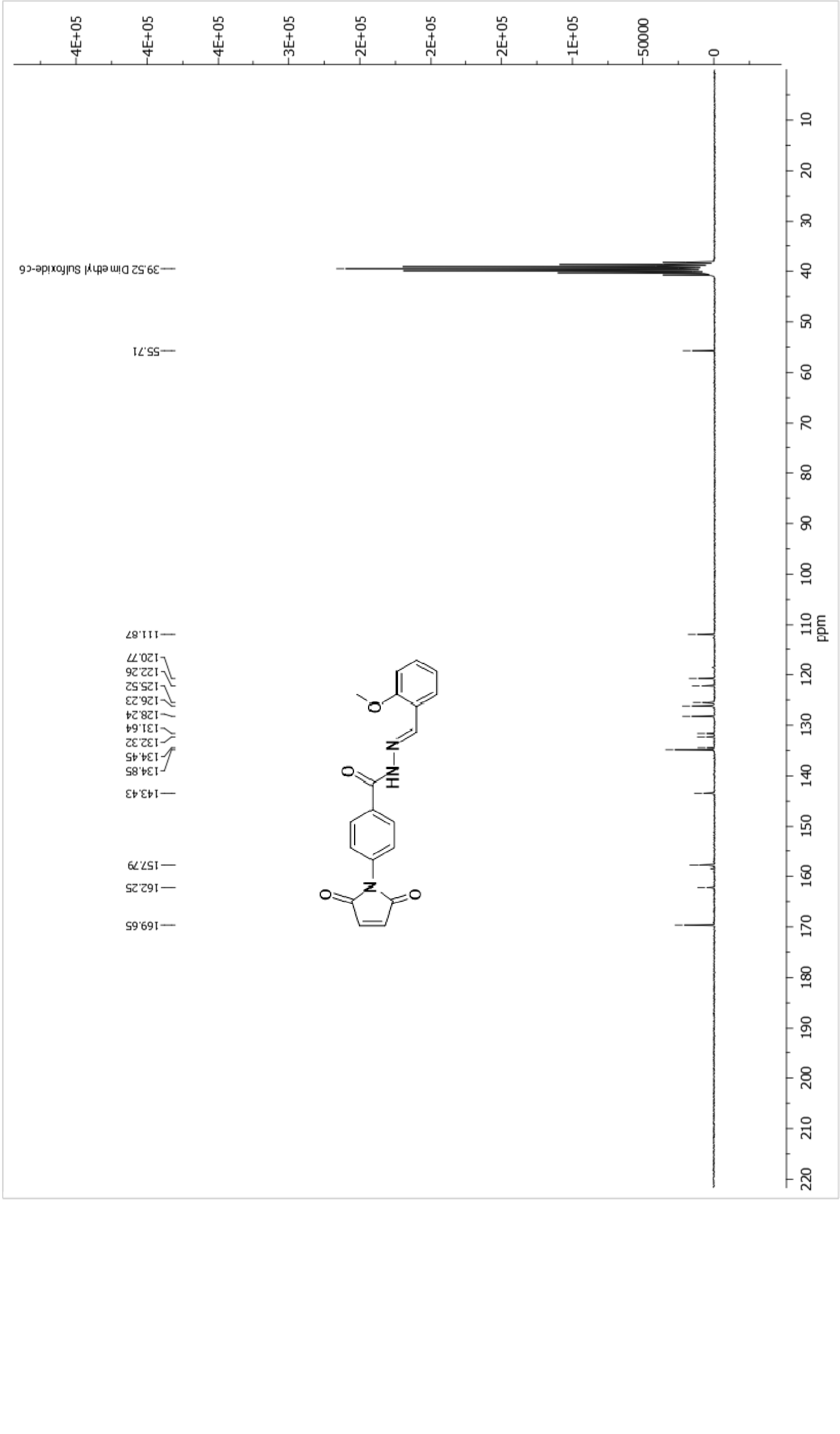

^{1}H NMR: 4-(2,5-dioxo-2,5-dihydro-1*H*-pyrrol-1-yl)-*N*-(3-methoxybenzylidene)benzohydrazide (**10**)

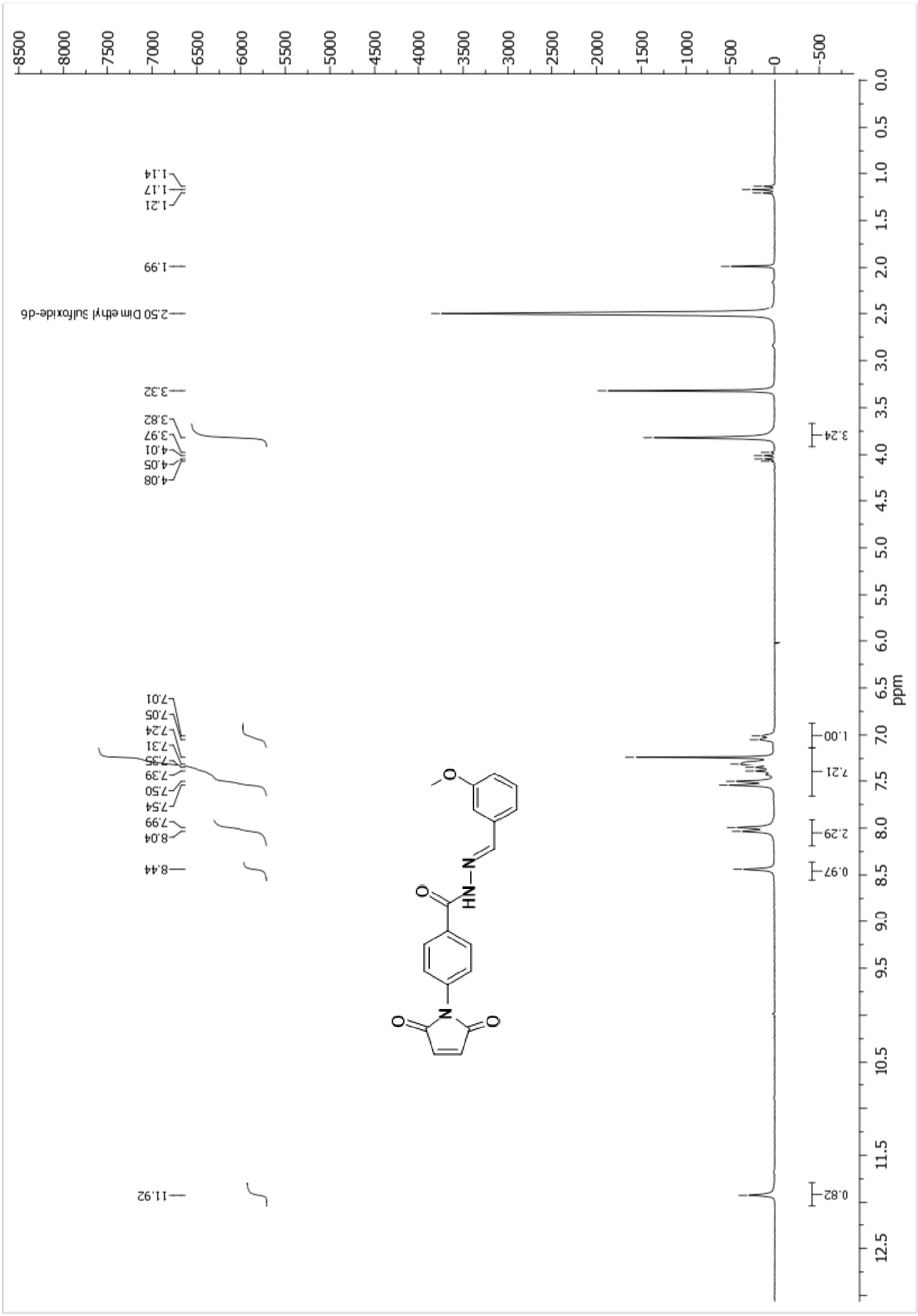

^{13}C NMR: 4-(2,5-dioxo-2,5-dihydro-1*H*-pyrrol-1-yl)-*N*-(3-methoxybenzylidene)benzohydrazide (**10**)

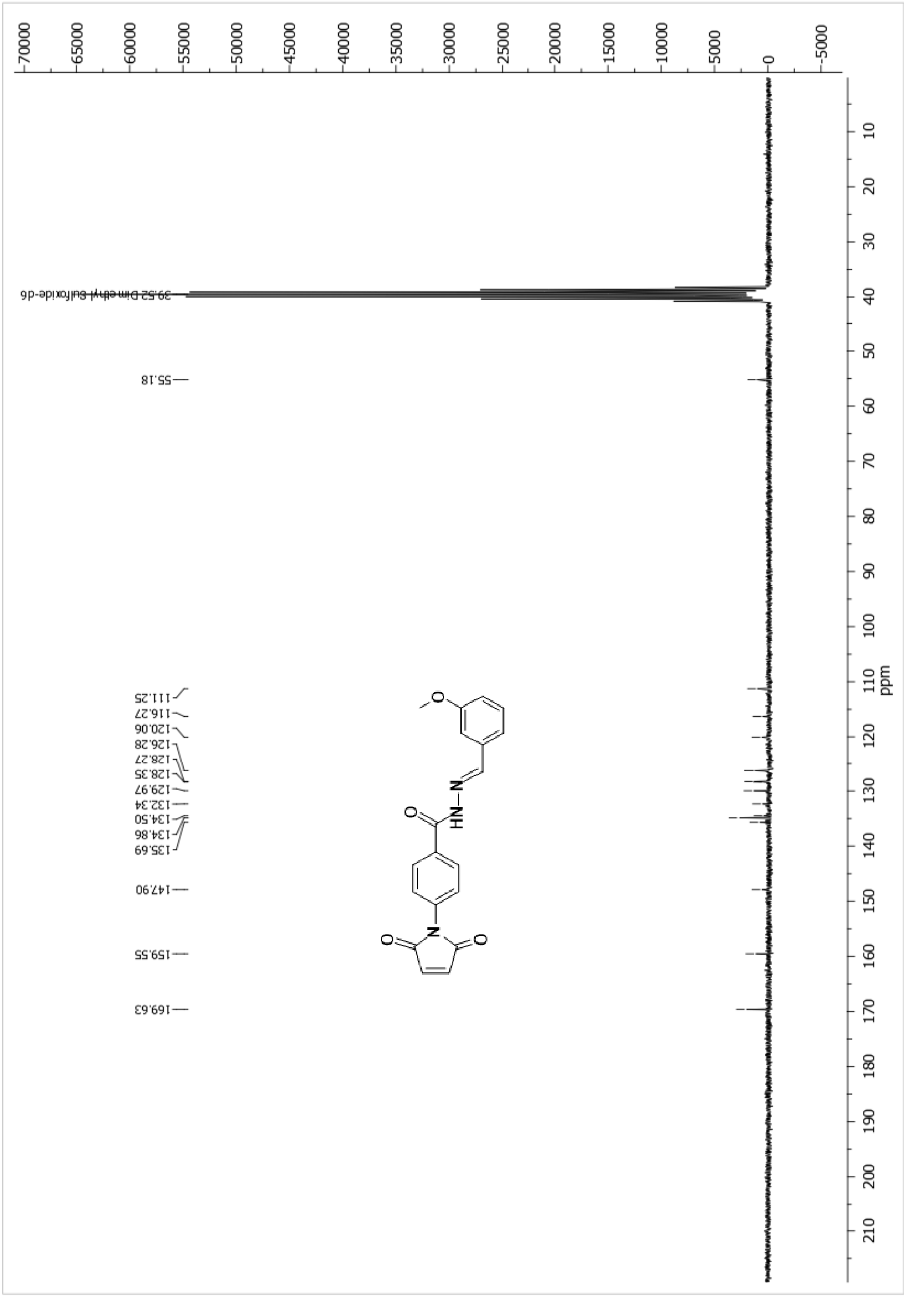

^{1}H NMR: 4-(2,5-dioxo-2,5-dihydro-1*H*-pyrrol-1-yl)-*N*-(2-nitrobenzylidene)benzohydrazide (**11**)

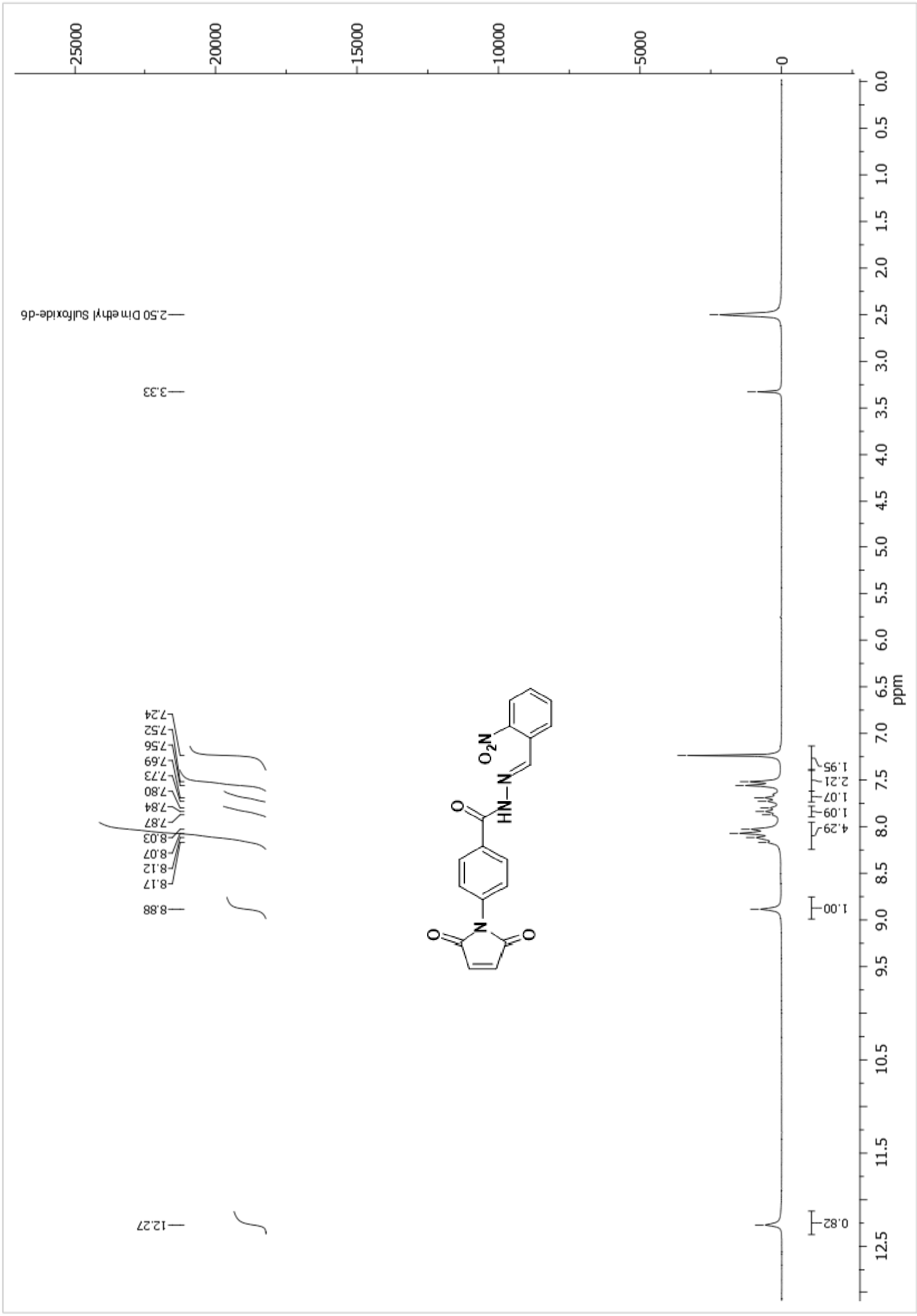

^{13}C NMR: 4-(2,5-dioxo-2,5-dihydro-1*H*-pyrrol-1-yl)-*N*-(2-nitrobenzylidene)benzohydrazide (**11**)

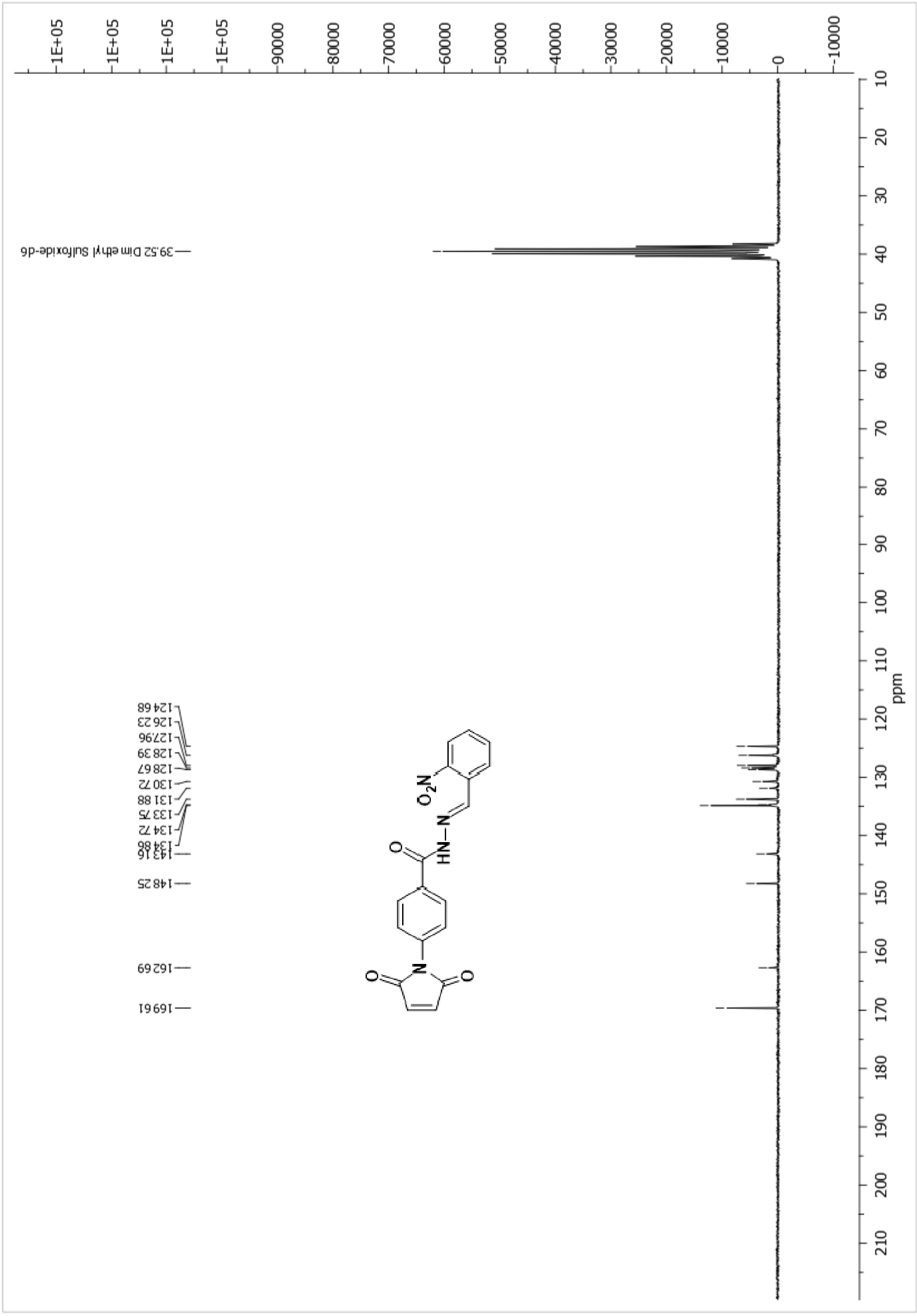

^{1}H NMR: 4-(2,5-dioxo-2,5-dihydro-1*H*-pyrrol-1-yl)-*N*-(3-nitrobenzylidene)benzohydrazide (**12**)

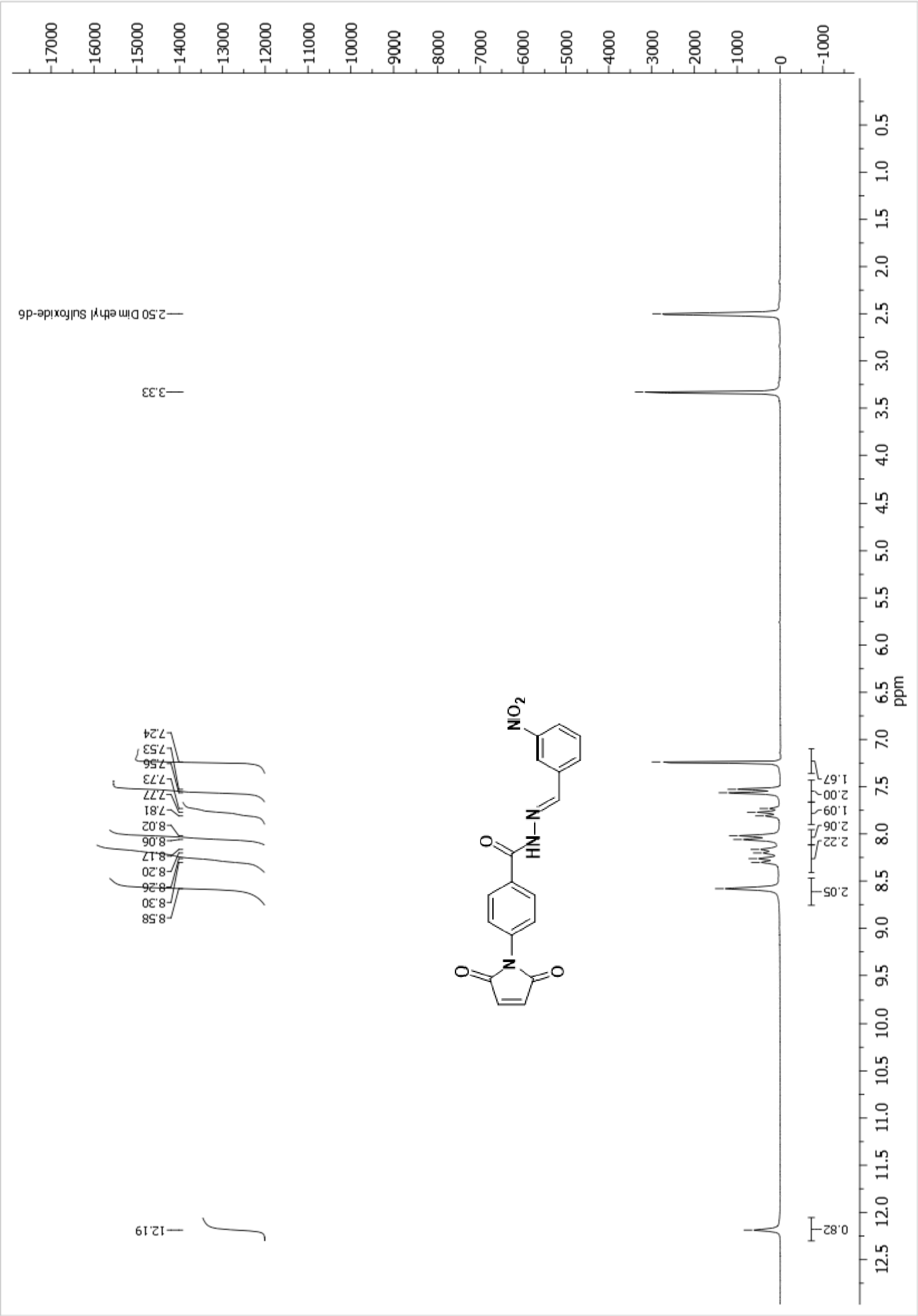

^{13}C NMR: 4-(2,5-dioxo-2,5-dihydro-1*H*-pyrrol-1-yl)-*N*-(3-nitrobenzylidene)benzohydrazide (**12**)

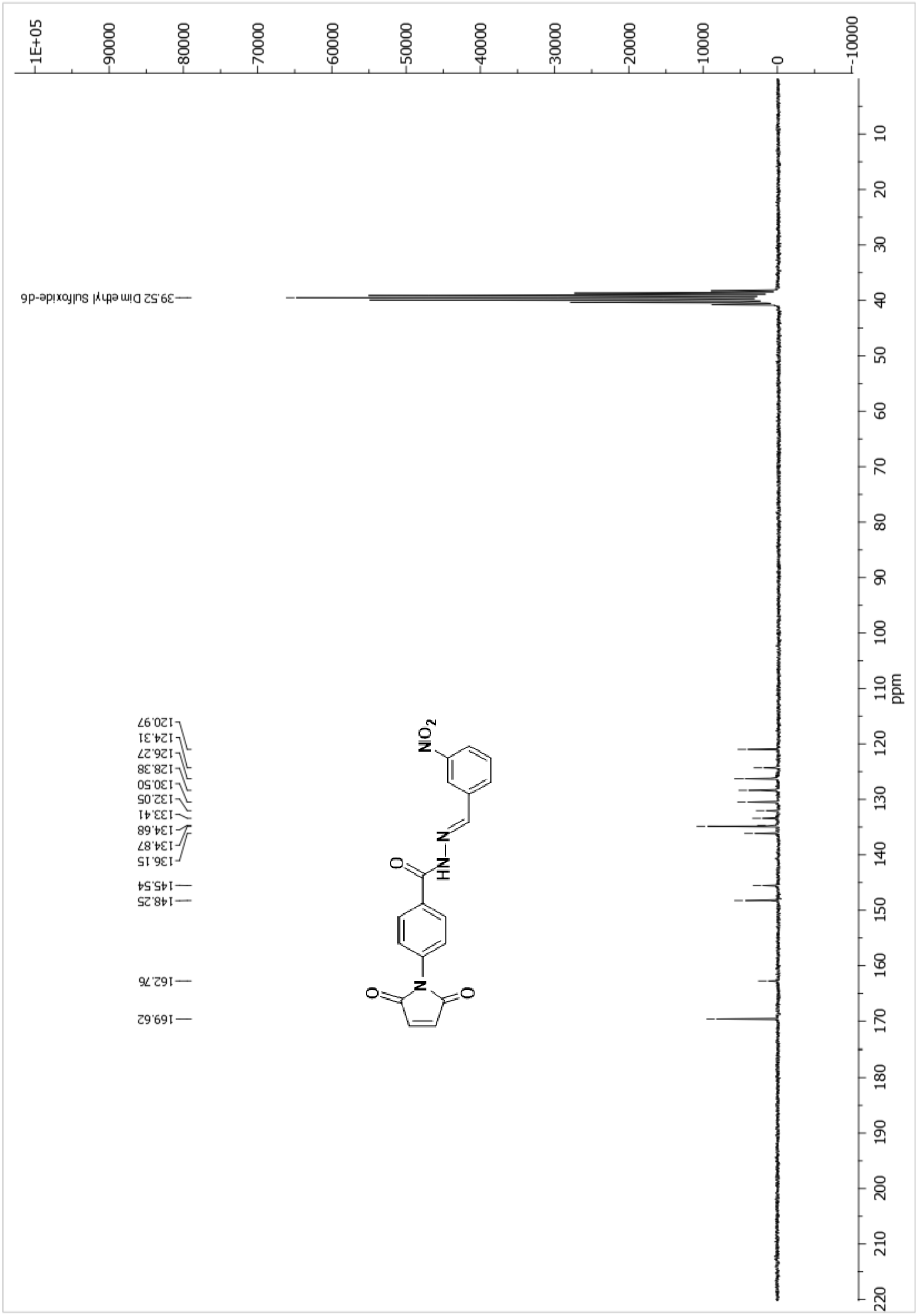

^{1}H NMR: 4-(2,5-dioxo-2,5-dihydro-1*H*-pyrrol-1-yl)-*N*-(4-bromobenzylidene)benzohydrazide (**13**)

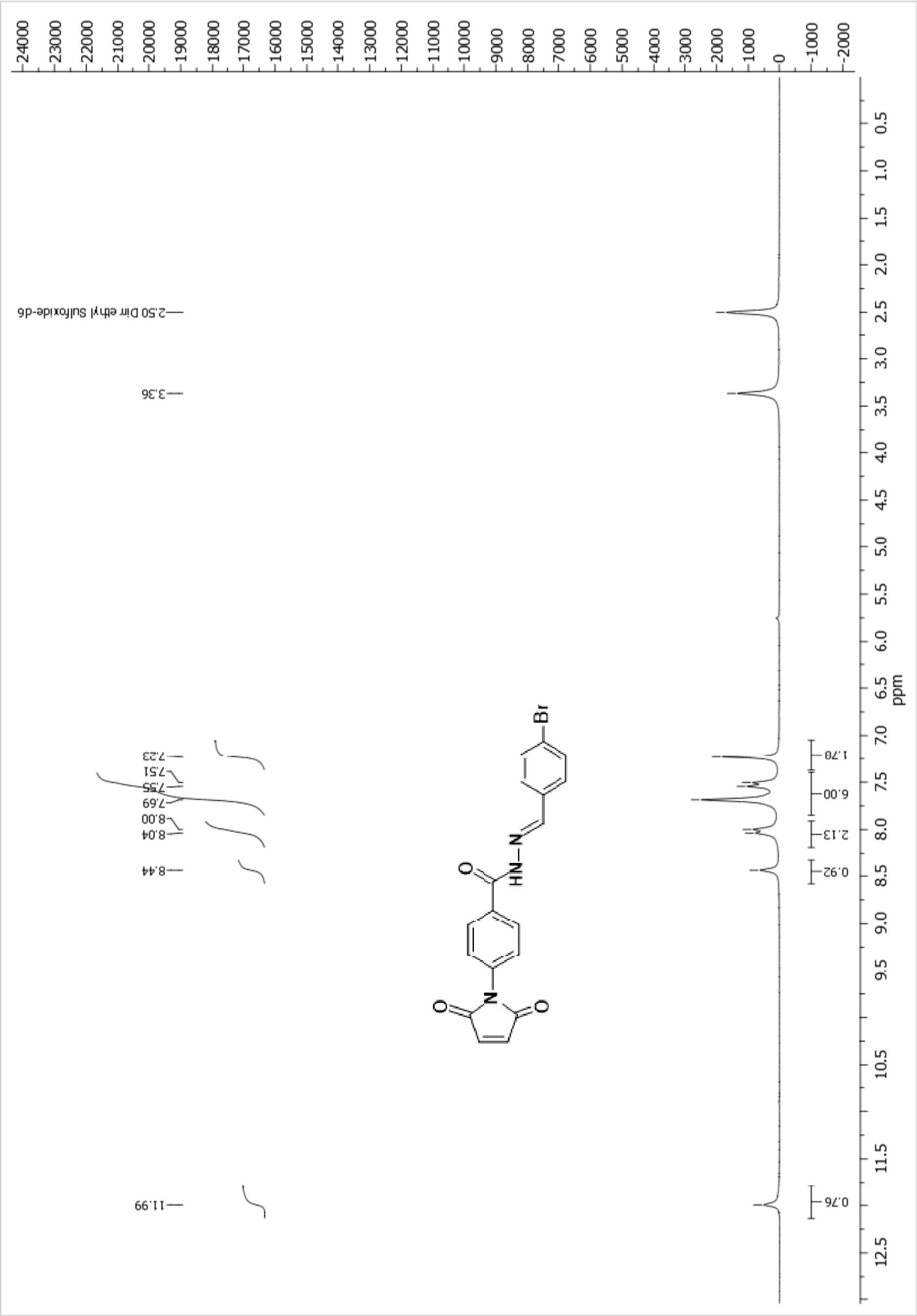

^{13}C NMR: 4-(2,5-dioxo-2,5-dihydro-1*H*-pyrrol-1-yl)-*N*-(4-bromobenzylidene)benzohydrazide (**13**)

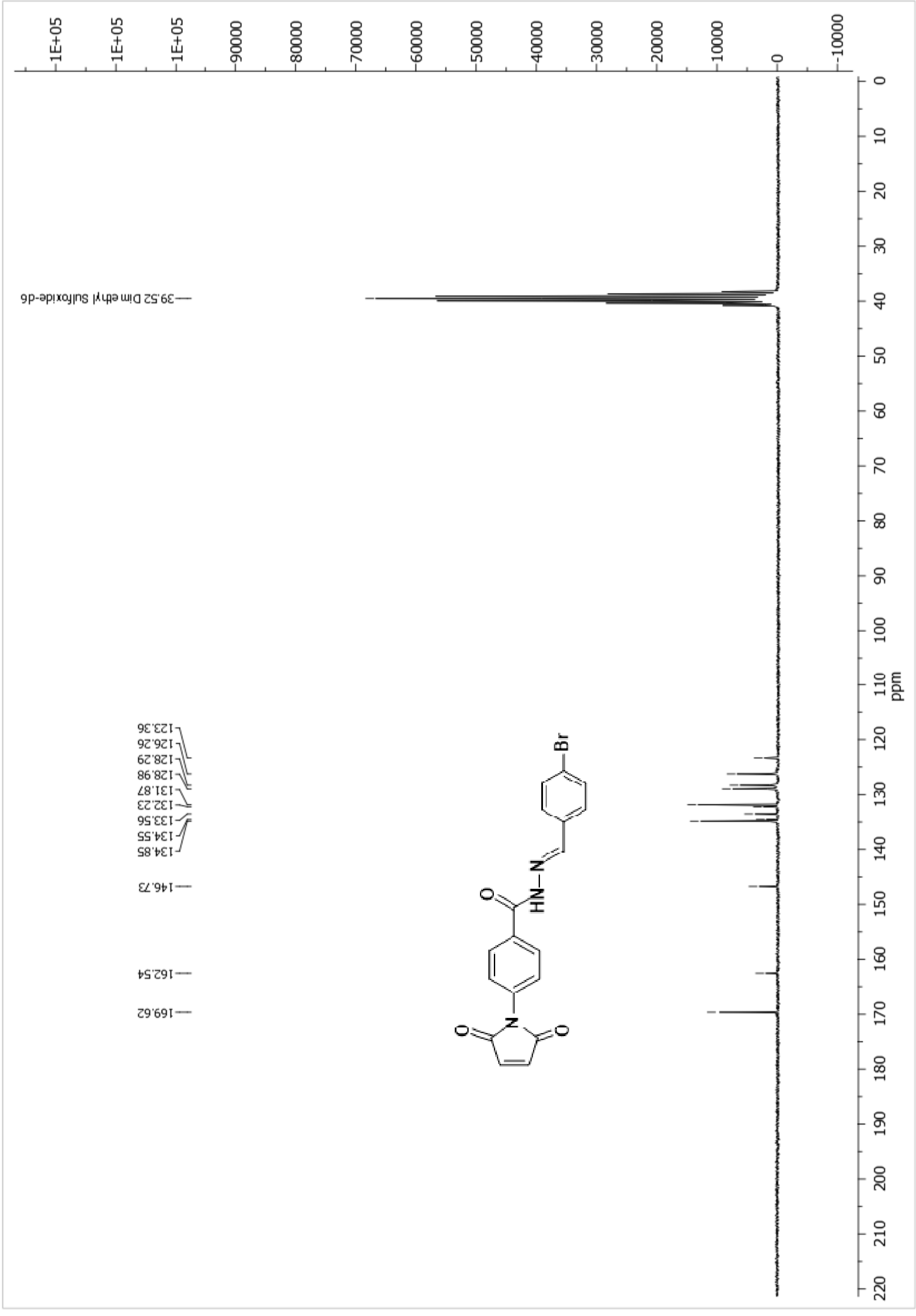

^{1}H NMR: 4-(2,5-dioxo-2,5-dihydro-1*H*-pyrrol-1-yl)-*N*-(2-hydroxy-3-methoxybenzylidene)benzohydrazide (**14**)

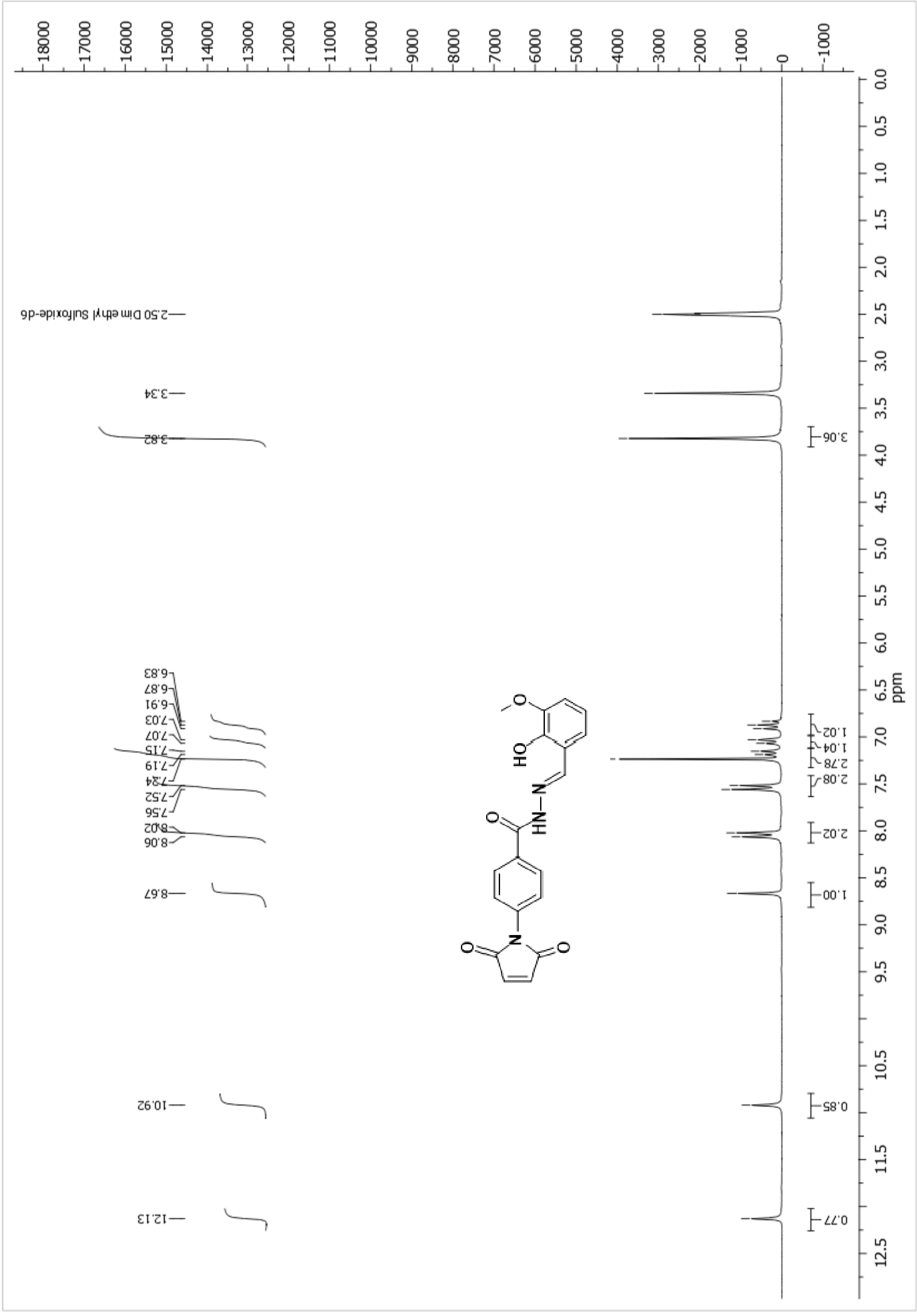

^{13}C NMR: 4-(2,5-dioxo-2,5-dihydro-1*H*-pyrrol-1-yl)-*N*-(2-hydroxy-3-methoxybenzylidene)benzohydrazide (**14**)

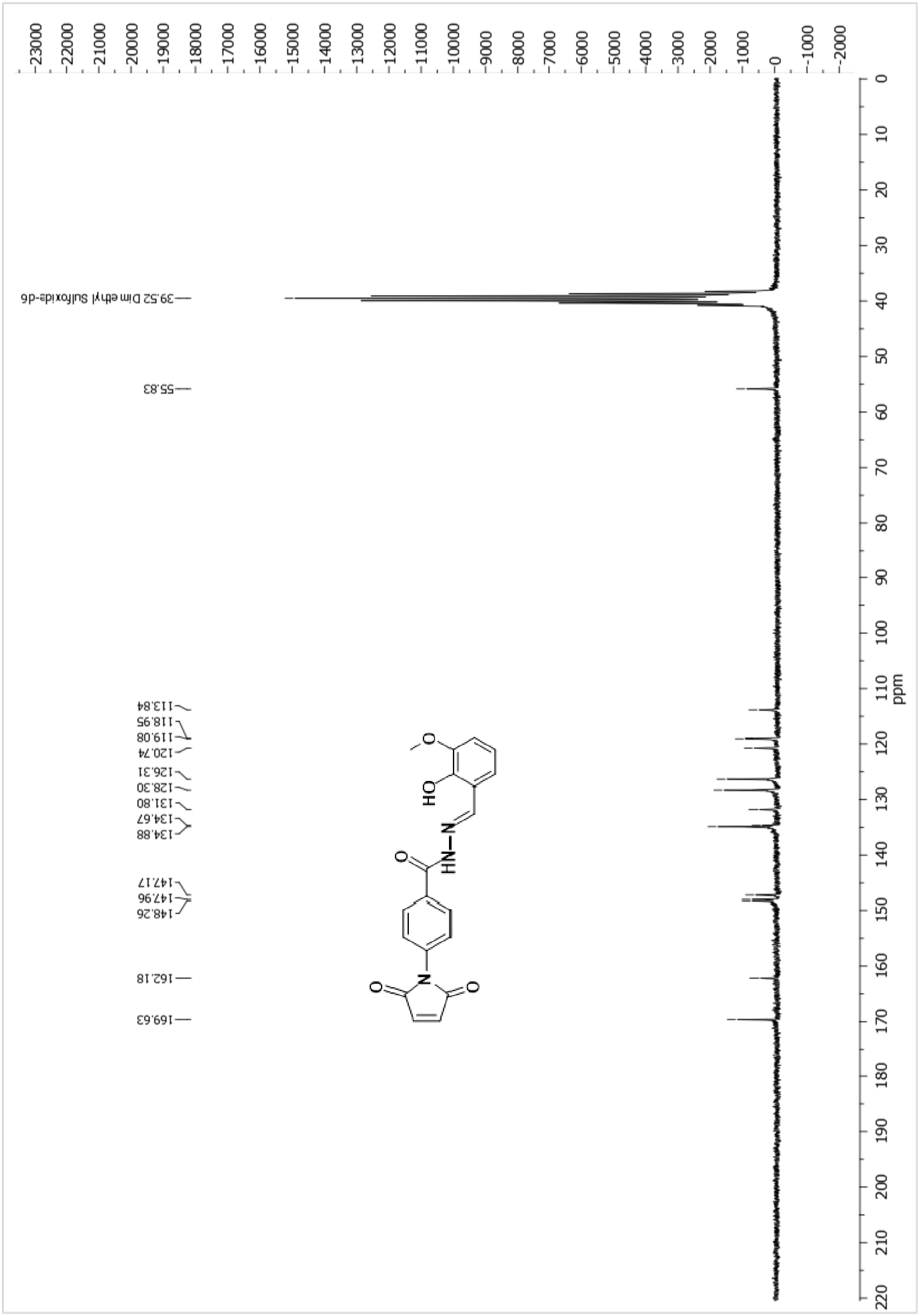

^{1}H NMR: 4-(2,5-dioxo-2,5-dihydro-1*H*-pyrrol-1-yl)-*N*-(3-hydroxy-4-methoxybenzelidene)benzohydrazide (**15**)

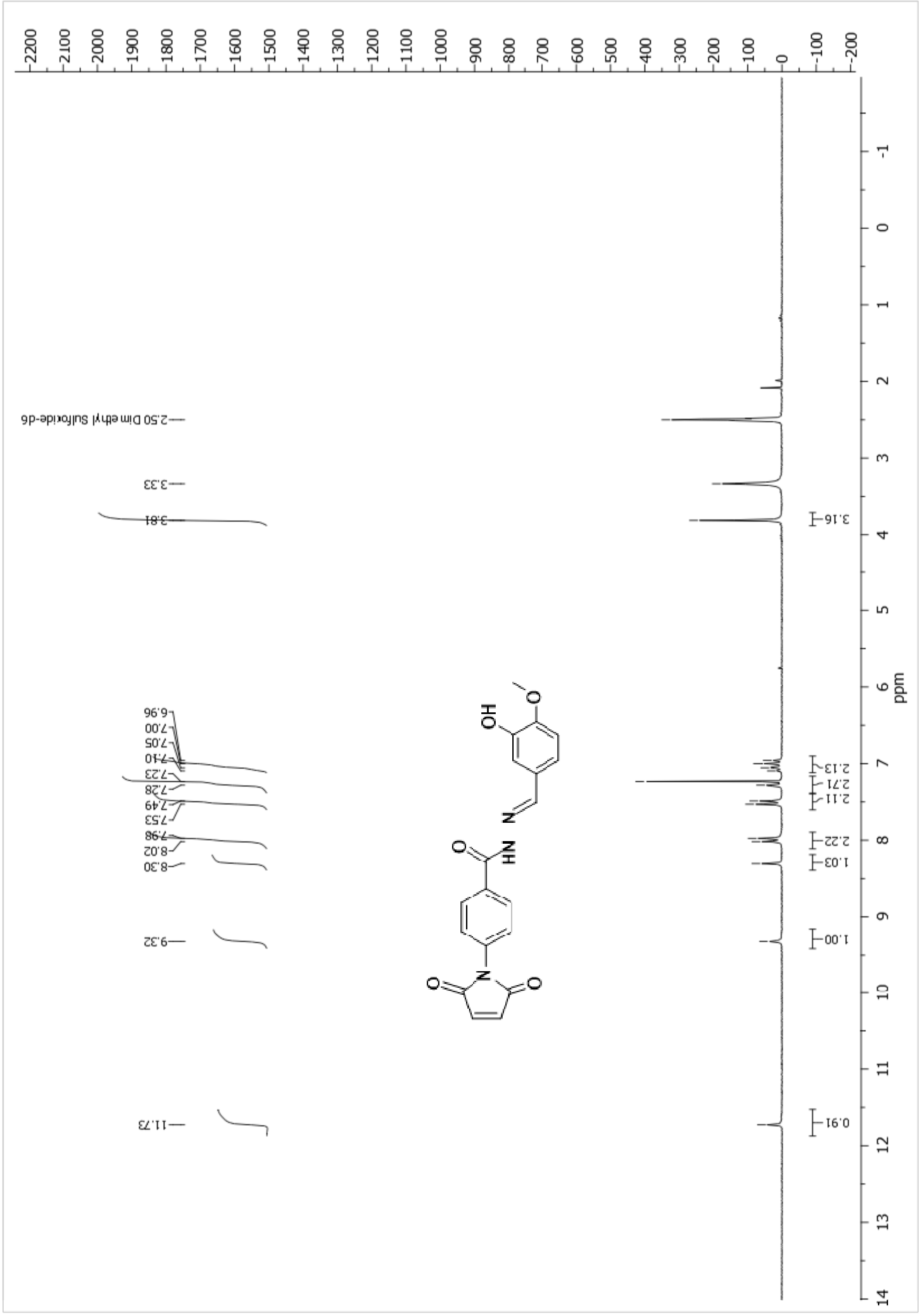

^{13}C NMR: 4-(2,5-dioxo-2,5-dihydro-1*H*-pyrrol-1-yl)-*N*-(3-hydroxy-4-methoxybenzelidene)benzohydrazide (**15**)

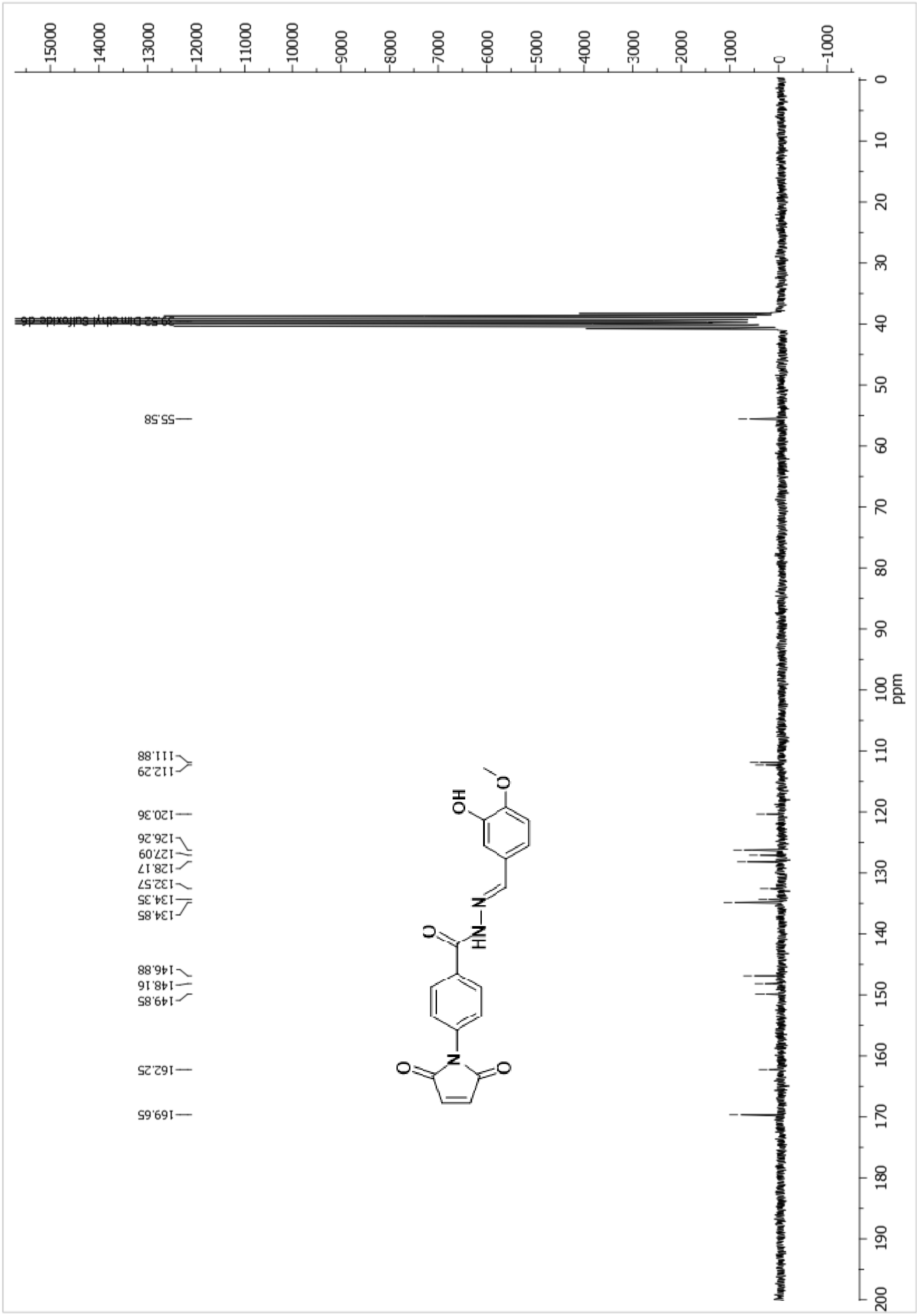

^{1}H NMR: 4-(2,5-dioxo-2,5-dihydro-1*H*-pyrrol-1-yl)-*N*-(4-hydroxy-3-methoxybenzelidene)benzohydrazide (**16**)

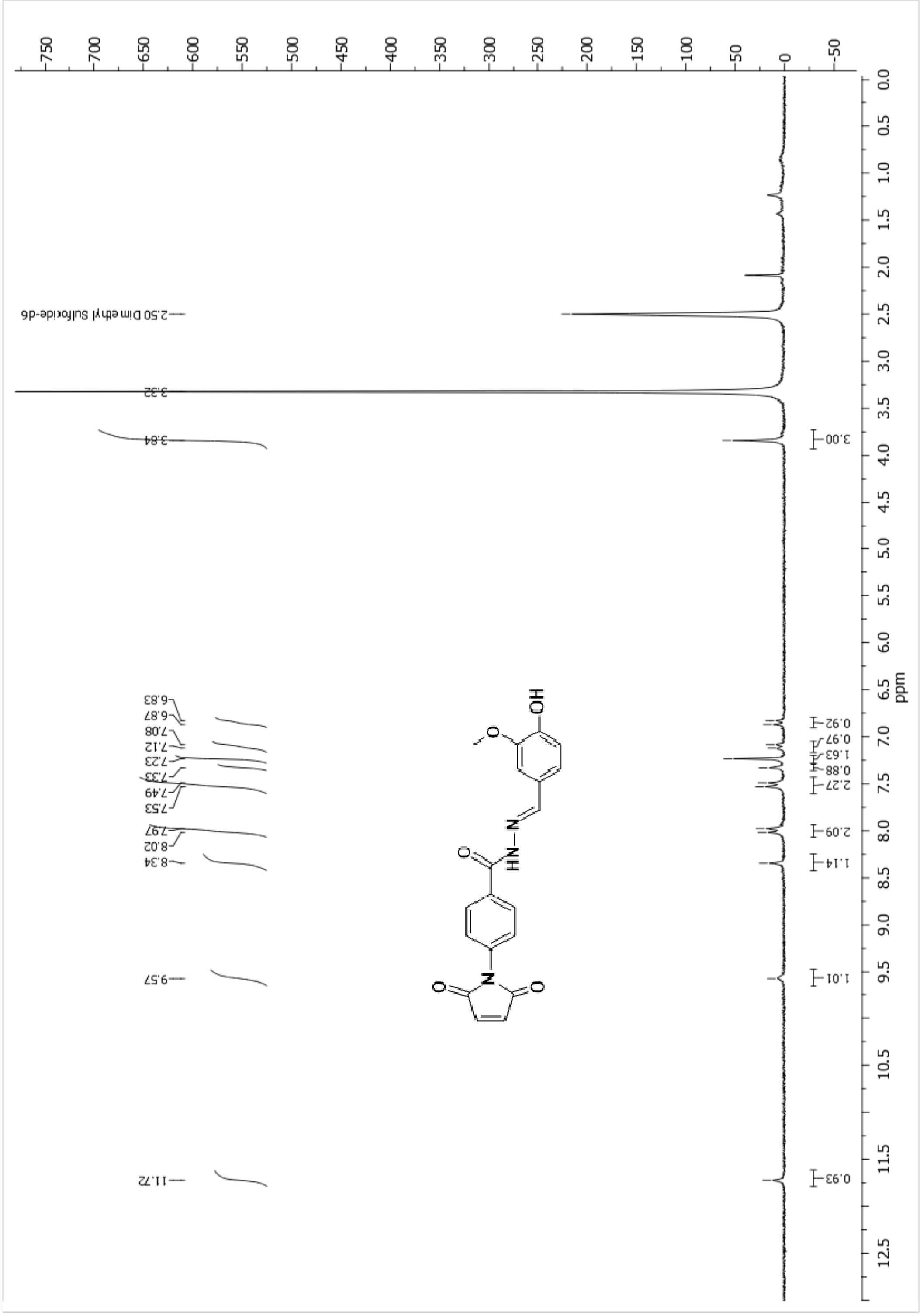

^{13}C NMR: 4-(2,5-dioxo-2,5-dihydro-1*H*-pyrrol-1-yl)-*N*-(4-hydroxy-3-methoxybenzelidene)benzohydrazide (**16**)

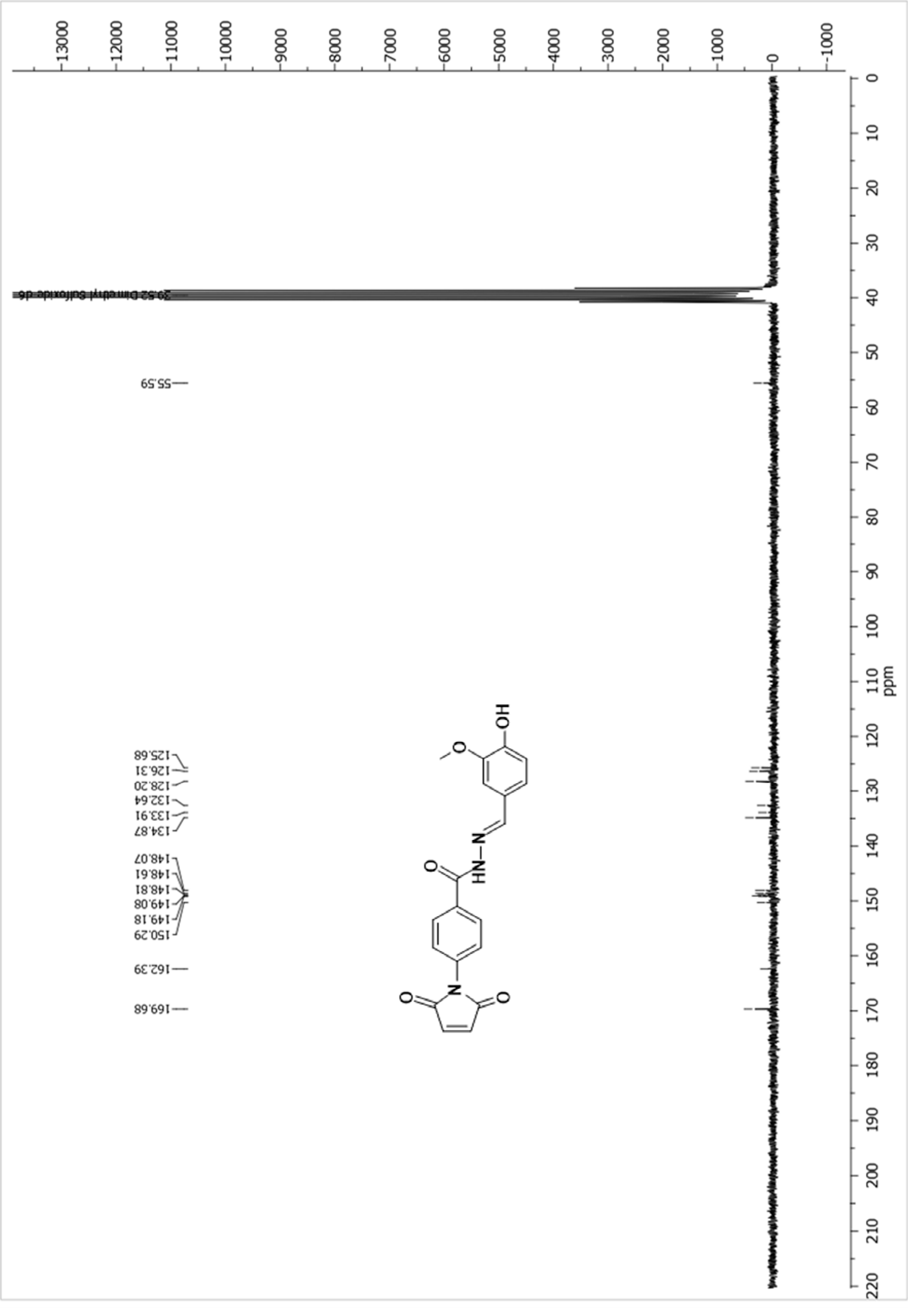

^{1}H NMR: 4-(2,5-dioxo-2,5-dihydro-1*H*-pyrrol-1-yl)-*N*-(furan-2-ylmethylene)benzohydrazide (**17**)

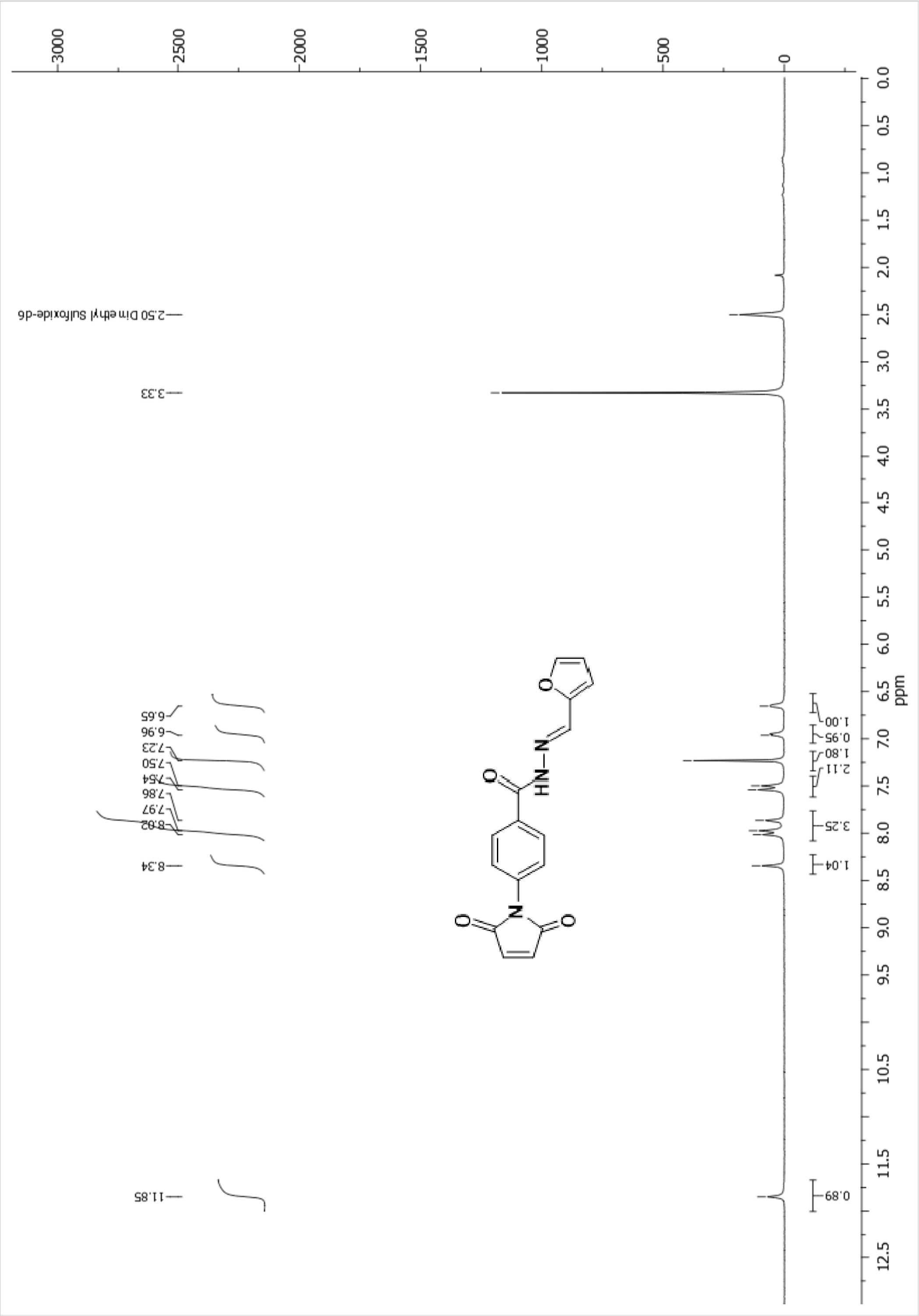

^{13}C NMR: 4-(2,5-dioxo-2,5-dihydro-1*H*-pyrrol-1-yl)-*N*-(furan-2-ylmethylene)benzohydrazide (**17**)

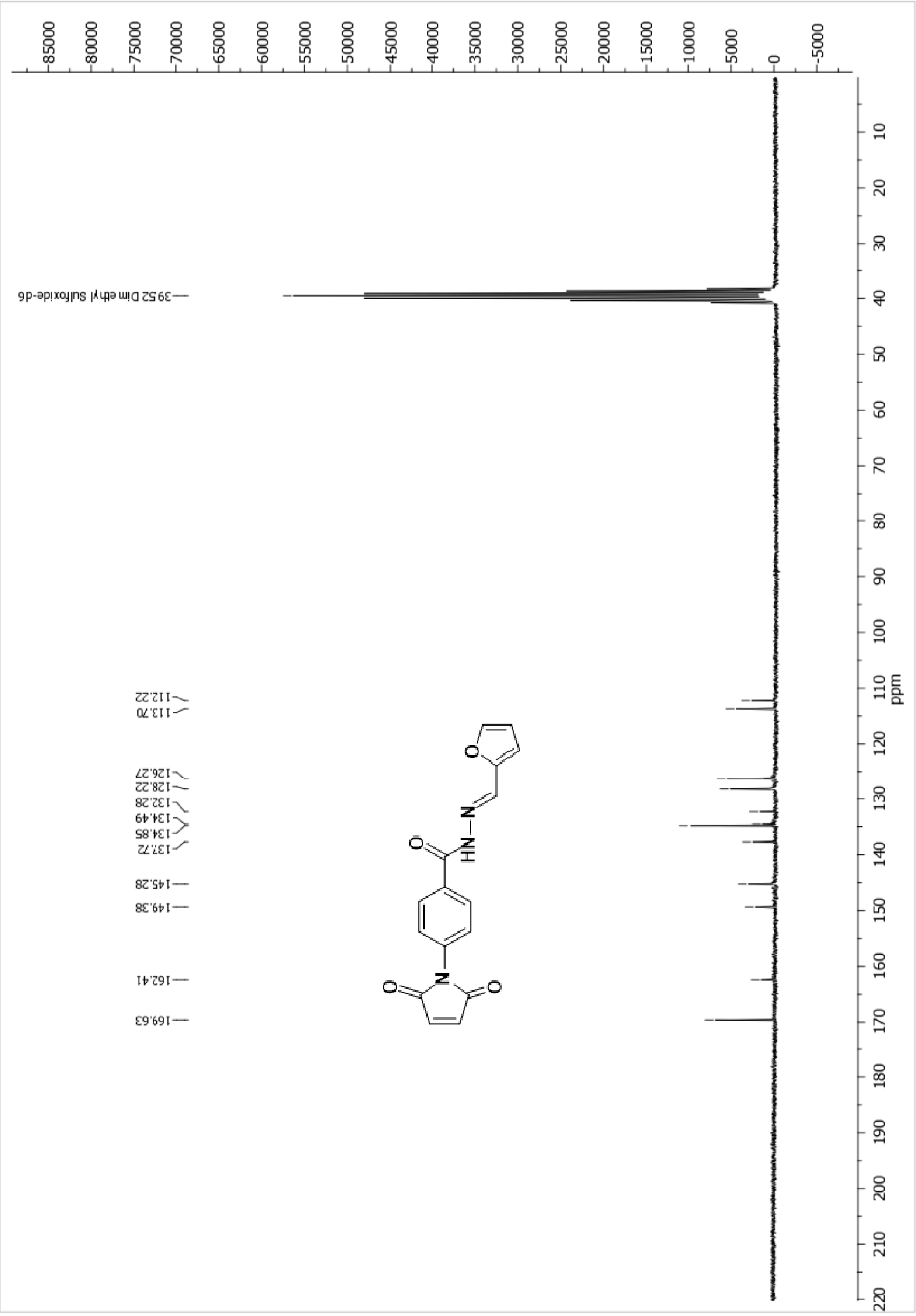

^{1}H NMR: 4-(2,5-dioxo-2,5-dihydro-1*H*-pyrrol-1-yl)-*N*-(furan-3-ylmethylene)benzohydrazide (**18**)

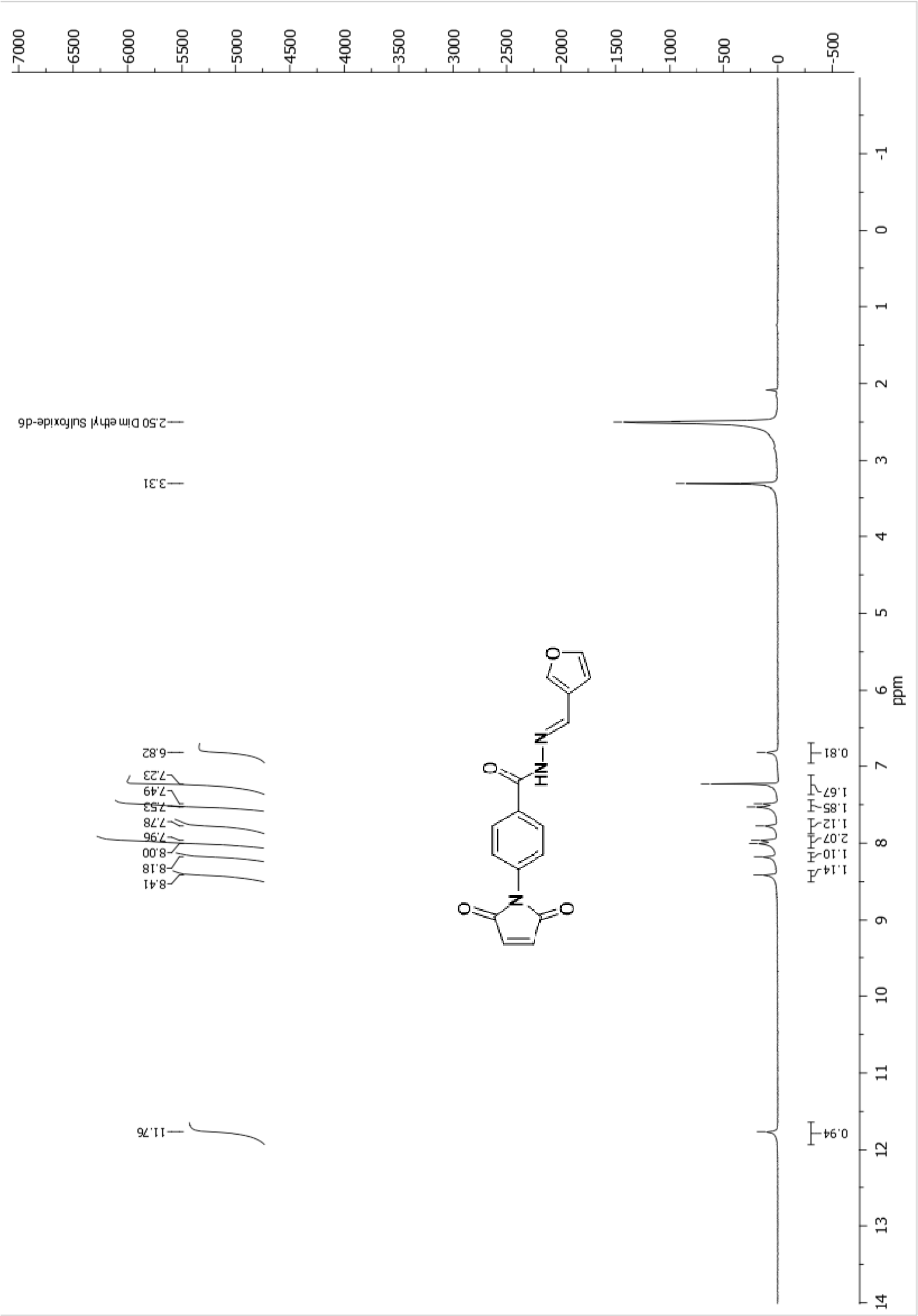

^{13}C NMR: 4-(2,5-dioxo-2,5-dihydro-1*H*-pyrrol-1-yl)-*N*-(furan-3-ylmethylene)benzohydrazide (**18**)

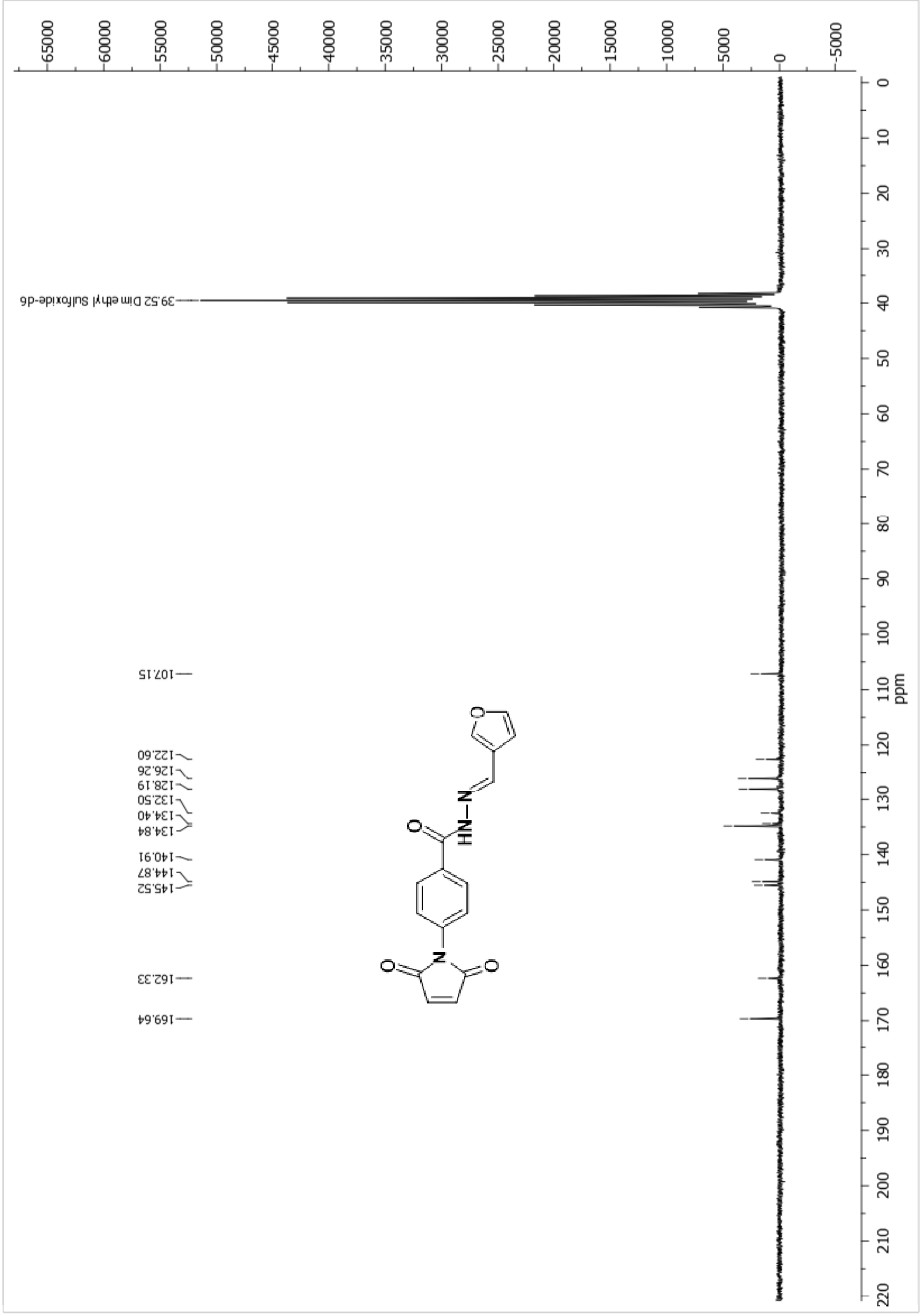

^{1}H NMR: 4-(2,5-dioxo-2,5-dihydro-1*H*-pyrrol-1-yl)-*N*-(5-methylfuran-2-ylmethylene)benzohydrazide (**19**)

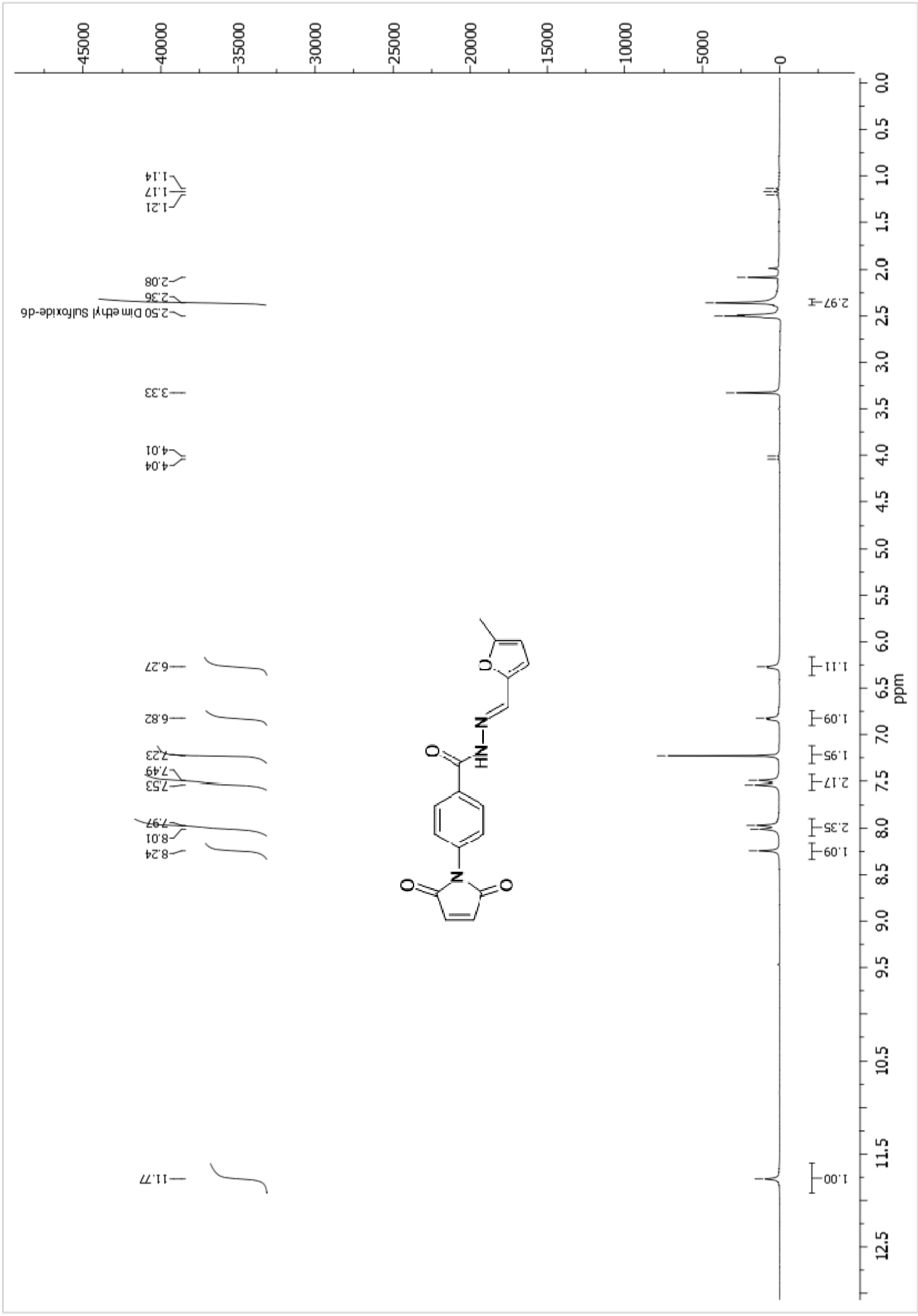

^{13}C NMR: 4-(2,5-dioxo-2,5-dihydro-1*H*-pyrrol-1-yl)-*N*-(5-methylfuran-2-ylmethylene)benzohydrazide (**19**)

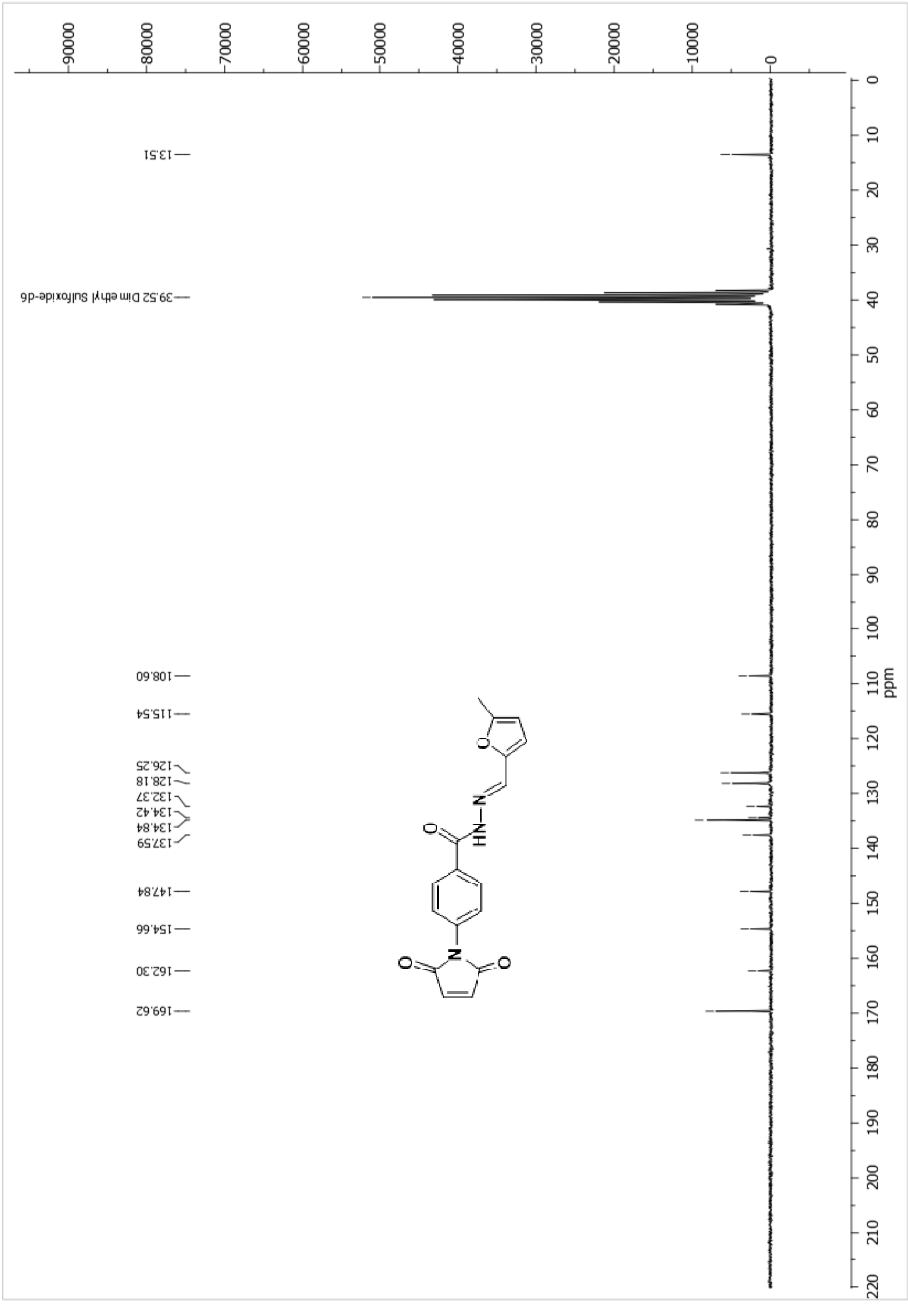

^{1}H NMR: 4-(2,5-dioxo-2,5-dihydro-1*H*-pyrrol-1-yl)-*N*-(5-nitrofuran-2-ylmethylene)benzohydrazide (**20**)

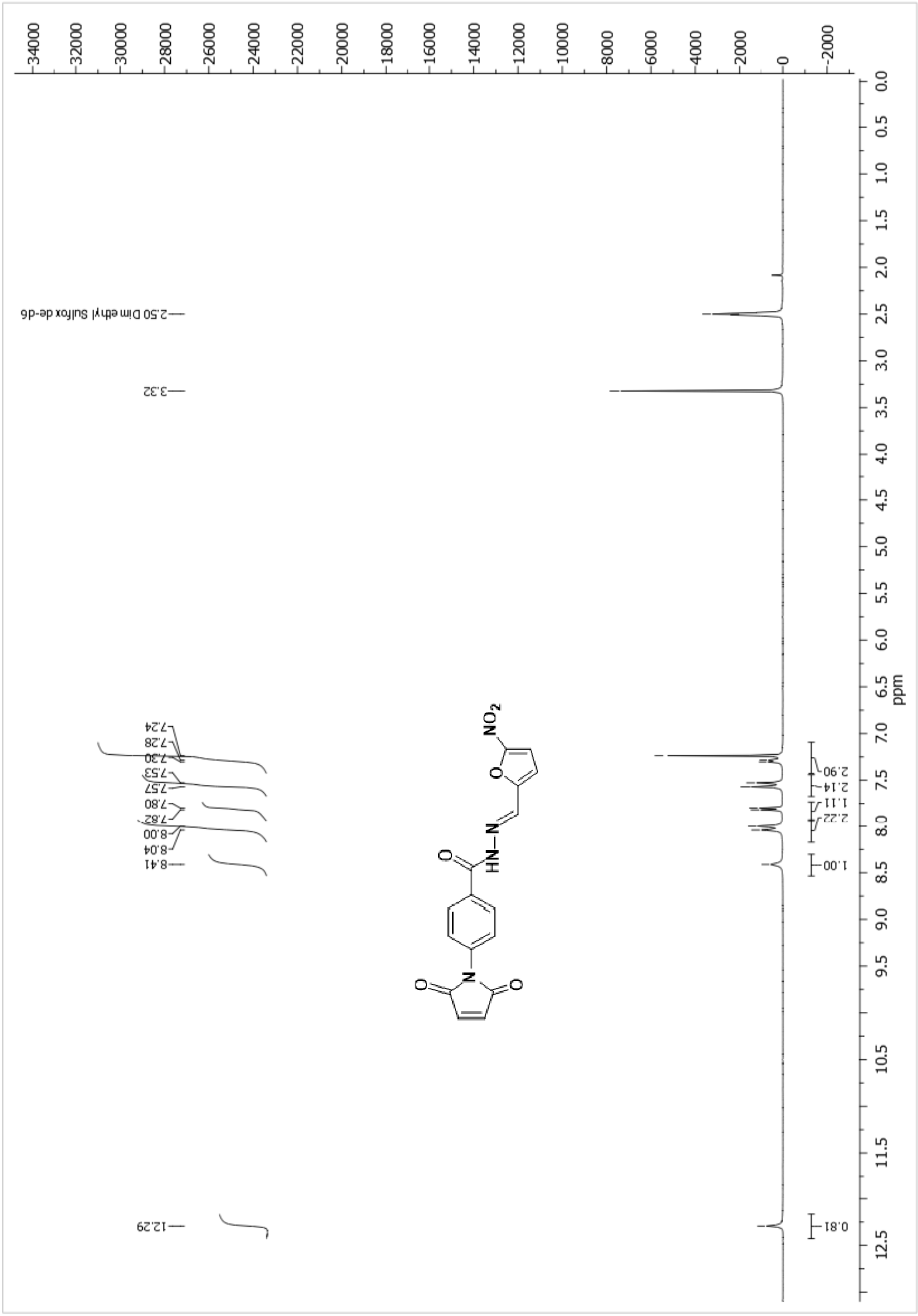

^{13}C NMR: 4-(2,5-dioxo-2,5-dihydro-1*H*-pyrrol-1-yl)-*N*-(5-nitrofuran-2-ylmethylene)benzohydrazide (**20**)

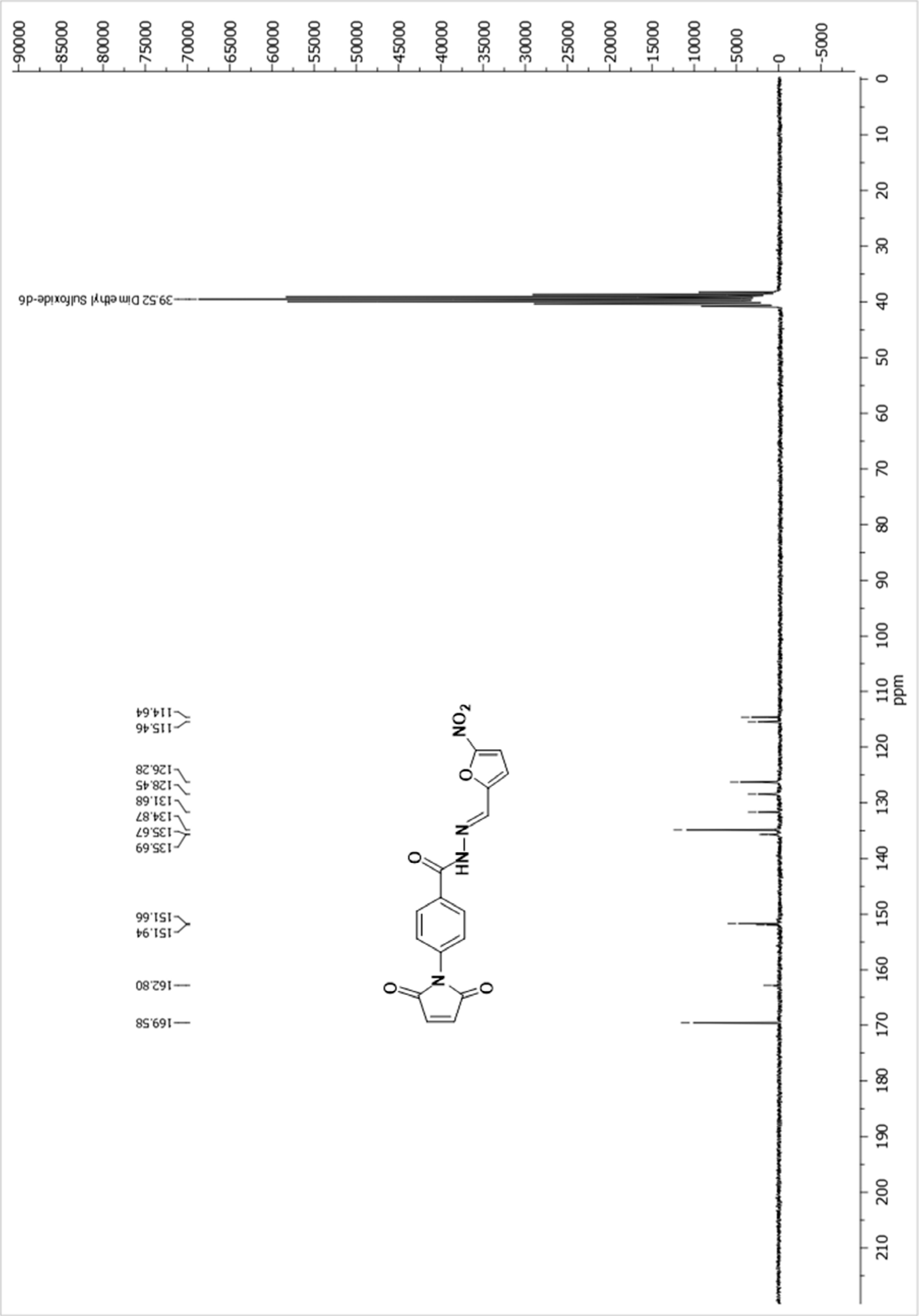

^{1}H NMR: 4-(2,5-dioxo-2,5-dihydro-1*H*-pyrrol-1-yl)-*N*-(5-(hydroxymethyl)furan-2-ylmethylene)benzohydrazide (**21**)

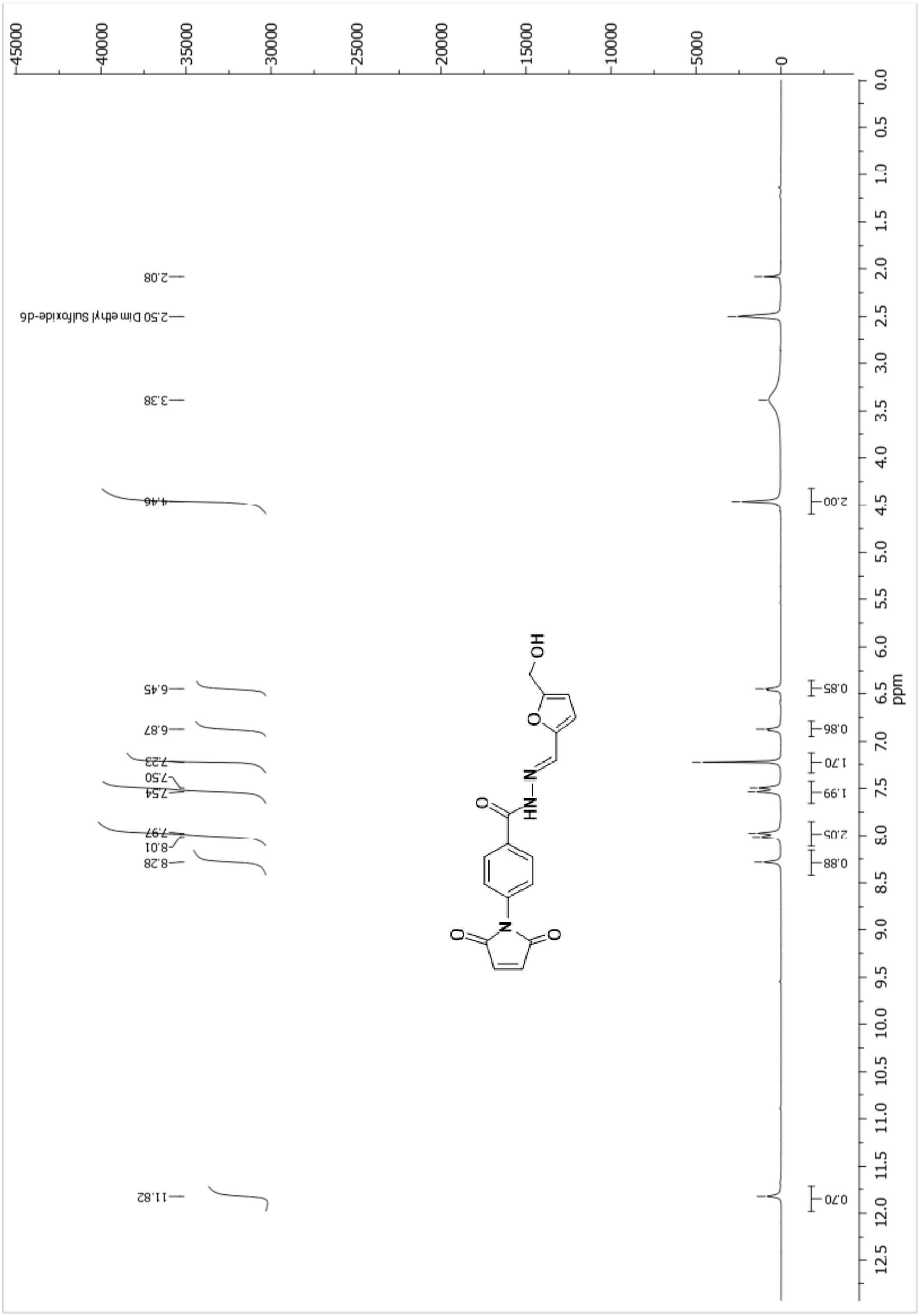

^{13}C NMR: 4-(2,5-dioxo-2,5-dihydro-1*H*-pyrrol-1-yl)-*N*-(5-(hydroxymethyl)furan-2-ylmethylene)benzohydrazide (**21**)

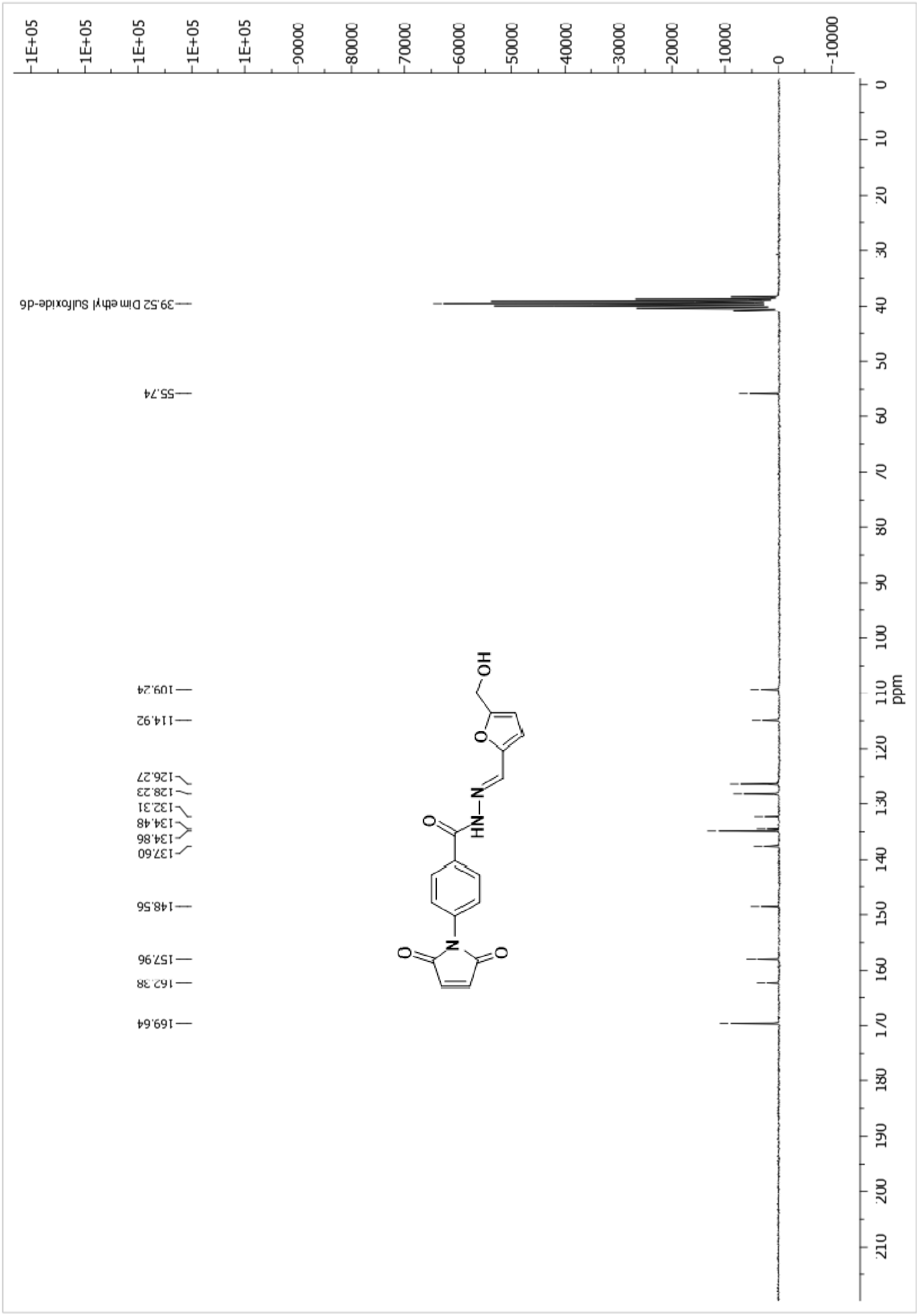

^{1}H NMR: 4-(2,5-dioxo-2,5-dihydro-1*H*-pyrrol-1-yl)-*N*-(5-(4-nitrophenyl)furan-2-ylmethylene)benzohydrazide (**22**)

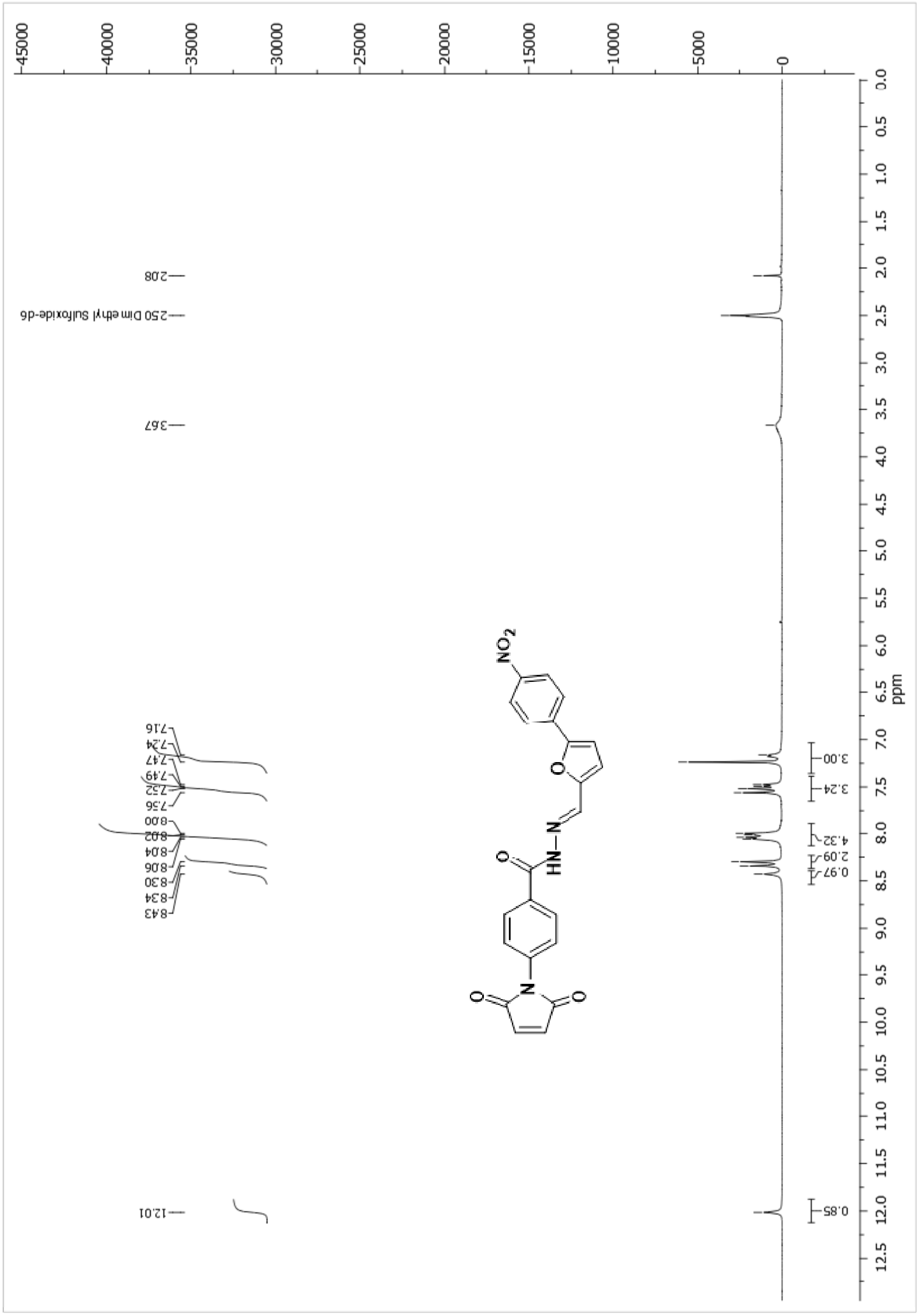

^{13}C NMR: 4-(2,5-dioxo-2,5-dihydro-1*H*-pyrrol-1-yl)-*N*-(5-(4-nitrophenyl)furan-2-ylmethylene)benzohydrazide (**22**)

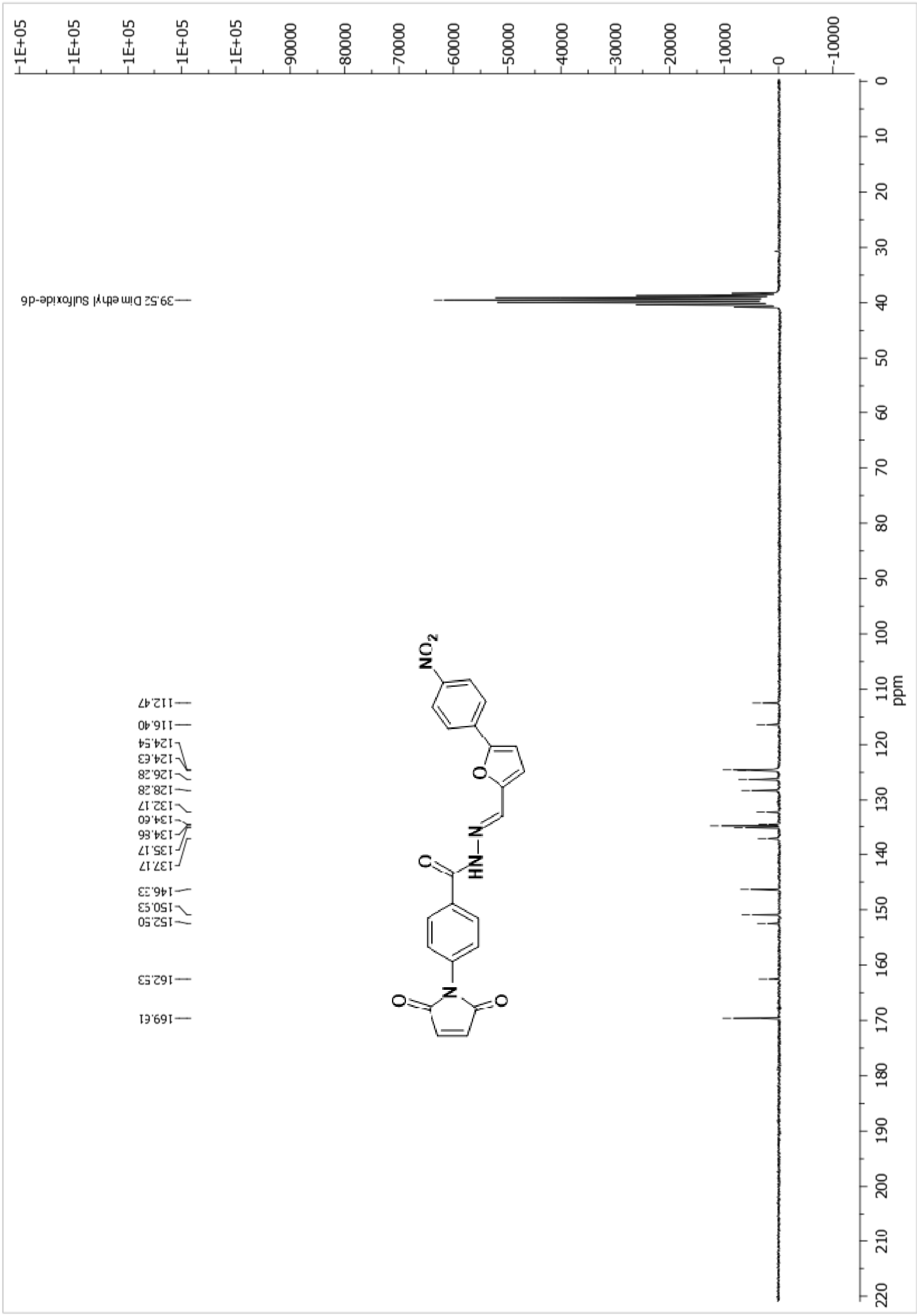

^{1}H NMR: 4-(2,5-dioxo-2,5-dihydro-1*H*-pyrrol-1-yl)-*N*-(pyridin-3-ylmethylene)benzohydrazide (**23**)

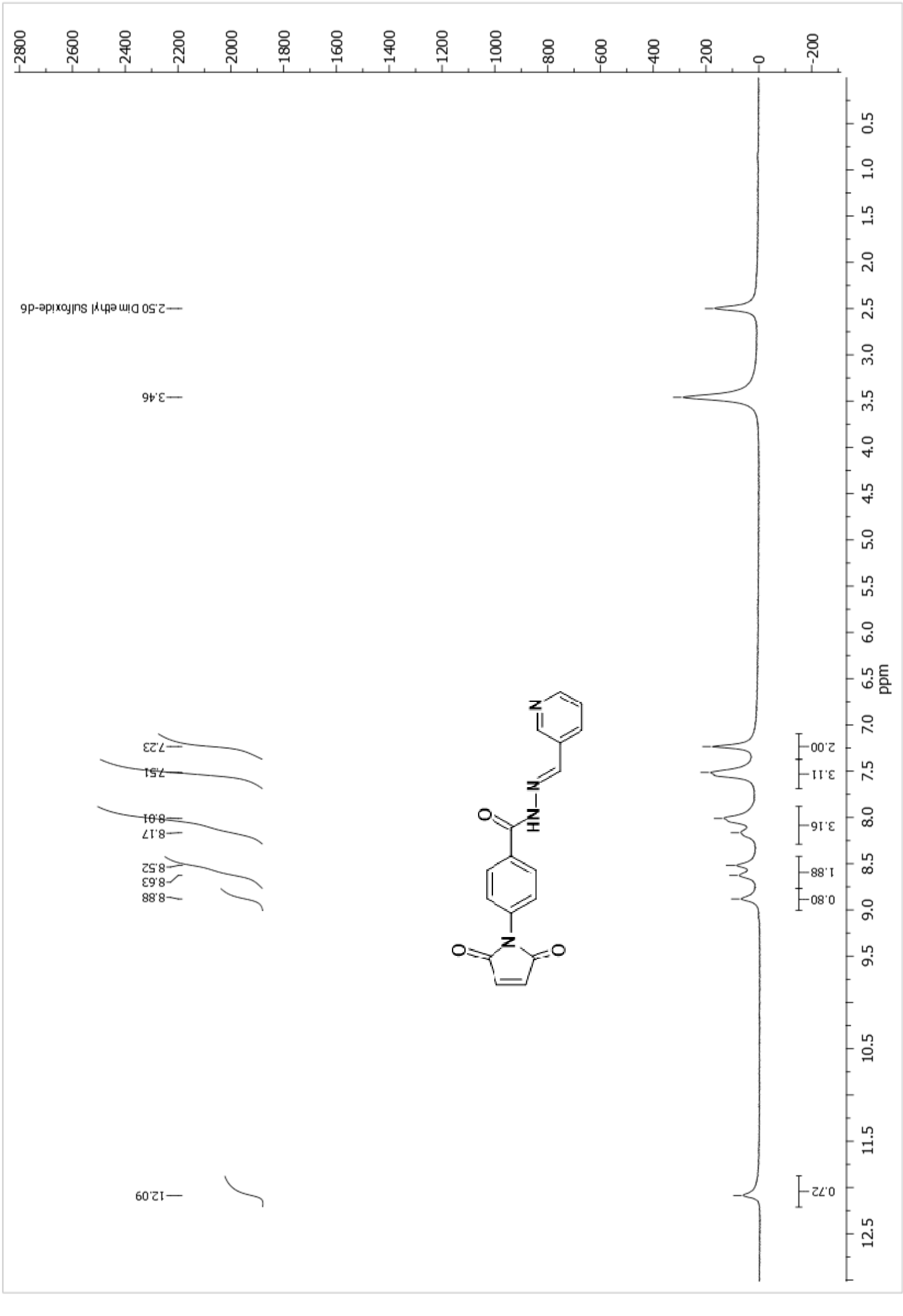

^{13}C NMR: 4-(2,5-dioxo-2,5-dihydro-1*H*-pyrrol-1-yl)-*N*-(pyridin-3-ylmethylene)benzohydrazide (**23**)

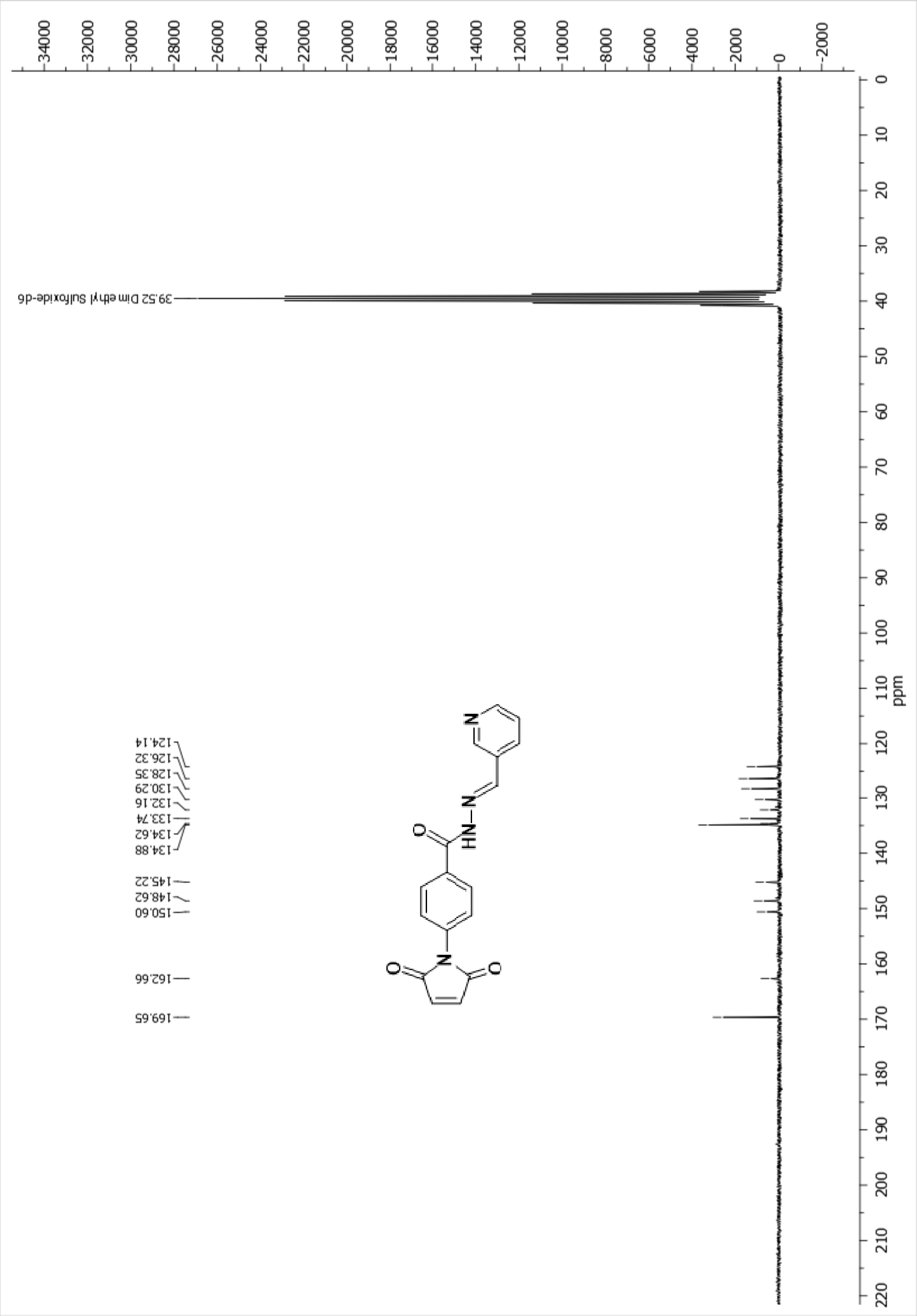

Supplement: Supplementary file 1 [file molecules-30-04035-s001.zip › Revised- no red color-SM-molecules-3815677.pdf]
